# Potential Direct Regulators of the *Drosophila yellow* Gene Identified by Yeast One-Hybrid and RNAi Screens

**DOI:** 10.1534/g3.116.032607

**Published:** 2016-08-12

**Authors:** Gizem Kalay, Richard Lusk, Mackenzie Dome, Korneel Hens, Bart Deplancke, Patricia J. Wittkopp

**Affiliations:** * Department of Molecular, Cellular, and Developmental Biology, University of Michigan, Ann Arbor, Michigan 48109; † Department of Ecology and Evolutionary Biology, University of Michigan, Ann Arbor, Michigan 48109; ‡ Institute of Bioengineering, School of Life Sciences, Ecole Polytechnique Fédéral de Lausanne, CH-1015 Lausanne, Switzerland

**Keywords:** transcription factor, pigmentation, ecdysone, Hr78, Hr38, Mutant Screen Report

## Abstract

The regulation of gene expression controls development, and changes in this regulation often contribute to phenotypic evolution. *Drosophila* pigmentation is a model system for studying evolutionary changes in gene regulation, with differences in expression of pigmentation genes such as *yellow* that correlate with divergent pigment patterns among species shown to be caused by changes in *cis*- and *trans*-regulation. Currently, much more is known about the *cis*-regulatory component of divergent *yellow* expression than the *trans*-regulatory component, in part because very few *trans*-acting regulators of *yellow* expression have been identified. This study aims to improve our understanding of the *trans*-acting control of *yellow* expression by combining yeast-one-hybrid and RNAi screens for transcription factors binding to *yellow cis*-regulatory sequences and affecting abdominal pigmentation in adults, respectively. Of the 670 transcription factors included in the yeast-one-hybrid screen, 45 showed evidence of binding to one or more sequence fragments tested from the 5′ intergenic and intronic *yellow* sequences from *D. melanogaster*, *D. pseudoobscura*, and *D. willistoni*, suggesting that they might be direct regulators of *yellow* expression. Of the 670 transcription factors included in the yeast-one-hybrid screen, plus another TF previously shown to be genetically upstream of *yellow*, 125 were also tested using RNAi, and 32 showed altered abdominal pigmentation. Nine transcription factors were identified in both screens, including four nuclear receptors related to ecdysone signaling (*Hr78*, *Hr38*, *Hr46*, and *Eip78C*). This finding suggests that *yellow* expression might be directly controlled by nuclear receptors influenced by ecdysone during early pupal development when adult pigmentation is forming.

Regulation of gene expression is important for the proper development and physiology of all organisms. Changes in gene expression contribute to phenotypic diversity within a species, as well as divergence between species (Stern and Orgogozo 2008; Martin and Orgogozo 2013). Such heritable changes in gene expression can result from changes in *cis*-regulatory sequences and/or *trans*-regulatory factors. *Cis*-regulatory sequences are typically located in noncoding regions of the genome and work by binding *trans*-regulatory molecules called transcription factors (TFs). TFs are typically proteins that recognize 6–12 bp long DNA sequences (Spitz and Furlong 2012). Physical interactions between a *cis*-regulatory sequence and one or more TFs largely determine when, where, and how much a gene is transcribed. Understanding how gene expression is controlled during development, and changes over evolutionary time, thus requires identifying both *cis*- and *trans*-regulatory factors, as well as the interactions between them.

To date, much more progress has been made studying *cis*-regulatory sequences of genes with divergent expression than TFs that bind to these *cis*-regulatory sequences (Wittkopp and Kalay 2012). This is largely because there has not been a practical way to systematically test TFs for binding to specific pieces of DNA. In recent years, however, yeast-one-hybrid (Y1H) systems have been developed that allow the entire repertoire of an organism’s TFs to be tested for evidence of binding to a particular DNA sequence (Simicevic and Deplancke 2010; Ouwerkerk and Meijer 2011; Reece-Hoyes and Walhout 2012). These Y1H assays use yeast cells as a host for both the putative *cis*-regulatory DNA sequence as well as each TF. When a TF binds to the region of DNA tested, transcription of a reporter gene occurs, allowing the yeast cells to grow on selective media. TFs that allow growth of the yeast cells are thus identified as possible direct regulators of the DNA-sequence being tested. A list of potential regulators from a Y1H screen can be narrowed further by identifying TFs that affect development of the trait of interest when their activity is reduced, such as with the use of RNA-interference (RNAi) (Hens *et al.* 2011).

Here, we describe complementary Y1H and RNAi screens designed to identify potential direct regulators of the pigmentation gene *yellow*. The *yellow* gene is required for black pigment formation in the genus *Drosophila* (Wittkopp *et al.* 2003), and its expression has diverged among species in a manner that correlates with pigmentation divergence (reviewed in Massey and Wittkopp 2016) (Figure 1). Both *cis*-regulatory (Wittkopp *et al.* 2002b; Gompel *et al.* 2005; Prud’homme *et al.* 2006; Jeong *et al.* 2006; Kalay and Wittkopp 2010; Ordway *et al.* 2014) and *trans*-regulatory (Wittkopp *et al.* 2002b; Werner *et al.* 2010; Arnoult *et al.* 2013) changes have been implicated in this expression divergence; *yellow* is also required for the wing extension behavior that is part of male courtship in *D. melanogaster* (Bastock 1956; Burnet *et al.* 1973), and expression required for this trait is regulated by a dedicated *cis*-regulatory element (Drapeau *et al.* 2006). For pigmentation, prior studies have identified the Abdominal-B (Abd-B) protein as a direct regulator of a male-specific body enhancer in *D. melanogaster yellow* (Jeong *et al.* 2006), as well as Engrailed (En) (Gompel *et al.* 2005) and Distal-less (Dll) (Arnoult *et al.* 2013) proteins as direct regulators of the wing enhancer in *D. biarmipes yellow*. Much less is known about the regulation of *yellow* expression for traits other than adult pigmentation, although the overexpression of Fruitless (Fru) protein, which is a major regulator of male courtship behavior (Anand *et al.* 2001), has been shown to induce Yellow protein expression in the larval brain (Drapeau *et al.* 2003). Identifying additional direct regulators of *yellow* will improve our understanding of how this critical pigmentation gene is regulated during development, and facilitate further investigation into how changes in this regulation have evolved.

**Figure 1:**
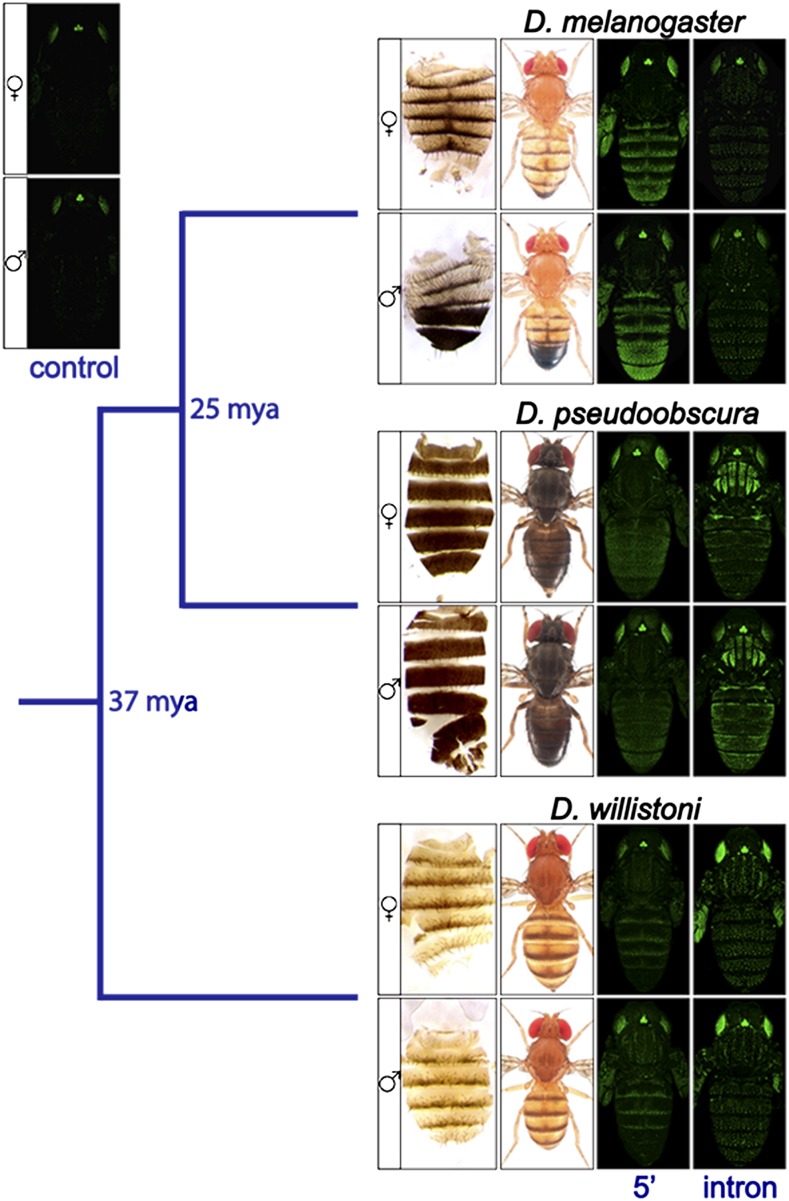
Differences in adult pigmentation patterns correlate with differences in *yellow* expression patterns between *Drosophila* species. Phylogenetic relationship between three species from the Sophophora subgenus, *D. melanogaster*, *D. pseudoobscura*, and *D. willistoni*, are shown. For each species, panels are as follows: from left to right, dissected dorsal abdomen, dorsal view of adult fly, pupal GFP expression in a *D. melanogaster* host driven by 5′ intergenic sequence upstream of *yellow*, pupal GFP expression in a *D. melanogaster* host driven by *yellow* intronic sequence, with females shown in the top row and males shown in the bottom row. Images of dorsal view of adult flies are courtesy of Nicolas Gompel. The panel labeled “control” shows pupal GFP expression in females (top) and males (bottom) driven by only the basal promoter used to construct all other reporter genes. The GFP used in these constructs is a nuclear enhanced green fluorescent protein (nEGFP). Additional information about these reporter genes can be found in Kalay and Wittkopp (2010), in which these images of adult flies and GFP expression patterns were first published.

## Materials and Methods

### Overview

**Figure 2:**
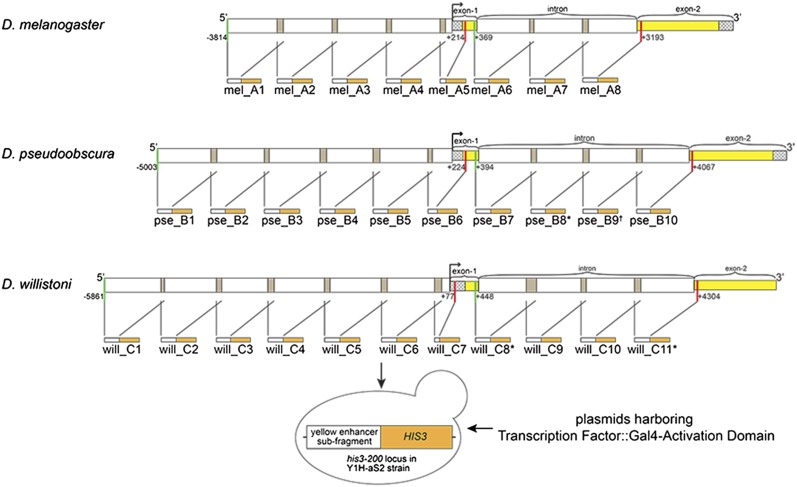
Fragments from *yellow* 5′ intergenic and intronic regions tested in yeast-one-hybrid (Y1H) assay. The *yellow* locus, including the 5′ intergenic and intronic regions from *D. melanogaster*, *D. pseudoobscura*, and *D. willistoni* are shown. Each locus was divided into the fragments indicated by the white boxes, plus the preceding and following gray shaded boxes (if applicable), which show the overlapping regions between neighboring fragments. The broken arrow represents transcription start site (TSS). Checkered boxes represent 5′ untranslated region (UTR) if they come before exon 1, 3′ UTR if they come after exon 2. Yellow boxes indicate *yellow* coding sequences. Green vertical lines indicate the start, and red vertical lines represent the end, of 5′ intergenic or intronic regions of *yellow* from each species. The numbers next to green and red vertical lines indicate the start and end position of 5′ intergenic and intronic regions relative to TSS. Each fragment was cloned in front of a *HIS3* reporter gene, which is represented by the orange boxes. The name of each fragment is written under each schematic. * indicates fragments that were not tested with Y1H for technical reasons, as described in *Materials and Methods*. † indicates that the fragment was tested with only half of the available transcription factors in Y1H, again for technical reasons. mel, *D. melanogaster*; pse, *D. pseudoobscura*; will, *D. willistoni*.

To identify potential direct regulators of *yellow*, we used a collection of 670 TFs from *D. melanogaster* (∼89% of all known and predicted TFs in this species) (Hens *et al.* 2011), and tested each one for evidence of binding to each of 26 overlapping fragments from the 5′ intergenic and intronic regions of *yellow* from *D. melanogaster*, *D. pseudoobscura*, and *D. willistoni*, which last shared a common ancestor 37 million yr ago (Russo *et al.* 1995) (Figure 1 and Figure 2). In *D. melanogaster*, the 5′ intergenic and intronic regions drive expression of *yellow* in the developing body, wings, bristles, larval mouthparts, and denticle belts (Geyer and Corces 1987; Martin *et al.* 1989; Wittkopp *et al.* 2002b; Jeong *et al.* 2006; Kalay and Wittkopp 2010). The 5′ intergenic region also harbors a *cis*-regulatory element required for proper male courtship behavior (Drapeau *et al.* 2006). *yellow* is also expressed in the embryo (Walter *et al.* 1991) and larval brain (Radovic *et al.* 2002) of *D. melanogaster*, although specific *cis*-regulatory elements driving these expression patterns have not been identified. Prior work has shown that the 5′ intergenic and intronic regions from *D. pseudoobscura* and *D. willistoni* also contain *cis*-regulatory sequences that drive pupal expression when assayed in *D. melanogaster* transformant flies (Figure 1; Kalay and Wittkopp 2010). Results from the biochemical Y1H screen for TF binding to these enhancers were combined with results from a genetic screen using RNAi to knock down activity of a subset of TFs and test for changes in pigmentation.

### Subcloning regions of yellow noncoding sequences

Fragments from the 5′ intergenic and intronic regions of *yellow* from *D. melanogaster*, *D. pseudoobscura*, and *D. willistoni* were amplified using PCR (see Supplemental Material, Table S1), and each subcloned into pGEMT, pDONR-P4-P1R, and pMW2 vectors. Each enhancer fragment was ∼ 1000 bp long, except for mel_A5, pse_B6, will_C7, which were 423 bp, 641 bp, and 345 bp long, respectively (Figure 2 and Table S1). Each fragment overlapped with the flanking fragments by ∼ 100 bp (Figure 2 and Table S1). A mix of *Taq* DNA polymerase and Phusion High-Fidelity DNA Polymerase (New England Biolabs) was used for PCR to minimize PCR-introduced mutations. PCR primers (see Table S1) had attB sequences attached to their 5′ ends that were compatible with the attP site in the pDONR-P4-P1R vector (Deplancke *et al.* 2004). PCR products were first subcloned into a pGEMT vector (Promega) for sequencing. Sequence-confirmed-fragments (see File S1) were cut out of pGEMT vector and subcloned into the pDONR-P4-P1R vector using a BP reaction to create an Entry clone (Gateway, Life Technologies). For each enhancer fragment, an LR reaction (Gateway, Life Technologies) was then used to move the fragment from pDONR-P4-P1R vector into the Y1H compatible pMW2 vector upstream of a basal promoter and coding sequence for the *S. cerevisiae HIS3* gene. These final constructs were mini-prepped and transformed into the Y1H-aS2 strain of *S. cerevisiae* using the lithium acetate (LiAc)–polyethylene glycol (PEG) method (Gietz and Woods 2006), where they were integrated into the mutant *his3-200* locus. Three out of the 29 enhancer/bait fragments subcloned into pMW2vector (pse_B8, will_C8 and will_C11) (Figure 2, File S1, and Table S1) could not be integrated into the yeast genome because the restriction enzyme used to linearize the vector harboring the DNAbait::*HIS3* fusion construct prior to genome integration also cut within the bait fragment. These three fragments were thus excluded from our analysis. Transformant cells for each of the other 26 baits were selected on synthetic complete medium lacking histidine (SC –His), which allowed growth only of cells that had incorporated the DNAbait::*HIS3* fusion construct into their genomes. This protocol follows that described in Hens *et al.* (2011).

### Yeast-one-hybrid (Y1H) assay

Prior to testing for interactions between specific TFs and specific DNA sequences, a self-activation test was conducted for each of the 26 integrated DNA sequence baits to determine whether the endogenous *S. cerevisiae* transcription factors could activate sufficient expression of the *HIS3* reporter gene to allow growth in the absence of any *D. melanogaster* TF. This test was performed by spotting eight independent transformants for each bait onto SC –His plates containing varying concentrations (0, 10, 20, 40, 60, 80, and 100 mM) of 3-amino-1,2,4-triazole (3AT), which is a competitive inhibitor of the enzyme imidazoleglycerol-phosphate dehydratase, encoded by the *HIS3* gene. This enzyme catalyzes the sixth step of histidine production (Fink 1964). The higher the level of 3AT in the medium, the more *HIS3* gene product is required for growth, resulting in a more stringent assay for TF binding (Reece-Hoyes and Walhout 2012). For each DNA sequence bait, a single transformant that was unable to grow on plates with 10, 20, and/or 40 mM of 3AT was selected and used for the subsequent Y1H screen.

**Figure 3:**
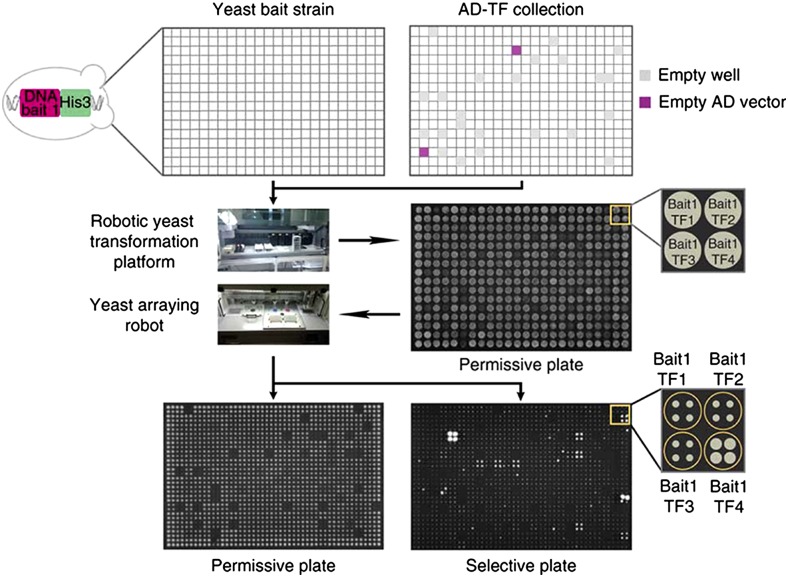
Overview of high-throughput Y1H assay. Each yeast strain with a *yellow* enhancer fragment (DNA bait)-*HIS3* reporter construct integrated into its genome (Yeast bait strain) was transformed with a library of transcription factors (TFs) fused to a Gal4 activation domain (AD-TF collection) using 384-well plates. As negative controls, each TF plate contained empty wells (gray squares in AD-TF collection plate) as well as constructs with activation domains (AD) without a TF attached (magenta squares in AD-TF collection plate) to test for activation in the absence of a TF. Then, each “bait-TF” combination was quadruplicated using a yeast arraying robot, first onto a permissive plate (no 3AT), then onto selective plates with increasing amounts of 3AT. After 3 d of growth, the permissive plate (no 3AT) was imaged as a reference. After 7 and then 10 d of growth, the selective plates were imaged and colony growth was scored as a readout of DNA bait–TF interaction. This figure is modified from Hens *et al.* (2011).

A previously constructed collection of 670 *D. melanogaster* TFs fused to the activation domain of the yeast Gal4 protein (GAL4-AD) arrayed across two 384-well plates (Hens *et al.* 2011) was used in this study. This collection includes ∼89% of all predicted and known TFs in *D. melanogaster*. Each of these 670 TFs (prey) were combined with each of the 26 *yellow* fragments (bait) in a 384-well format using the LiAc–PEG method, and plated on plates with synthetic complete medium lacking histidine, and tryptophan (SC –His, –Trp), which ensured growth of successful transformations only. After 3 d of growth, these 384-well format permissive plates were used to generate at least four 1536-well format plates by spotting each transformant genotype four times in a quartet of neighboring wells (Figure 3). One of the four 1536-well SC –His, –Trp plates did not contain any 3AT. The rest of the 1536-well SC –His, –Trp plates contained increasing concentrations of 3AT, starting with the lowest concentration of 3AT that prevented growth in the self-activation test. All plates were incubated at 30°C to allow colony growth, and then imaged using a Bio-Rad Gel Documentation camera. The permissive 1536-well plates lacking 3AT were imaged after 3 d of growth as positive controls, whereas the 1536-well plates containing 3AT were imaged after 7 and 10 d of growth. Two types of negative controls were included on each plate: cells that were not transformed with any TF-GAL4-AD construct (*i.e.*, an empty well in the AD-TF collection), and cells that were transformed with the Gal4-AD construct alone lacking any *D. melanogaster* TF. The former was used to test if endogenous yeast TFs can activate *HIS3*, and the latter was used to test whether Gal4-AD can bind to the bait DNA in the absence of a TF and activate *HIS3*. Sets of quadruplicate cells containing the same TF and bait DNA that showed growth above background levels were inferred to have a direct interaction between the *D. melanogaster* TF protein and the bait DNA that caused the cells to express the *HIS3* gene (Figure 3). One of the two plates of TFs was ultimately excluded for the pse_B9 bait fragment because of contamination, resulting in only ∼50% of the 670 TFs tested for binding to this sequence.

### Analysis of Y1H data

To determine which TFs showed evidence of binding to *yellow* enhancer fragments, the images from each 1536-well plate were analyzed using an R package called Gitter (Wagih and Parts 2014)—an image analysis tool for processing of colony-based screens. Raw plate images used for this analysis are included as File S2. This program detects the grid of colonies on each plate, and then uses the contrast between the colony and the medium to determine the area of the colony, the degree to which the colony is circular, and whether the colony appears to overlap with other colonies. This method was used as an alternative to the TIDY analysis pipeline for Y1H data described in Hens *et al.* (2011), which identified significant interactions much more liberally.

To ensure optimal processing, plate images were first manually cropped to remove the plate edges, which sometimes created glare that interfered with Gitter’s grid detection. Contaminant colonies, visible on some plates after 7 or 10 d of growth (see File S3), were also manually removed from the images at this stage by replacing them with gray regions of equal darkness to neighboring noncolony pixels. Gitter was then run in batch mode across all of these preprocessed images using default parameters with the exception of setting the ‘fast’ image scaling parameter to 3000, and the ‘autorotate’ parameter to ‘T.’

Each potential TF-enhancer interaction was represented on each plate by a quartet of colonies. Colonies flagged by Gitter as noncircular were screened from further analysis. Areas of colonies were calculated as the natural log of the number of pixels detected to be in the colony. Quartets were excluded from further analysis if they: (a) had only one detectable circular colony, (b) had mean area of < 2, or (c) had an index of dispersion among the quartet colony areas of > 0.2. The value of each quartet was recorded as the mean of the quartet’s colonies’ areas.

Z-scores were assigned to each quartet by comparing the quartet’s value to an outlier-screened and edge-corrected distribution of values of quartets across the plate. Outlier screening was performed by using Grubb’s test for outliers on the quartet values. To detect if the plate showed an edge effect, a *t*-test was used to compare the values of quartets in the outermost two rows and columns against the values of the inner quartets. If there was a significant difference between edge and interior quartets (alpha = 0.05), the values of the exterior quartets were adjusted downward by the difference between the mean values of edge and interior quartets. Z-scores were then calculated for each quartet, including outlier quartets, on the basis of these edge-corrected values. Y1H output files created by Gitter (see File S3) were analyzed using custom R scripts that are included in File S4. A complete summary of results is provided in Table S2, and with graphical summaries shown in the main text.

### RNAi screening

To identify TFs that affect pigmentation in adult *D. melanogaster* flies, we used RNAi to decrease TF activity, and examined body color in the dorsal abdominal cuticle. For this purpose, 153 RNAi lines were obtained from the Bloomington Drosophila Stock Center (BDSC), and one line (RNAi for *bric-a-brac*1) was obtained from Vienna Drosophila Stock Center (VDRC) (see Table S3). These lines were used to test the function of 124 different TFs that were also tested in the Y1H screen, as well as one transcription factor (*fru*) that was previously shown to be genetically upstream of *yellow* (Drapeau *et al.* 2003). The lines ordered from BDSC belong to the TRiP collection (Transgenic RNAi Project, Harvard Medical School), in which UAS-RNAi transgenes are integrated into an attP2 site on the third chromosome using the phiC31 site-directed integration (Ni *et al.* 2008, 2009). The integration of each of these UAS-RNAi transgenes into the same genomic location reduces variability in expression among these lines by controlling for position effects. RNAi lines targeting pigmentation genes *ebony*, *yellow*, and *tan* were also included as controls. The *ebony* RNAi line (part of TRiP collection) was ordered from the BDSC, and the *yellow* and *tan* RNAi lines were ordered from the VDRC (see Table S3).

**Figure 4:**
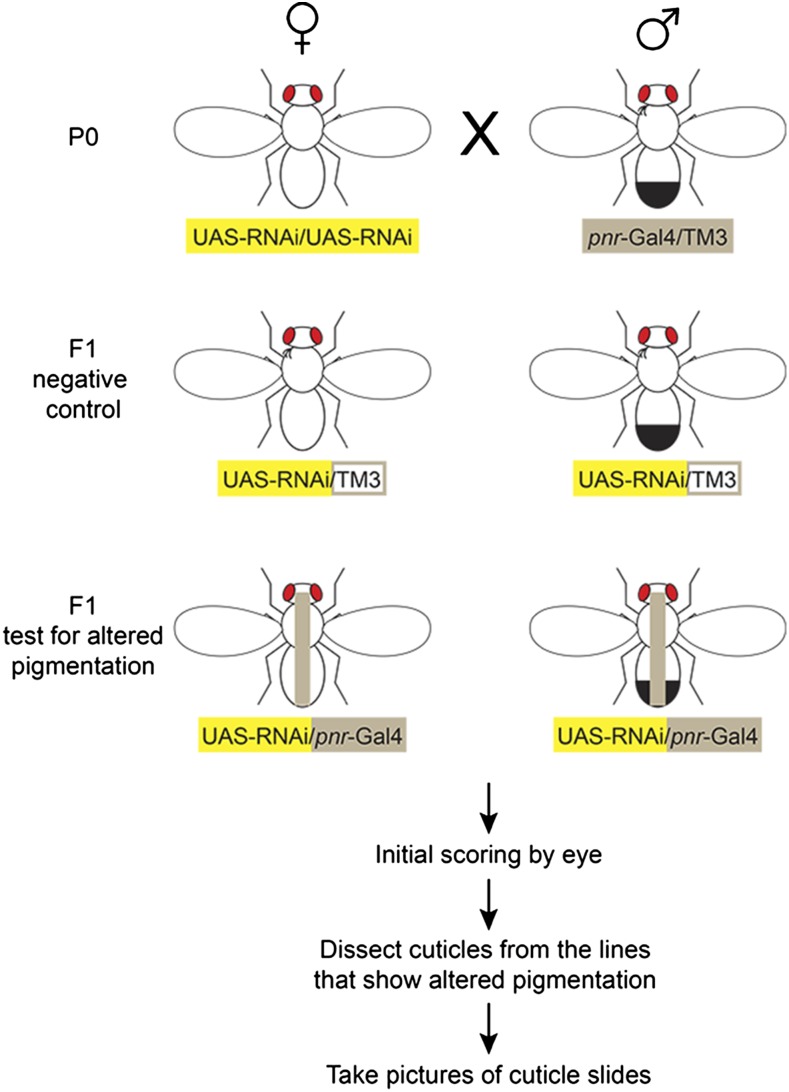
Overview of RNAi screen for transcription factors affecting pigmentation in *D. melanogaster*. To determine whether a given TF affects abdominal pigmentation in *D. melanogaster*, one or more homozygous UAS-RNAi lines reducing activity of that TF were each crossed to a line heterozygous for the *pannier*-Gal4 (*pnr*-Gal4), which drives expression in the dorsal midline (gray stripe), and a TM3 balancer on the 3rd chromosome (P0 cross). Half of the F_1_ progeny inherited the UAS-RNAi construct and the TM3 balancer (control), whereas the other half inherited the UAS-RNAi construct and the *pnr*-Gal4 driver (knockdown). The presence or absence, respectively, of a bristle phenotype on the humeral (shoulder) region of adult flies caused by a mutation on the TM3 balancer chromosome was used to distinguish control and knockdown flies. After scoring initially for possible abdominal pigmentation differences by eye, cuticles were dissected from lines that showed potentially altered abdominal pigmentation. Dissected cuticles were mounted on microscope slides and imaged as described in *Materials and Methods*.

Flies homozygous for a UAS-RNAi transgene were crossed to flies heterozygous for the *pannier*-Gal4 driver (*pnr*-Gal4) and a TM3 balancer chromosome (Figure 4). Flies inheriting the *pnr*-Gal4 driver, which expresses Gal4 in the dorsal midline during pupal stages when adult pigmentation is developing (Calleja *et al.* 2000; Wittkopp *et al.* 2002a) (Figure 6A), were tested for effects on pigmentation by comparing their pigmentation to that of siblings that inherited the TM3 balancer chromosome. More specifically, virgin females from RNAi lines with the genotype *y^1^*, *sc^1^*, *v^1^*; *P{y+t7.7 = CaryP}attP2*, *P{UAS-RNAi y+ v+}* were crossed to males with the genotype *y^1^*, *w^1118^*; *P{w+mW.hs = GawB}pnr^MD237^/TM3*, *P{w^+mC^ = UAS-y.C}MC2*, *Ser^1^*. From their progeny, females with the genotype *y^1^*, *sc^1^*, *v^1^* /*y^1^*, *w^1118^* ; *P{y+t7.7 = CaryP}attP2*, *P{UAS-RNAi y+ v+}/ P{w+mW.hs = GawB}pnr^MD237^*, and males with the genotype *y^1^*, *sc^1^*, *v^1^*; *P{y+t7.7 = CaryP}attP2*, *P{UAS-RNAi y+ v+}/ P{w+mW.hs = GawB}pnr^MD237^*, were identified based on the lack of a humeral bristle phenotype caused by a mutation on the TM3 balancer chromosome. These flies, which inherited both the UAS-RNAi and *pnr-GAL4* transgenes, were the “test” flies. Their siblings, which had the genotype *y^1^*, *sc^1^*, *v^1^*/*y^1^*, *w^1118^* ; *P{y+t7.7 = CaryP}attP2*, *P{UAS-RNAi y+ v+}/TM3*, *P{w^+mC^ = UAS-y.C}MC2*, *Ser^1^* in females, and *y^1^*, *sc^1^*, *v^1^*; *P{y+t7.7 = CaryP}attP2*, *P{UAS-RNAi y+ v+}/TM3*, *P{w^+mC^ = UAS-y.C}MC2*, *Ser^1^* in males, and therefore inherited a UAS-RNAi transgene without the *pnr-Gal4* driver required for its expression, carried the humeral bristle phenotype and were used as controls. All flies were raised at room temperature (∼22°) on cornmeal and yeast-based medium; 42.5 liters of this medium contains 39 liters of water, 675 g of yeast (SafPro Relax+YF deactivated dry yeast from Lesaffre Yeast Corp.), 390 g of soy flour (ADM, Protein Specialties Division), 2850 g of yellow cornmeal, 225 g of agar (MoorAgar, Inc.), 3 liters of light corn syrup (Karo light syrup), and 188 ml of propionic acid. Adult progeny from each cross were collected the day that they emerged, sorted based on sex and humeral bristle phenotype, aged 3–5 d, and scored for pigmentation by eye. If a difference in pigmentation was detected by eye between the control and test genotypes under the microscope, the flies were placed in a 1:10 glycerol:ethanol solution. After being stored in the glycerol:ethanol mix at least for 3 d, abdominal cuticle of test and control flies from both sexes were dissected for each line, mounted in polyvinyl alcohol mounting medium (BioQuip), baked at 65° overnight, and imaged using a Schott Leica MZ6 stereoscope with camera and “Scion Visicapture” version 1.2 software under identical lighting conditions. To improve the consistency between digital images and visual observations, we decreased background color and increased contrast by applying color adjustment to all cuticle images in each panel using Adobe Photoshop CS6.

### Data availability

The authors state that all data necessary for confirming the conclusions presented in the article are represented fully within the article.

## Results and Discussion

### Y1H screen identifies potential direct regulators of the yellow gene

**Figure 5:**
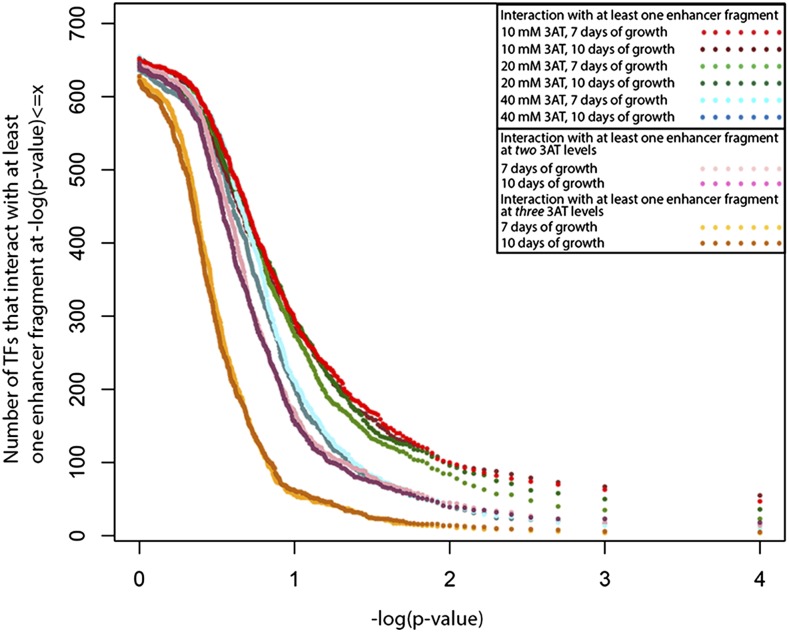
Effects of selection strength, incubation time, and significance threshold on interactions observed between TFs and *yellow* fragments. The number of TFs with a significant interaction with at least one *yellow* enhancer fragment (*y*-axis) at a given significance threshold or smaller [*x*-axis, –log(p-value)] is shown. The different colored dots show results using plates with different levels of inhibitor (10 mM, 20 mM, 40 mM of 3AT), two different incubation times (7 or 10 d of growth allowed), and when we required evidence of an interaction with at least one, two, or three levels of 3AT inhibitor, as indicated in the figure inset. Note that the rate of decrease in the number of TFs that showed a significant interaction with at least one *yellow* enhancer fragment slowed down around –log(p-value) = 2, which corresponds to p-value ≤ 0.01. Results discussed in the manuscript were based on a statistical threshold of p-value ≤ 0.005, or –log(p-value) = 2.3 and the requirement that a given TF-enhancer fragment interaction was observed with at least two 3AT levels.

Each of the 670 TFs was tested for evidence of binding to each of the 26 *yellow* enhancer fragments from *D. melanogaster*, *D. pseudoobscura*, and *D. willistoni* for a total of 17,420 unique tests. As expected, the number of TFs showing evidence of a significant interaction with a DNA fragment in the Y1H screen depended upon the statistical threshold used to call significance and the stringency of selection used (controlled by altering the amount of 3AT in the media, see *Materials and Methods*). The number of TFs showing a significant interaction with at least one of the DNA fragments generally decreased as 3AT concentrations (and thus the strength of selection) increased, regardless of the significance threshold used (Figure 5). Increasing the number of days of growth allowed before scoring from 7 to 10 had little effect on the number of interactions detected (Figure 5). Requiring a significant interaction at two or three levels of 3AT tested for a particular enhancer/TF pair reduced the number of significant interactions at all p-value thresholds (Figure 5). For all conditions, the rate of decrease in the number of TFs that showed evidence of an interaction with increasing statistical stringency slowed around –log_10_(p-value) = 2, which corresponds to a p-value of 0.01 (Figure 5).

To generate a list of possible regulators, we used a significance threshold of p-value < 0.005, corresponding to 2.3 in Figure 5. We also required that a specific TF show evidence of an interaction with the same DNA fragment for at least two of the 3AT concentrations tested for that fragment after 7 d of growth. Using these criteria, we found that 45 (6.7%) of the 670 TFs tested showed evidence of binding at least one enhancer fragment (Table 1 and Table S2), with ∼9% of these TFs (four of 45) showing a significant interaction with more than one of the 26 *yellow* enhancer fragments (Table 1 and Table S2). Nineteen TFs showed interactions with at least one of the eight *yellow* enhancer fragments from *D. melanogaster*, seven TFs with at least one of the nine fragments from *D. pseudoobscura*, and 21 with at least one of the nine fragments from *D. willistoni*. The TF interacting with the highest number of fragments (6/26) was Hormone-receptor-like in 78 (Hr78), which has been shown to repress ecdysone signaling (Zelhof *et al.* 1995). This TF interacted with at least one enhancer fragment from each of the three species, suggesting that it might be a conserved regulator. *Hr78* (also known as *DHR78*) encodes a nuclear receptor involved in larval molting, particularly cuticle formation in the larval tracheal system, and its absence leads to larval death at the third instar larval stage (Astle *et al.* 2003; King-Jones and Thummel 2005). *CG8765*, Hormone-receptor-like in 38 (Hr38) (also known as *DHR38*), and *intermediate neuroblasts defective* (*ind*) were tied for the next most commonly binding TFs. They each showed evidence of interaction with two of the 26 fragments for at least two different 3AT levels. To the best of our knowledge, none of these three genes have previously been implicated in regulating *yellow* expression, and thus represent new candidates for direct regulators of *yellow*. The full list of TFs with the number of 3AT levels that showed a significant (p-value < 0.005) interaction with each DNA fragment is shown in Table S2. These genes include *Abd-B*, which was previously shown to directly bind to *D. melanogaster yellow cis*-regulatory sequences (Jeong *et al.* 2006), as well as *Doublesex* (*dsx*), which has been shown to directly regulate, along with *Abd-B*, another gene that impacts pigmentation, *bric-a-brac 1* (*bab1*) (Williams *et al.* 2008).

**Table 1:** TFs interacted with at least one yellow enhancer fragment in Y1H

Gene Name	*D. melanogaster*	*D. pseudoobscura*	*D. willistoni*
Hr78*^a^*^,^*^b^*	xxxx	x	x
CG8765			xx
Hr38*^a^*^,^*^b^*	xx		
ind			xx
ab	x		
Abd-B*^b^*			x
bigmax	x		
C15*^b^*			x
caup	x		
CG11085			x
CG1233	x		
CG1529	x		
CG1621		x	
CG1647	x		
CG17806			x
CG31666			x
CG33695	x		
CG5591	x		
CG7101	x		
CG7928			x
CG8216	x		
CG9437	x		
CG9650		x	
crm	x		
d4			x
dsx*^b^*			x
Eip78C*^a^*^,^*^b^*		x	
Gsc			x
Hey			x
HLH4C		x	
Hr46*^a^*^,^*^b^*		x	
Lim3*^b^*			x
NfI	x		
Oaz	x		
Oli		x	
otp			x
p53			x
pdm2			x
pfk	x		
pnt*^b^*			x
ro			x
slp2	x		
stwl	x		
Su(var)3-7			x
toe			x

All transcription factors that showed a significant interaction with at least one *yellow* enhancer fragment from the 5′ intergenic and/or intronic regions from *D. melanogaster*, *D. pseudoobscura*, and/or *D. willistoni* for more than one 3AT levels are shown. Each “x” indicates evidence of an interaction between the TF and a *yellow cis*-regulatory sequence from the species indicated. The number of “x”s indicated shows the number of *yellow* enhancer fragments a given TF interacted with for that species.

a TFs that are nuclear receptors.

b TFs showing altered abdominal pigmentation when knocked down with RNAi.

Y1H screens are perhaps the best method currently available to systematically search for TFs binding to a *cis*-regulatory sequence of interest, but they are known to have an important portion of false positives and false negatives (Deplancke *et al.* 2004; Hens *et al.* 2011). False positives can be technical, where the reporter gene becomes active independent of an interaction between a TF and a DNA fragment, or they can be biological, where a TF and DNA fragment that interacted in the yeast cell do not interact in their native biological setting for one of many reasons, including lack of competition among TFs (Walhout 2011). We have tried to minimize technical false positives by testing for self-activation of each DNA fragment and indiscriminate activation by the vector used to express TFs, assaying each potential interaction in quadruplicate, using stringent statistical thresholds, and requiring evidence of binding at multiple levels of inhibitor (3AT). Testing a TF-DNA fragment interaction at multiple 3AT levels is a “semi-independent” way of confirming a given interaction, because even though independent transformations are not conducted, the same transformant colony has to grow in a new, perhaps more stringent, environment. Presence of growth in multiple 3AT levels makes it more likely that a given TF-DNA fragment interaction is not a technical false positive. These stringent criteria may cause us to miss some true interactions, however, especially in cases where an interaction activates a low level reporter gene expression, such as when a repressor binds to the DNA fragment (Deplancke *et al.* 2004). These cases can be detected only at low levels of 3AT, where growth environment is less stringent. The raw Y1H data (plate images) are provided in File S2 so that other researchers can analyze them with different analysis criteria.

The false positive and false negative rates are unknown for our data, but a previous Y1H experiment using the same reagents as our study found that 72–77% of the TFs detected as binders of specific DNA fragments in a Y1H screen were also detected with a secondary direct binding assay (Hens *et al.* 2011). A false negative rate was also reported in this prior study, with 14 of the 19 previously reported direct regulators not detected using the automated Y1H system (Hens *et al.* 2011). Consequently, we anticipate that most of the TFs identified in our Y1H screen are likely to truly be able to bind to *yellow cis*-regulatory sequences in yeast at least, but many other factors might also bind that we failed to identify. We also anticipate that only a subset of TFs that are capable of binding to the sequences tested will actually regulate *yellow* expression in *D. melanogaster*. For these reasons, we think it is premature to interpret similarities and differences in TF binding among species, even thought this was one of the original goals of our experiment.

### RNAi screen identifies new regulators of adult pigmentation

**Figure 6:**
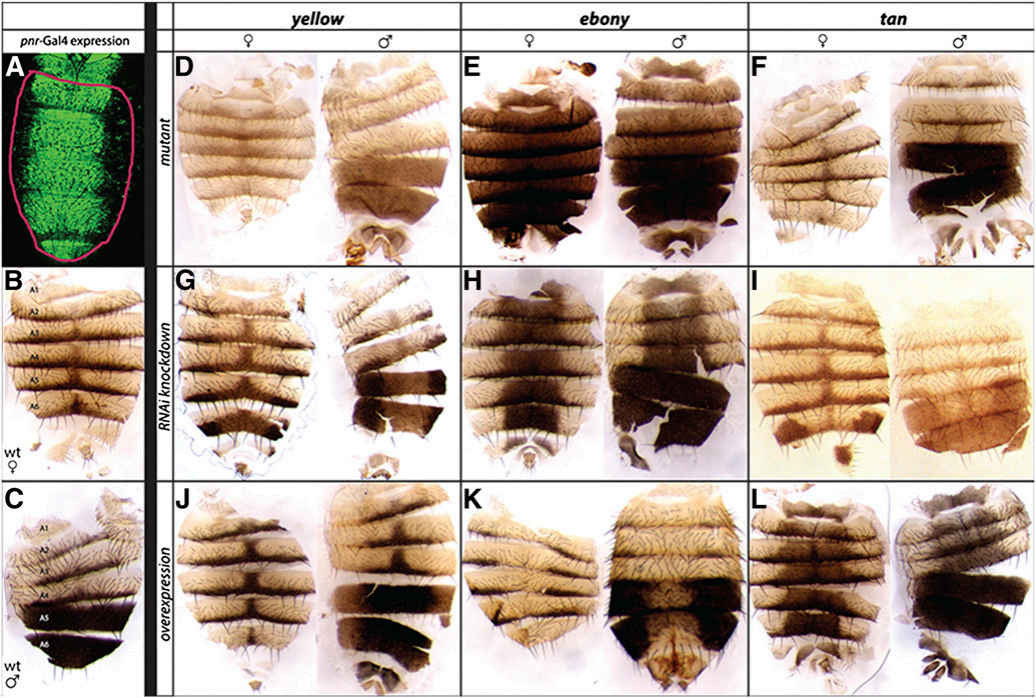
Altering expression of *yellow*, *tan*, and *ebony* in *D. melanogaster* alters pigmentation. For comparison to the effects of knocking down TFs with unknown effects, we examined the pigmentation phenotypes caused by altering genes required for pigment synthesis with our dissection and imaging protocols. Specifically, we examined the effects of altering the *yellow*, *ebony*, and *tan* genes, which are required for pigment synthesis (Massey and Wittkopp 2016). Changes in abdominal pigmentation were caused by loss-of-function mutations as well as Gal4-driven increases and decreases in expression for all three genes. The Gal4 driver used for this work was *pnr-Gal4*, which activates expression along the dorsal midline, as shown by a UAS-GFP construct in (A). Note that *pnr-Gal4* activates expression in a subset of the dorsal abdominal cuticle [outlined in red in (A)], allowing pigmentation outside of the *pnr-Gal4* expression domain to be used as an internal control within each cuticle. Wild-type *D. melanogaster* abdominal cuticle from females (B) and males (C) are also shown for comparison. (D–F) Loss-of-function mutations in *yellow* (D), *ebony* (E), and *tan* (F) altered pigmentation throughout the abdominal cuticle, with *yellow* mutants showing decreased black pigments, *ebony* mutants showing decreased yellow pigments, and *tan* mutants showing decreased brown pigments (G–I) Reducing activity of *yellow* (G), *ebony* (H), and *tan* (I) using RNAi caused similar changes in pigmentation, but only in the *pnr-GAL4* expression domain. (J–L) Increasing activity of *yellow* (J), *ebony* (K), and *tan* (L) in the *pnr-Gal4* expression domain had opposite effects on pigmentation. Images shown in this panel were taken on multiple days, causing imaging conditions to vary among genotypes.

As described in the introduction, *yellow* is a pleiotropic gene required for pigmentation in larvae and adults as well as mating behavior and potentially other traits (reviewed in Wittkopp and Beldade 2009). The *cis*-regulatory sequences controlling *yellow* expression during the pupal stage when adult pigmentation is forming have been studied most extensively (Geyer and Corces 1987; Martin *et al.* 1989; Wittkopp *et al.* 2002b; Jeong *et al.* 2006; Kalay and Wittkopp 2010), but potential direct regulators identified by the Y1H screen could affect any aspect of *yellow* expression. To identify TFs that might specifically affect *yellow* expression during the pupal stages and contribute to pigmentation development, we used UAS-RNAi transgenes (Ni *et al.* 2008, 2009) to reduce the activity of 124 of the 670 TFs tested in the Y1H screen (see Table S3) plus *fru*, which is genetically upstream of *yellow*, along the dorsal midline of the developing fly with a *pnr*-Gal4 driver (Calleja *et al.* 1996). We then looked for effects on pigmentation in dissected cuticle from adult abdomens by comparing flies with both the *UAS* and *Gal4* transgenes to their siblings that lacked one or both of these components (Figure 4). During pupal development, *pnr*-Gal4 is expressed in a stripe along the dorsal midline throughout the abdomen (Figure 6A), in cells that produce black, brown, and yellow pigments in both females (Figure 6B) and males (Figure 6C). As positive controls and reference points for this analysis, we also used *pnr*-Gal4 to modify activity of three genes required for pigment synthesis: *yellow*, *ebony*, and *tan*. *yellow* mutants lack black pigment (Figure 6D), *ebony* mutants lack yellow pigment (Figure 6E), and *tan* mutants lack brown pigment (Figure 6F). Reducing activity of these three genes in the *pnr*-Gal4 expression domain using UAS-RNAi resulted in a loss of the expected pigment for each gene (Figure 6, G–I), whereas overexpressing each of these genes using *pnr-Gal4* and UAS-*yellow*, UAS-*ebony*, or UAS-*tan* resulted in a gain of black, yellow, and brown pigments, respectively (Figure 6, J–L). Note that overexpression of *yellow* primarily darkens black pigmentation where it already exists (described in more detail in Wittkopp *et al.* 2002b) whereas overexpression of *ebony* or *tan* is sufficient to produce yellow or brown pigment, respectively, in all cells within the *pnr*-Gal4 expression domain.

**Figure 7:**
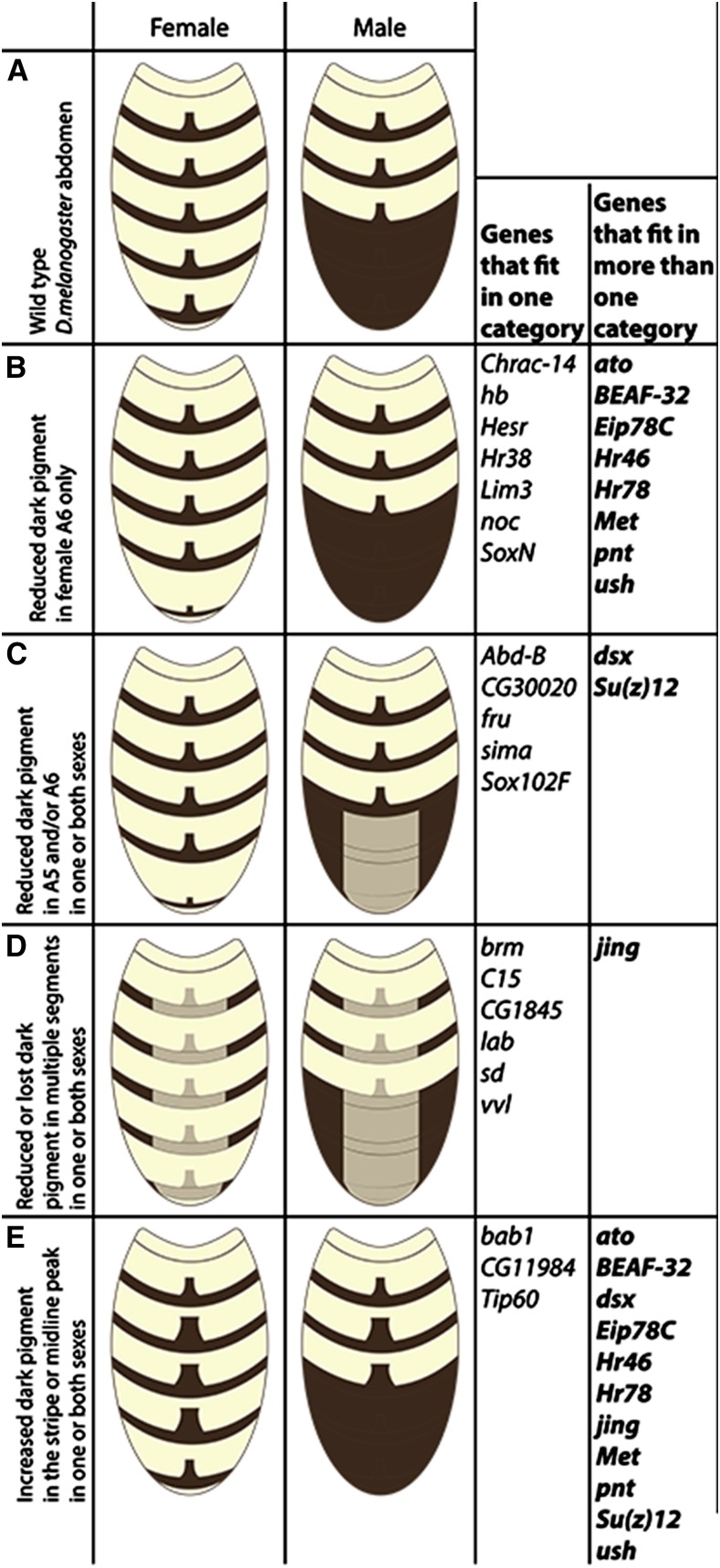
Summary of RNAi knockdown phenotypes observed for 32 transcription factors. Schematic representations of abdominal pigmentation in wild type *D. melanogaster* (A), and after knocking down activity of 32 transcription factors (B–E) are shown. Four classifications were used to describe the pigmentation phenotypes of RNAi knockdown flies: reduced dark pigment in female segment A6 only (B), reduced dark pigment in A5 and/or A6 in one or both sexes (C), reduced or lost dark pigment in multiple segments in one or both sexes (D), and increased dark pigment in stripes and/or midline peak in one or both sexes (E). Knockdown phenotypes of 21 transcription factors each fell into only one of these four categories, whereas knockdown phenotypes of 11 transcription factors were more complex and fell into more than one category.

Among the 125 RNAi lines targeting TFs we tested, we found that 32 (∼25%) affected abdominal pigmentation when activated by *pnr*-Gal4 (Figure 7, Table S4, and File S5). Reducing activity of seven of these TFs, *Chromatin accessibility complex 14kD protein* (*Chrac-14*), *hunchback* (*hb*), *Hes-related* (*Hesr*), *Hr38*, *Lim3* (*Lim3*), *no ocelli* (*noc*), and *SoxNeuro* (*SoxN*), reduced dark pigment only in the A6 abdominal segment of females (Figure 7B), whereas reducing activity of five other TFs *Abd-B*, *CG30020*, *fru*, *similar* (*sima*), *Sox102F* (*Sox102F*), resulted in reduced or partially lost dark pigment in the A5 and/or A6 abdominal segments of one or both sexes (Figure 7C). As previously shown (Celniker *et al.* 1990; Hopmann *et al.* 1995), males with *Abd-B* knocked down in the dorsal midline, lost male specific pigmentation in abdominal segments A5 and A6. The knock-down of the above 12 TFs had no noticeable effect on pigmentation in other segments. Six TFs reduced abdominal pigmentation in multiple segments when knocked down, with each having a unique effect (Figure 7D, Table S4, and File S5). Knock down of *brahma* (*brm*) resulted in loss of pigmentation in abdominal segments A2–A6 in males, including the midline peak, but had no visible effect on pigmentation in females. *C15*, when knocked down, resulted in loss of the midline peak in female abdominal segments A2–A6 and in male abdominal segments A2, A3, and A4, and also removed part of the dark pigmentation in the anterior part of male abdominal segment A5. This knockdown also led to overall thinner dark stripes in all segments that have it. Knockdown of *CG1845* resulted in close to complete loss of dark pigment in the dorsal midline of the dark stripe in female abdominal segments A2–A6 and abdominal segments A2, A3, and A4 in males. This knockdown also led to close to complete loss of the midline peak of all segments that normally have it, and it resulted in reduced dark pigment in the dorsal midline of male abdominal segments A5 and A6. Knock down of *labial* (*lab*) resulted in partial or complete loss of dark pigment in the dorsal midline of female abdominal segments A2–A6, and male abdominal segments A2, A3, and A4. This knockdown did not affect the midline peak of dark stripe in any of the segments except the female abdominal segment A6, where it resulted in a close to complete loss. Knock down of *scalloped* (*sd*) reduced dark pigment in male abdominal segments A2–A6, and led to close to complete loss of dark pigment in female abdominal segment A6. Finally, knock down of *ventral veins lacking* (*vvl*) resulted in reduced dark pigment in the *pnr*-Gal4 expression domain in abdominal segments A2–A6 in both males and females. Three TFs increased pigmentation in both males and females when knocked down (Figure 7E): Knockdown of *Tat interactive protein 60kda* (*Tip60*) and *CG11984* increased pigmentation by broadening the midline peak in abdominal segments A3–A6 in females and in abdominal segments A3 and A4 in males, whereas *bab1* broadened the midline peak in abdominal segments A2 and A3 of males and females, as well as led to gain of male specific pigmentation in female abdominal segments A4, A5, and A6 and male abdominal segment A4.

Interestingly, 11 of the TFs tested [atonal (ato), Boundary element-associated factor of 32kD (BEAF-32), dsx, Ecdysone-induced protein 78C (Eip78C), Hormone receptor 3 (Hr46), Hr78, jing (jing), Methoprene-tolerant (Met), pointed (pnt), Su(z)12 (Su(z)12), and u-shaped (ush)] had opposite effects on pigmentation in different segments of the abdomen or in different sexes when knocked down with RNAi (Figure 7, Table S4, and File S5). Consequently, they are listed in more than one category in Figure 7. For example, knockdowns of ato, Eip78C, Hr46, Hr78, Met, and ush increased dark pigmentation in female abdominal segments A3, A4, and A5, and male abdominal segments A3 and A4 (also A2 in the case of ush) (Figure 7E), but reduced or eliminated dark pigmentation in female abdominal segment A6 (Figure 7B). Reducing activity of Su(z)12 resulted in broader midline peaks in female abdominal segments A3, A4, and A5, and male abdominal segments A3 and A4 (Figure 7E), but less dark pigmentation in female abdominal segment A6, and male abdominal segments A5 and A6 (Figure 7C). Knockdown of pnt or BEAF-32 increased dark pigmentation in male abdominal segments A3 and A4 (Figure 7E), but also decreased dark pigmentation in female abdominal segment A6 (Figure 7B). Knockdown of jing resulted in reduced dark pigment in abdominal segments A2 through A6 in both sexes (Figure 7D), but this knockdown also increased the thickness of the pigmented stripe and broadened the midline peak in all female abdominal segments A2–A6 and male abdominal segments A3 and A4 (Figure 7E). Similar to Abd-B, when knocked-down, jing also resulted in loss of male-specific pigmentation in males. Finally, knocking down the activity of dsx resulted in gain of male specific pigmentation in female abdominal segment A6 (Figure 7E), but this knockdown also resulted in partial loss of dark pigment in male abdominal segments A5 and A6 (Figure 7C).

Two of the TFs that affected abdominal pigmentation in our RNAi screen are necessary for proper male courtship behavior in *D. melanogaster*. The first TF, *fru*, was not included in our Y1H screen, but has previously been shown to be genetically upstream of *yellow* in the 3rd instar larval brain (Radovic *et al.* 2002; Drapeau *et al.* 2003). *fru* has also been shown to be a master regulator of male courtship behavior in *D. melanogaster* (Anand *et al.* 2001)— a trait for which we know *yellow* is also necessary (Bastock 1956; Burnet *et al.* 1973). Mutants lacking the Fru zinc finger domain lack *yellow* expression in the 3rd instar larval brain (Drapeau *et al.* 2003). According to the summary of mutant phenotypes on FlyBase (Attrill *et al.* 2016), effects of *fru* on adult body pigmentation have not been described. We found that when *fru* was knocked down in *D. melanogaster* using the *pnr-Gal4* driver, dark pigment was almost completely lost in the dorsal midline of abdominal segment A6 in females, and was reduced in abdominal segment A5 of both sexes (Figure 7C, Table S4, and File S5). The second TF, *ato*, was tested with Y1H, but did not show a statistically significant interaction with any of the *yellow* enhancer fragments with the inclusion criteria and significance thresholds used in our analysis. Knocking down *ato* in the RNAi screen, however, reduced pigmentation in female abdominal segment A6, and increased pigmentation in the midline peak of female A3, A4, A5, and male A3 and A4 abdominal segments (Figure 7, B and E, Table S4, and File S5). Interestingly, and similar to *fru* and *yellow*, this gene is also necessary for proper male mating behavior, specifically the production of courtship song (Tauber and Eberl 2001). These observations suggest that the pathways regulating pigmentation and male courtship might have more shared components than currently appreciated. For a more detailed description of all pigmentation phenotypes observed in this study, please refer to Table S4.

Three of the 32 TFs that affected adult abdominal pigmentation in our RNAi screen have previously had their effects on pigmentation described in detail: *Abd-B* (Jeong *et al.* 2006), *dsx* (Williams *et al.* 2008), and *bab-1* (Kopp *et al.* 2000; Williams *et al.* 2008). Three more of these TFs (*Sox102F*, *jing*, and *vvl*) were also described as having effects on pigmentation in another recent screen that used a *pnr*-Gal4 driver and UAS-RNAi lines from the TRiP collection (Rogers *et al.* 2013). Additionally, overexpression of *ush* in the thorax has been shown to cause pigmentation to darken (Calleja *et al.* 2002), and localized knockdowns of *Hr78* and *Hr46* have previously been described as affecting abdominal pigmentation (Lam *et al.* 1999). The remaining 23 TFs [*ato*, *BEAF-32*, *brm*, *C15*, *CG1845*, *CG11984*, *CG30020*, *Chrac-14*, *Eip78C*, *fru*, *hb*, *Hesr*, *Hr38*, *lab*, *Lim3*, *Met*, *noc*, *pnt*, *sd*, *sima*, *SoxN*, *Su(z)12*, and *Tip60*] are, to the best of our knowledge, implicated in development of adult body pigmentation for the first time here, although *Hr38* has previously been shown to affect immune-related melanization (Sekine *et al.* 2011). With the exception of *Tip60*, *CG11984*, and *Chrac-14*, these 23 TFs plus *ush*, *Hr78*, and *Hr46* were also tested for effects on pigmentation in Rogers *et al.* (2013), but reported not to affect pigmentation. This difference in interpretation likely resulted from the fact that we scored pigmentation phenotypes after dissecting and mounting abdominal cuticles, whereas Rogers *et al.* (2013) scored pigmentation by looking at whole flies; the phenotypes we observed in *pnr-Gal4;UAS-RNAi* lines for these TFs were subtle, and likely not visible without dissection. In all, 115 of the 125 TFs we tested in our RNAi screen were also tested in Rogers *et al.* (2013), with six (*Abd-B*, *dsx*, *bab1*, *jing*, *Sox102F*, and *vvl*) found to affect adult abdominal pigmentation in both screens, 23 found to affect pigmentation only in our study, and 86 found to have no effect on adult abdominal pigmentation in either study (see Table S2, Table S3, and Table S4).

Nine genes (*Abd-B*, *dsx*, *C15*, *Eip78C*, *Hr38*, *Hr46*, *Hr78*, *Lim3*, and *pnt*) with effects on pigmentation in our RNAi screen also showed a significant biochemical interaction with at least one *yellow* enhancer fragment in our Y1H screen (Table 1, Table S2, and Table S4), suggesting that they might be direct regulators of *yellow*. The other TFs with effects on pigmentation described above might alter body color by affecting expression and/or activity of pigmentation genes other than *yellow* or might directly regulate *yellow* but not affect pigmentation because of shadow enhancers, but the high false negative rate of Y1H screens and the stringent criteria we used when analyzing the Y1H data prevent us from excluding these 23 genes as regulators of *yellow*. Additional genetic and biochemical experiments are needed to test these hypotheses. However, a recent study comparing evidence of TF binding and phenotypes resulting from reducing activity of these TFs on development of the *Caenorhabditis elegans* intestine also found very limited overlap between these sets of genes, suggesting that the phenotypic effects of many TFs are indirect (MacNeil *et al.* 2015).

### Nuclear receptors and ecdysone signaling: direct regulators of yellow expression and pigmentation?

**Figure 8:**
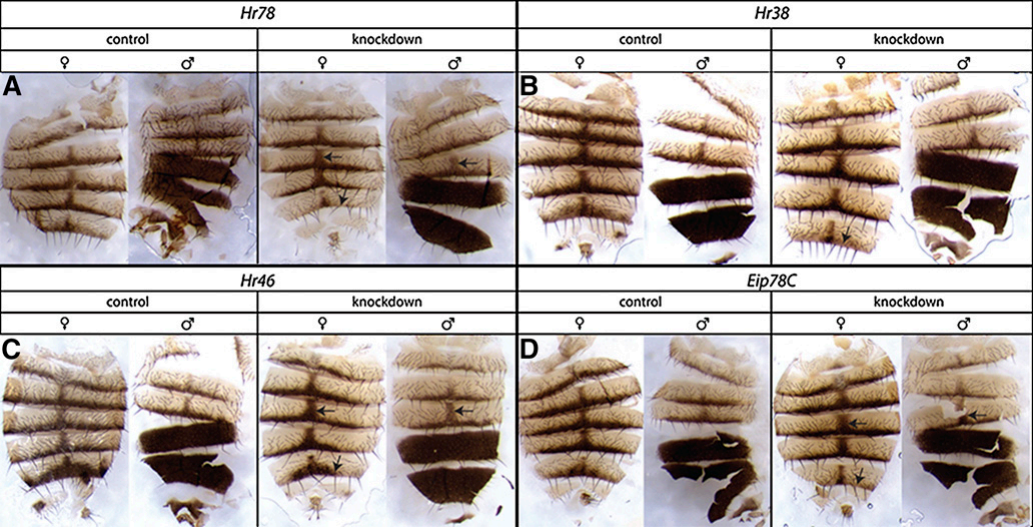
RNAi knockdown phenotypes for nuclear receptor genes showing evidence of binding to *yellow* fragments in yeast-one-hybrid screen. Abdominal pigmentation phenotypes in both control and knockdown flies of both sexes are shown for four nuclear receptors, *Hr78*, *Hr38*, *Hr46*, and *Eip78C*. Changes in pigmentation observed consistently in knockdown relative to control flies are marked with arrows and described further in Table S4. Briefly, knockdown of *Hr78*, *Hr46*, and *Eip78C* resulted in increased dark pigment in female segments A3, A4, A5, and male segments A3 and A4 as well as reduced dark pigment in female segment A6. Knockdown of *Hr38* only led to reduced dark pigment in female segment A6.

The most striking pattern to emerge from our Y1H and RNAi screens is the high proportion of nuclear receptors related to ecdysone signaling identified as potential direct regulators of *yellow*. We tested 11 of the 18 nuclear receptors in the *D. melanogaster* genome (King-Jones and Thummel 2005) for evidence of binding to *yellow cis*-regulatory sequences in our Y1H screen, and found that four of them (*Hr78*, *Hr38*, *Hr46*, and *Eip78C*) (∼36%) were among the 45 TFs that showed a statistically significant interaction with at least one *yellow* enhancer subfragment tested (Table 1 and Table S2). Two of these receptors, *Hr78* and *Hr38*, were among the four TFs with evidence of binding to more than one *yellow* fragment tested (Table 1 and Table S2). All four of these nuclear receptors were also found to alter pigmentation when their activity was reduced using RNAi (Figure 8 and File S5). Another nuclear receptor, *Hr4*, which was not included in our study, was also reported to have effects on pigmentation when activity was reduced by RNAi in Rogers *et al.* (2013). Taken together, these data suggest that one or more of these nuclear receptors might directly regulate *yellow* expression. *Hr78* showed evidence of binding to fragments from all three of the species tested, whereas each of the other three nuclear receptors (Eip78C, Hr46, and Hr38) showed evidence of binding to one or more *yellow* fragments in only one of the three species tested. These differences in binding among species might result from false positives and/or negatives, but could also be caused by developmental systems drift or divergent activities.

Nuclear receptors respond to a ligand by translocating to the nucleus and directly regulating transcription of target genes. The ligands for most nuclear receptors in *D. melanogaster* have not been identified, but many appear to be affected by ecdysone signaling, which controls development and metamorphosis (King-Jones and Thummel 2005). *Hr38* can even substitute for the ecdysone receptor (EcR) (Riddiford *et al.* 2000). Seven of the 18 nuclear receptors are transcriptionally regulated by the active form of ecdysone, hydroxyecdysone (20E), including the other three nuclear receptors showing evidence of binding to *yellow* sequences in our Y1H screen (*Hr78*, *Hr46*, and *Eip78C*) (King-Jones and Thummel 2005). During metamorphosis, ecdysone signaling controls processes such as tissue-specific cell proliferation, differentiation and programmed cell death, reproductive and behavioral changes, and cuticle deposition (Thummel 1995; King-Jones and Thummel 2005). This last function is particularly intriguing given that (i) *yellow* is expressed during pupal development at the same time that deposition of the adult cuticle begins (Walter *et al.* 1991); (ii) Yellow protein is exported from the epidermal cells and incorporated into the developing cuticle (Walter *et al.* 1991; Wittkopp *et al.* 2002a); and (iii) *yellow* mutants show significant changes, both increase and decrease, in the amount of molecules involved in chitin biosynthesis, which is necessary for proper cuticle formation and pigmentation (Bratty *et al.* 2012). Indeed, in the swallowtail butterfly, *Pailio xuthus*, a link between ecdysone signaling and *yellow* expression was established by showing that exposure to a high titer of 20E increased *yellow* expression (Futahashi and Fujiwara 2007).

Additional support for the hypothesis that one or more of these nuclear receptors affect pigmentation, and might do so by regulating *yellow* expression, comes from prior studies of these genes. For example, *Eip78C*, *Hr46* (also known as *DHR3*), and *Hr4* are all expressed early in pupal development at the end of the ecdysone peak (King-Jones and Thummel 2005), shortly before *yellow* expression begins (Walter *et al.* 1991). Localized knockdowns of *Hr78* and *Hr46* have previously been described as affecting pigmentation (Zelhof *et al.* 1995; Lam *et al.* 1999). *HR38* directly regulates expression of *Ddc* (Davis *et al.* 2007), another gene required for pigment synthesis (Wright 1987), and affects *yellow*-dependent melanization caused by the immune response (Sekine *et al.* 2011). Finally, one of the other TFs showing an effect on pigmentation when knocked down by RNAi, *Ventral veins lacking* (*Vvl*), regulates expression of ecdysone biosynthetic enzymes (Danielsen *et al.* 2014), providing another potential link between ecdysone signaling and pigmentation.

### Conclusions

The goal of this study was to identify potential direct regulators of the pigmentation gene *yellow* by using complementary biochemical and genetic approaches. We tested *yellow* enhancer fragments from three *Drosophila* species, *D. melanogaster*, *D. pseudoobscura*, and *D. willistoni*, against 670 TFs using a biochemical assay (Y1H), which identified 45 potential regulators of *yellow*. We also tested 124 of these 670 TFs, plus another TF known to be genetically upstream of *yellow* in the nervous system with RNAi to determine the effects of reducing their activity on adult abdominal pigmentation. We identified 32 TFs that altered adult abdominal pigmentation in *D. melanogaster* when knocked down by RNAi, 23 of which have not previously been implemented in pigmentation development, and nine of which were also identified in the Y1H screen as potential direct regulators of *yellow*. Taken together, these data provide the largest list of putative regulators of *yellow* identified to date, while also revealing unexpected links among pigmentation, male-courtship behavior, and ecdysone signaling. This information sets the stage for studies testing possible functional relationships between these TFs and *yellow*, as well as provides additional candidates for the *trans*-regulatory factors contributing to divergent *yellow* expression among species.

## 

## Supplementary Material

Supplemental Material

## References

[bib1] Anand A. Villella A. Ryner L. C. Carlo T. Goodwin S. F., 2001 Mole-cular genetic dissection of the sex-specific and vital functions of the *Drosophila melanogaster* sex determination gene fruitless. Genetics 158: 1569–1595. 11514448 10.1093/genetics/158.4.1569 PMC1461753

[bib2] Arnoult L. Su K. F. Y. Manoel D. Minervino C. Magriña J., 2013 Emergence and diversification of fly pigmentation through evolution of a gene regulatory module. Science 339: 1423–1426. 23520110 10.1126/science.1233749

[bib3] Astle J. Kozlova T. Thummel C. S., 2003 Essential roles for the Dhr78 orphan nuclear receptor during molting of the *Drosophila* tracheal system. Insect Biochem. Mol. Biol. 33: 1201–1209. 14599492 10.1016/j.ibmb.2003.06.011

[bib4] Attrill H. Falls K. Goodman J. L. Millburn G. H. Antonazzo G., 2016 FlyBase: establishing a gene group resource for *Drosophila melanogaster*. Nucleic Acids Res. 44: D786–D792. 26467478 10.1093/nar/gkv1046 PMC4702782

[bib5] Bastock M., 1956 A gene mutation which changes a behavior pattern. Evolution 10: 421–439.

[bib6] Bratty M. A. Chintapalli V. R. Dow J. A. T. Zhang T. Watson D. G., 2012 Metabolomic profiling reveals that *Drosophila melanogaster* larvae with the y mutation have altered lysine metabolism. FEBS Open Bio 2: 217–221. 10.1016/j.fob.2012.07.007 PMC3642158 23650603

[bib7] Burnet B. Connolly K. Harrison B., 1973 Phenocopies of pigmentary and behavioral effects of the yellow mutant in *Drosophila* induced by α-dimethyltyrosine. Science 181(4104): 10591060. 10.1126/science.181.4104.1059 4199226

[bib8] Calleja M. Moreno E. Pelaz S. Morata G., 1996 Visualization of gene expression in living adult *Drosophila*. Science 274: 252–255. 8824191 10.1126/science.274.5285.252

[bib9] Calleja M. Herranz H. Estella C. Casal J. Lawrence P., 2000 Generation of medial and lateral dorsal body domains by the pannier gene of *Drosophila*. Development 127: 3971–3980. 10952895 10.1242/dev.127.18.3971

[bib10] Calleja M. Renaud O. Usui K. Pistillo D. Morata G., 2002 How to pattern an epithelium: lessons from achaete-scute regulation on the notum of *Drosophila*. Gene 292: 1–12. 12119094 10.1016/s0378-1119(02)00628-5

[bib11] Celniker S. E. Sharma S. Keelan D. J. Lewis E. B., 1990 The molecular genetics of the bithorax complex of *Drosophila*: cis-regulation in the abdominal-B domain. EMBO J. 9: 4277–4286. 2265608 10.1002/j.1460-2075.1990.tb07876.x PMC552210

[bib12] Danielsen E. T. Moeller M. E. Dorry E. Komura-Kawa T. Fujimoto Y., 2014 Transcriptional control of steroid biosynthesis genes in the *Drosophila* prothoracic gland by ventral veins lacking and knirps. PLoS Genet. 10: e1004343. 24945799 10.1371/journal.pgen.1004343 PMC4063667

[bib13] Davis M. M. Yang P. Chen L. O’Keefe S. L. Hodgetts R. B., 2007 The orphan nuclear receptor DHR38 influences transcription of the DOPA decarboxylase gene in epidermal and neural tissues of *Drosophila melanogaster*. Genome 50: 1049–1060. 18059550 10.1139/g07-084

[bib14] Deplancke B. Dupuy D. Vidal M. Walhout A. J. M., 2004 A gateway-compatible yeast one-hybrid system. Genome Res. 14: 2093–2101. 15489331 10.1101/gr.2445504 PMC528925

[bib15] Drapeau M. D. Radovic A. Wittkopp P. J. Long A. D., 2003 A gene necessary for normal male courtship, yellow, acts downstream of fruitless in the *Drosophila melanogaster* larval brain. J. Neurobiol. 55: 53–72. 12605459 10.1002/neu.10196

[bib16] Drapeau M. D. Cyran S. A. Viering M. M. Geyer P. K. Long A. D., 2006 A cis-regulatory sequence within the yellow locus of *Drosophila melanogaster* required for normal male mating success. Genetics 172: 1009–1030. 16272418 10.1534/genetics.105.045666 PMC1456202

[bib17] Fink G. R., 1964 Gene-enzyme relations in histidine biosynthesis in yeast. Science 146: 525–527. 14190241 10.1126/science.146.3643.525

[bib18] Futahashi R. Fujiwara H., 2007 Regulation of 20-hydroxyecdysone on the larval pigmentation and the expression of melanin synthesis enzymes and yellow gene of the swallowtail butterfly, *Papilio xuthus*. Insect Biochem. Mol. Biol. 37: 855–864. 17628284 10.1016/j.ibmb.2007.02.014

[bib19] Geyer P. K. Corces V. G., 1987 Separate regulatory elements are responsible for the complex pattern of tissue-specific and developmental transcription of the yellow locus in *Drosophila melanogaster*. Genes Dev. 1: 996–1004. 3123324 10.1101/gad.1.9.996

[bib20] Gietz, R. D., and R. A. Woods, 2006 Yeast transformation by the LiAc/SS Carrier DNA/PEG method. Methods Mol. Biol. 313: 107–120. 10.1385/1-59259-958-3:107 16118429

[bib21] Gompel N. Prud’homme B. Wittkopp P. J. Kassner V. A. Carroll S. B., 2005 Chance caught on the wing: cis-regulatory evolution and the origin of pigment patterns in *Drosophila*. Nature 433: 481–487. 15690032 10.1038/nature03235

[bib22] Hens K. Feuz J.-D. Isakova A. Iagovitina A. Massouras A., 2011 Automated protein-DNA interaction screening of *Drosophila* regulatory elements. Nat. Methods 8: 1065–1070. 22037703 10.1038/nmeth.1763 PMC3929264

[bib23] Hopmann R. Duncan D. Duncan I., 1995 Transvection in the iab-5,6,7 region of the bithorax complex of *Drosophila*: homology independent interactions in trans. Genetics 139: 815–833. 7713434 10.1093/genetics/139.2.815 PMC1206383

[bib24] Jeong S. Rokas A. Carroll S. B., 2006 Regulation of body pigmentation by the Abdominal-B Hox protein and its gain and loss in *Drosophila* evolution. Cell 125: 1387–1399. 16814723 10.1016/j.cell.2006.04.043

[bib25] Kalay G. Wittkopp P. J., 2010 Nomadic enhancers: tissue-specific cis-regulatory elements of yellow have divergent genomic positions among *Drosophila* species. PLoS Genet. 6: e1001222. 21151964 10.1371/journal.pgen.1001222 PMC2996884

[bib26] King-Jones K. Thummel C. S., 2005 Nuclear receptors—a perspective from *Drosophila*. Nat. Rev. Genet. 6: 311–323. 15803199 10.1038/nrg1581

[bib27] Kopp A. Duncan I. Godt D. Carroll S. B., 2000 Genetic control and evolution of sexually dimorphic characters in *Drosophila*. Nature 408: 553–559. 11117736 10.1038/35046017

[bib28] Lam G. Hall B. L. Bender M. Thummel C. S., 1999 DHR3 is required for the prepupal–pupal transition and differentiation of adult structures during *Drosophila metamorphosis*. Dev. Biol. 212: 204–216. 10419696 10.1006/dbio.1999.9343

[bib29] MacNeil L. T. Pons C. Arda H. E. Giese G. E. Myers C. L., 2015 Transcription factor activity mapping of a tissue-specific in vivo gene regulatory network. Cell Syst. 1: 152–162. 26430702 10.1016/j.cels.2015.08.003 PMC4584425

[bib30] Martin A. Orgogozo V., 2013 The Loci of repeated evolution: a catalog of genetic hotspots of phenotypic variation. Evolution 67: 1235–1250. 23617905 10.1111/evo.12081

[bib31] Martin M. Meng Y. B. Chia W., 1989 Regulatory elements involved in the tissue-specific expression of the yellow gene of *Drosophila*. Mol. Gen. Genet. 218: 118–126. 2550760 10.1007/BF00330574

[bib32] Massey J. H. Wittkopp P. J., 2016 The genetic basis of pigmentation differences within and between *Drosophila* species. Curr. Top. Dev. Biol. 119: 27–61. 27282023 10.1016/bs.ctdb.2016.03.004 PMC5002358

[bib33] Ni J.-Q. Markstein M. Binari R. Pfeiffer B. Liu L.-P., 2008 Vector and parameters for targeted transgenic RNA interference in *Drosophila melanogaster*. Nat. Methods 5: 49–51. 18084299 10.1038/nmeth1146 PMC2290002

[bib34] Ni J.-Q. Liu L.-P. Binari R. Hardy R. Shim H.-S., 2009 A *Drosophila* resource of transgenic RNAi lines for neurogenetics. Genetics 182: 1089–1100. 19487563 10.1534/genetics.109.103630 PMC2728850

[bib35] Ordway A. J. Hancuch K. N. Johnson W. Wiliams T. M. Rebeiz M., 2014 The expansion of body coloration involves coordinated evolution in cis and trans within the pigmentation regulatory network of *Drosophila prostipennis*. Dev. Biol. 392: 431–440. 24907418 10.1016/j.ydbio.2014.05.023

[bib36] Ouwerkerk P. B. F. Meijer A. H., 2011 Yeast one-hybrid screens for detection of transcription factor DNA interactions. Methods Mol. Biol. 678: 211–227. 20931383 10.1007/978-1-60761-682-5_16

[bib37] Prud’homme B. Gompel N. Rokas A. Kassner V. A. Williams T. M., 2006 Repeated morphological evolution through cis-regulatory changes in a pleiotropic gene. Nature 440: 1050–1053. 16625197 10.1038/nature04597

[bib38] Radovic A. Wittkopp P. J. Long A. D. Drapeau M. D., 2002 Immunoh-istochemical colocalization of Yellow and male-specific Fruitless in *Drosophila melanogaster* neuroblasts. Biochem. Biophys. Res. Commun. 293: 1262–1264. 12054512 10.1016/S0006-291X(02)00366-2

[bib39] Reece-Hoyes J. S. Walhout A. J. M., 2012 Gene-centered yeast one-hybrid assays. Methods Mol. Biol. 812: 189–208. 22218861 10.1007/978-1-61779-455-1_11 PMC3775493

[bib40] Riddiford L. M. Cherbas P. Truman J. W., 2000 Ecdysone receptors and their biological actions. Vitam. Horm. 60: 1–73. 11037621 10.1016/s0083-6729(00)60016-x

[bib41] Rogers W. A. Grover S. Stringer S. J. Parks J. Rebeiz M., 2013 A survey of the trans-regulatory landscape for *Drosophila melanogaster* abdominal pigmentation. Dev. Biol. 385(2): 417–432. 10.1016/j.ydbio.2013.11.013 24269556

[bib42] Russo C. A. Takezaki N. Nei M., 1995 Molecular phylogeny and divergence times of drosophilid species. Mol. Biol. Evol. 12: 391–404. 7739381 10.1093/oxfordjournals.molbev.a040214

[bib43] Sekine Y. Takagahara S. Hatanaka R. Watanabe T. Oguchi H., 2011 p38 MAPKs regulate the expression of genes in the dopamine synthesis pathway through phosphorylation of NR4A nuclear receptors. J. Cell Sci. 124: 3006–3016. 21878507 10.1242/jcs.085902

[bib44] Simicevic J. Deplancke B., 2010 DNA-centered approaches to characterize regulatory protein–DNA interaction complexes. Mol. Biosyst. 6: 462–468. 20174675 10.1039/b916137f

[bib45] Spitz F. Furlong E. E. M., 2012 Transcription factors: from enhancer binding to developmental control. Nat. Rev. Genet. 13: 613–626. 22868264 10.1038/nrg3207

[bib46] Stern D. L. Orgogozo V., 2008 The loci of evolution: how predictable is genetic evolution? Evolution 62: 2155–2177. 18616572 10.1111/j.1558-5646.2008.00450.x PMC2613234

[bib47] Tauber E. Eberl D. F., 2001 Song production in auditory mutants of *Drosophila*: the role of sensory feedback. J. Comp. Physiol. A Neuroethol. Sens. Neural Behav. Physiol. 187: 341–348. 10.1007/s003590100206 11529478

[bib48] Thummel C. S., 1995 From embryogenesis to metamorphosis: the regulation and function of *Drosophila* nuclear receptor superfamily members. Cell 83: 871–877. 8521511 10.1016/0092-8674(95)90203-1

[bib49] Wagih O. Parts L., 2014 gitter: a robust and accurate method for quantification of colony sizes from plate images. G3 (Bethesda) 4: 547–552. 24474170 10.1534/g3.113.009431 PMC3962492

[bib50] Walhout A. J. M., 2011 What does biologically meaningful mean? A perspective on gene regulatory network validation. Genome Biol. 12: 109. 21489330 10.1186/gb-2011-12-4-109 PMC3218850

[bib51] Walter M. F. Black B. C. Afshar G. Kermabon A. Y. Wright T. R., 1991 Temporal and spatial expression of the yellow gene in correlation with cuticle formation and dopa decarboxylase activity in *Drosophila* development. Dev. Biol. 147: 32–45. 1879614 10.1016/s0012-1606(05)80005-3

[bib52] Werner T. Koshikawa S. Williams T. M. Carroll S. B., 2010 Gener-ation of a novel wing colour pattern by the Wingless morphogen. Nature 464: 1143–1148. 20376004 10.1038/nature08896

[bib53] Williams T. M. Selegue J. E. Werner T. Gompel N. Kopp A., 2008 The regulation and evolution of a genetic switch controlling sexually dimorphic traits in *Drosophila*. Cell 134: 610–623. 18724934 10.1016/j.cell.2008.06.052 PMC2597198

[bib54] Wittkopp P. J. Beldade P., 2009 Development and evolution of insect pigmentation: genetic mechanisms and the potential consequences of pleiotropy. Semin. Cell Dev. Biol. 20: 65–71. 18977308 10.1016/j.semcdb.2008.10.002

[bib55] Wittkopp P. J. Kalay G., 2012 Cis-regulatory elements: molecular mechanisms and evolutionary processes underlying divergence. Nat. Rev. Genet. 13: 59–69. 10.1038/nrg3095 22143240

[bib56] Wittkopp P. J. True J. R. Carroll S. B., 2002 a Reciprocal functions of the *Drosophila* yellow and ebony proteins in the development and evolution of pigment patterns. Development 129: 1849–1858. 11934851 10.1242/dev.129.8.1849

[bib57] Wittkopp P. J. Vaccaro K. Carroll S. B., 2002 b Evolution of yellow gene regulation and pigmentation in *Drosophila*. Curr. Biol. 12: 1547–1556. 12372246 10.1016/s0960-9822(02)01113-2

[bib58] Wittkopp P. J. Carroll S. B. Kopp A., 2003 Evolution in black and white: genetic control of pigment patterns in *Drosophila*. Trends Genet. 19: 495–504. 12957543 10.1016/S0168-9525(03)00194-X

[bib59] Wright T. R., 1987 The genetics of biogenic amine metabolism, sclerotization, and melanization in *Drosophila melanogaster*. Adv. Genet. 24: 127–222. 3124532

[bib60] Zelhof A. C. Yao T. P. Evans R. M. McKeown M., 1995 Identification and characterization of a *Drosophila* nuclear receptor with the ability to inhibit the ecdysone response. Proc. Natl. Acad. Sci. USA 92: 10477–10481. 7479823 10.1073/pnas.92.23.10477 PMC40634

