# Supplementary material for: Potential Direct Regulators of the Drosophila yellow Gene Identified by Yeast One-Hybrid and RNAi Screens
Source: G3 (Bethesda). 2016 Aug 12;6(10):3419–30. doi: 10.1534/g3.116.032607 (PMC5068961; doi:10.1534/g3.116.032607)
Supplement: Supplemental Material [file supp_6_10_3419__index.html]

Potential Direct Regulators of the Drosophila yellow Gene Identified by Yeast One-Hybrid and RNAi Screens — Supplemental Material 

# Potential Direct Regulators of the *Drosophila yellow* Gene Identified by Yeast One-Hybrid and RNAi Screens

## Supplemental Material for Kalay *et al.*, 2016

**Files in this Data Supplement:**

- Table S1 - Names, sequences and annotations of primers used to amplify *yellow* enhancer subfragments tested in yeast-one-hybrid. (.xlsx, 47 KB)
- Table S2 - List of interactions between *D.melanogaster* transcription factors and *yellow* enhancer subfragments identified using yeast-one-hybrid and screened based on significance threshold and the number of 3AT levels for which a given interaction was observed. (.xlsx, 182 KB)
- Table S3 - List of RNAi lines tested to look for change in adult abdominal pigmentation. (.xlsx, 78 KB)
- Table S4 - List of transcription factors that affected abdominal pigmentation as a result of RNAi knockdown as well as detailed descriptions of the resulting phenotypes. (.xlsx, 63 KB)
- File S1 - DNA sequences of *yellow* enhancer subfragments tested with yeast-one-hybrid in fasta format. (.txt, 27 KB)
- File S2 - Zipped raw image files of test and control plates used for each *yellow* enhancer subfragment in yeast-one-hybrid. (.zip, 98 MB)
- File S3 - Raw yeast-one-hybrid data produced by Gitter analysis. (.zip, 1 MB)
- File S4 - R scripts used to create Figure 5 and Table S2 using File S3 as data source. (.txt, 19 KB)
- File S5 - Cuticle images of control and test flies where RNAi was used to knockdown a transcription factor gene. (.zip, 7 MB)
