# Supplementary figures and images for: Potential Direct Regulators of the Drosophila yellow Gene Identified by Yeast One-Hybrid and RNAi Screens (part 2 of 3)
Source: G3 (Bethesda). 2016 Aug 12;6(10):3419–30. doi: 10.1534/g3.116.032607 (PMC5068961; doi:10.1534/g3.116.032607)

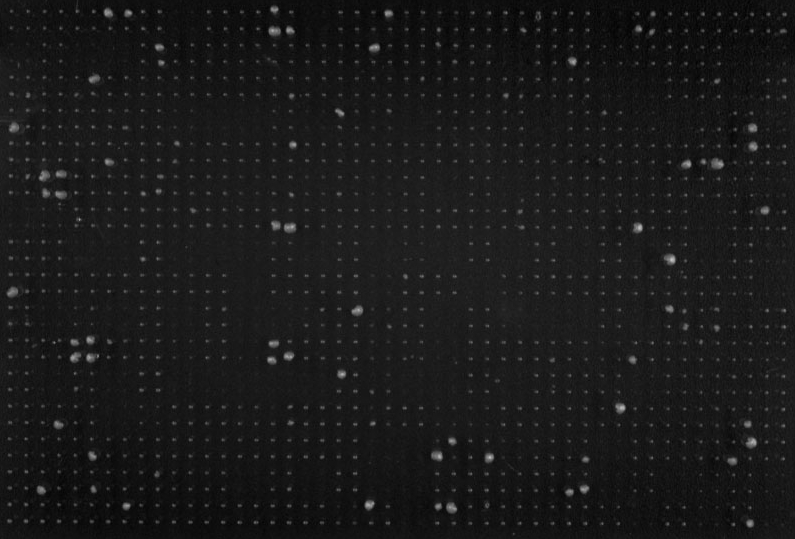

Supplement: Supplemental Material [file supp_g3.116.032607_FileS2.zip › IndividualImagesForSupplementalFile2/mel_A7-TF2-10mM3AT-after7days.1sc.png]

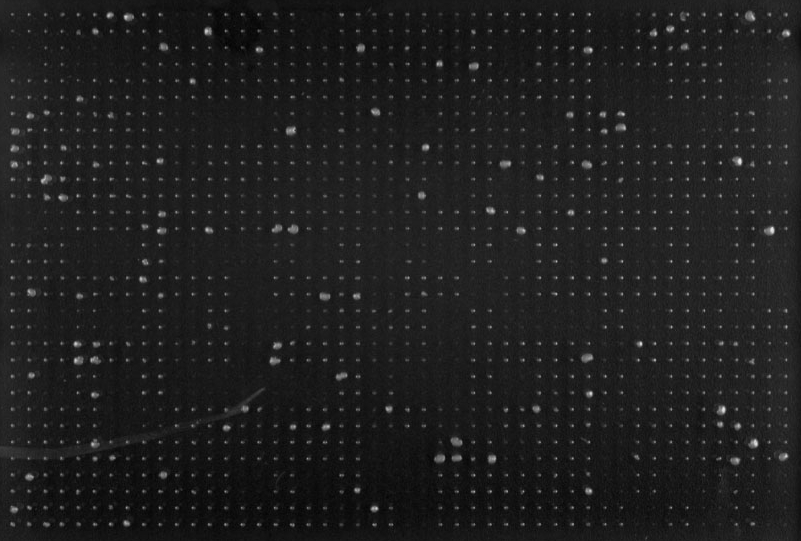

Supplement: Supplemental Material [file supp_g3.116.032607_FileS2.zip › IndividualImagesForSupplementalFile2/mel_A7-TF2-20mM3AT-after10days.png]

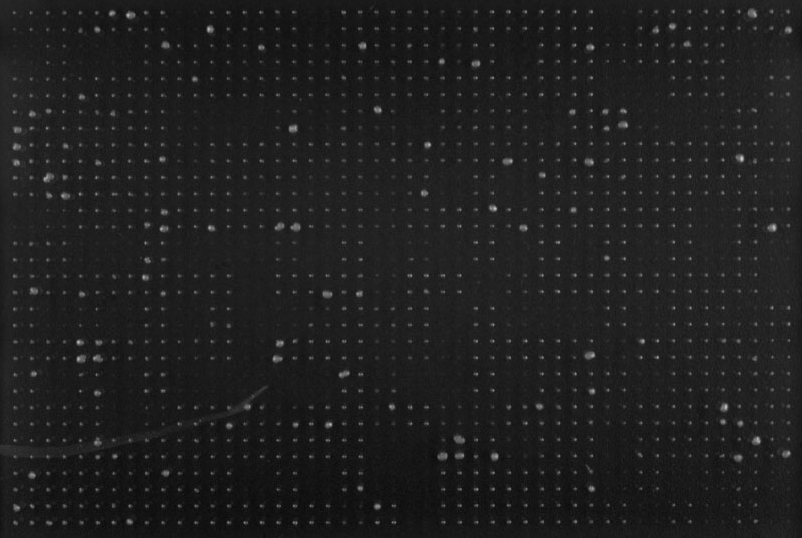

Supplement: Supplemental Material [file supp_g3.116.032607_FileS2.zip › IndividualImagesForSupplementalFile2/mel_A7-TF2-20mM3AT-after7days.1sc.png]

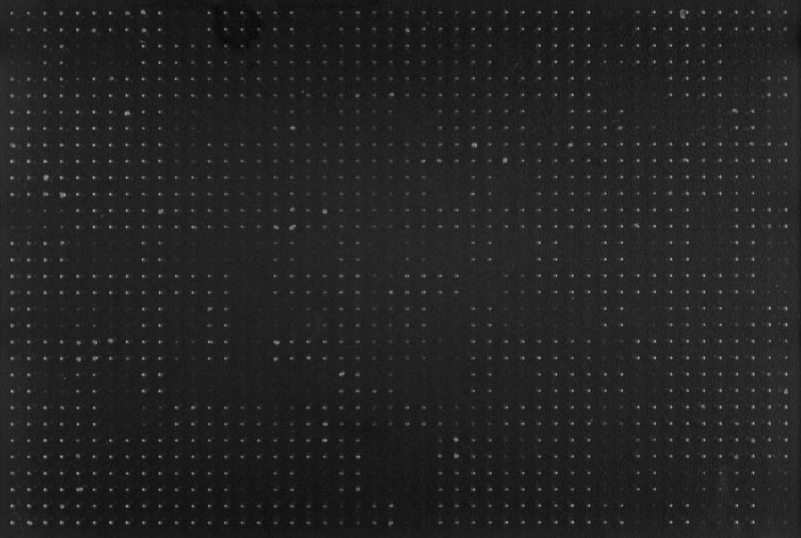

Supplement: Supplemental Material [file supp_g3.116.032607_FileS2.zip › IndividualImagesForSupplementalFile2/mel_A7-TF2-40mM3AT-after10days.png]

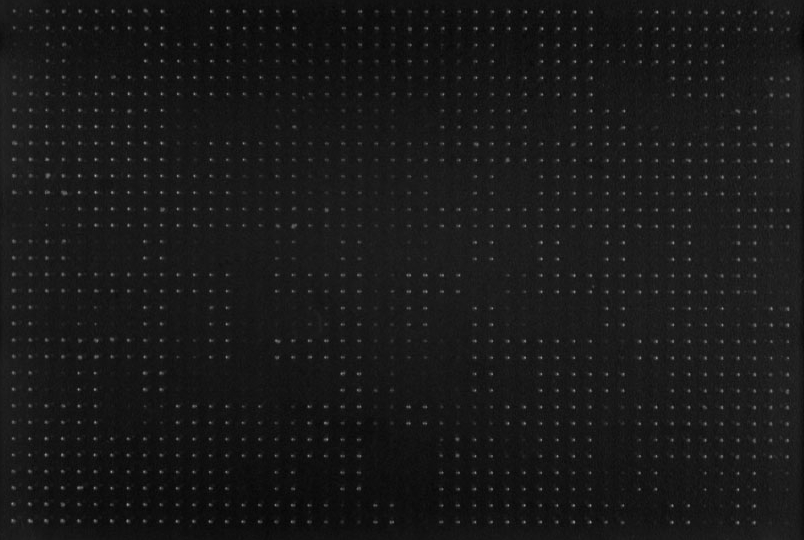

Supplement: Supplemental Material [file supp_g3.116.032607_FileS2.zip › IndividualImagesForSupplementalFile2/mel_A7-TF2-40mM3AT-after7days.1sc.png]

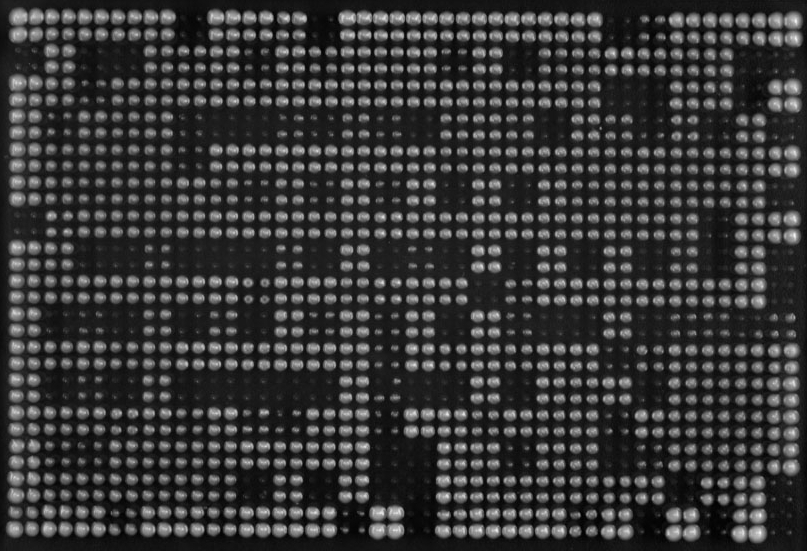

Supplement: Supplemental Material [file supp_g3.116.032607_FileS2.zip › IndividualImagesForSupplementalFile2/mel_A7-TF2-no3AT-3days-1536.1sc.png]

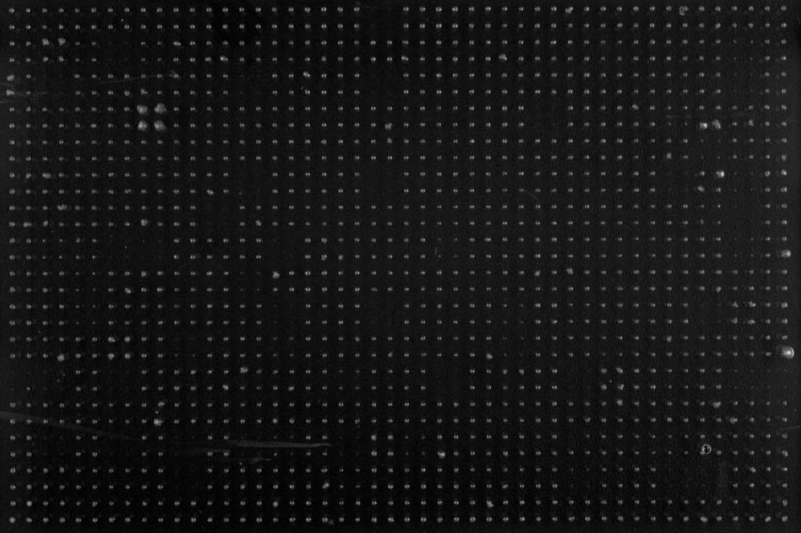

Supplement: Supplemental Material [file supp_g3.116.032607_FileS2.zip › IndividualImagesForSupplementalFile2/mel_A8-TF1-10mM3AT-after10days.png]

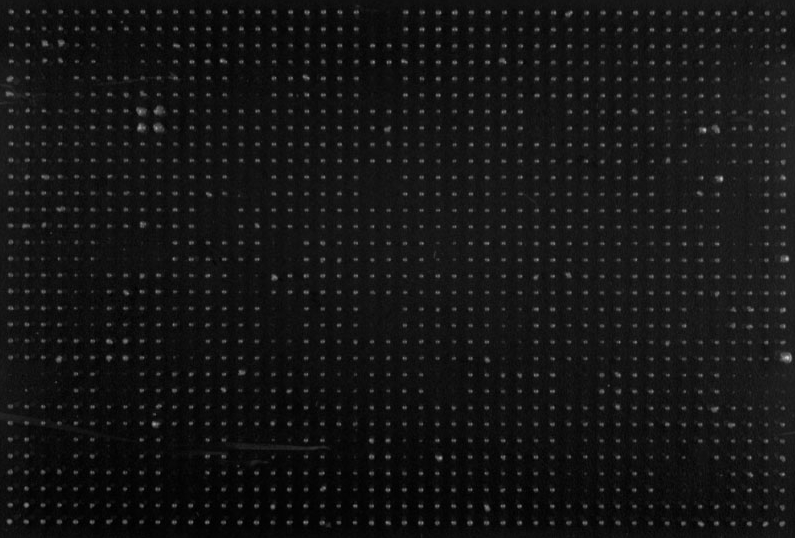

Supplement: Supplemental Material [file supp_g3.116.032607_FileS2.zip › IndividualImagesForSupplementalFile2/mel_A8-TF1-10mM3AT-after7days.1sc.png]

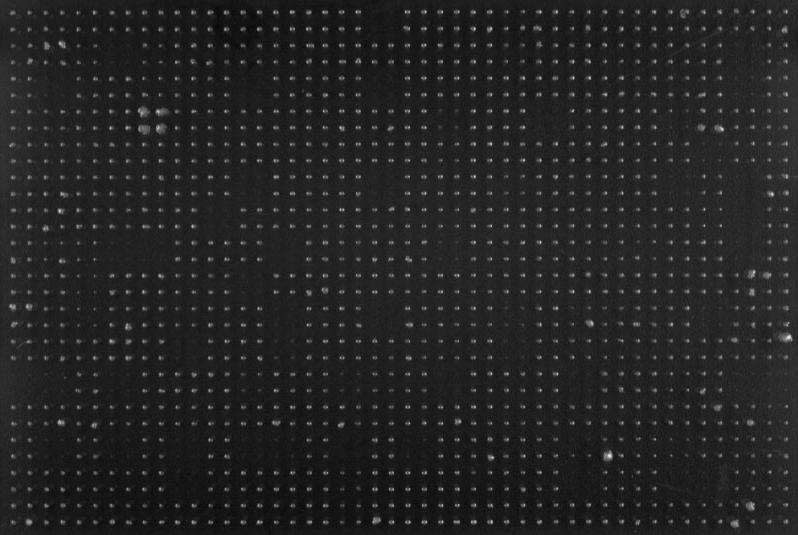

Supplement: Supplemental Material [file supp_g3.116.032607_FileS2.zip › IndividualImagesForSupplementalFile2/mel_A8-TF1-20mM3AT-after10days.png]

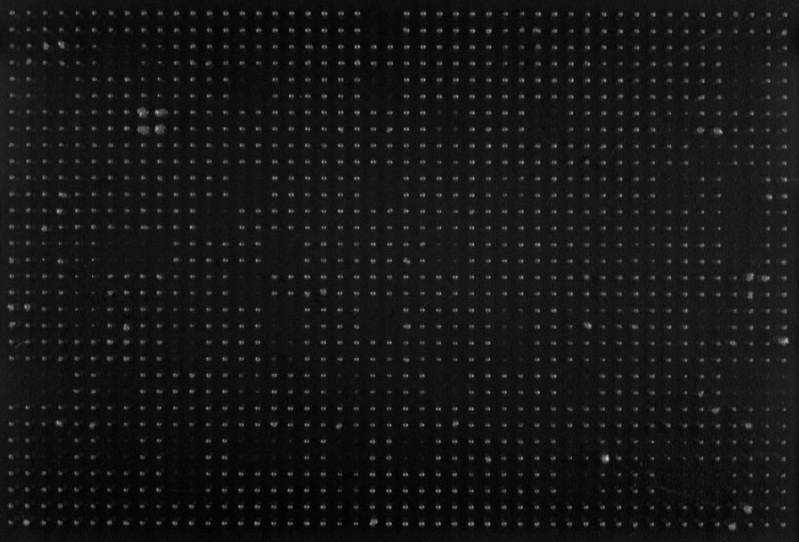

Supplement: Supplemental Material [file supp_g3.116.032607_FileS2.zip › IndividualImagesForSupplementalFile2/mel_A8-TF1-20mM3AT-after7days.1sc.png]

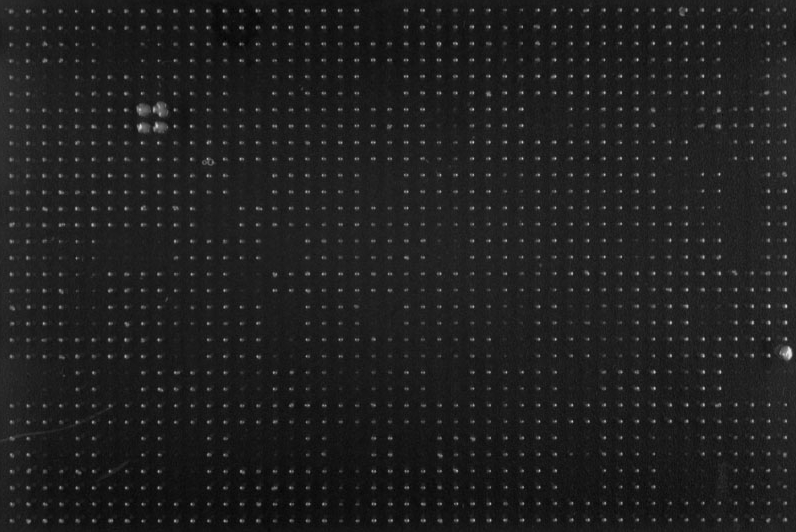

Supplement: Supplemental Material [file supp_g3.116.032607_FileS2.zip › IndividualImagesForSupplementalFile2/mel_A8-TF1-40mM3AT-after10days.png]

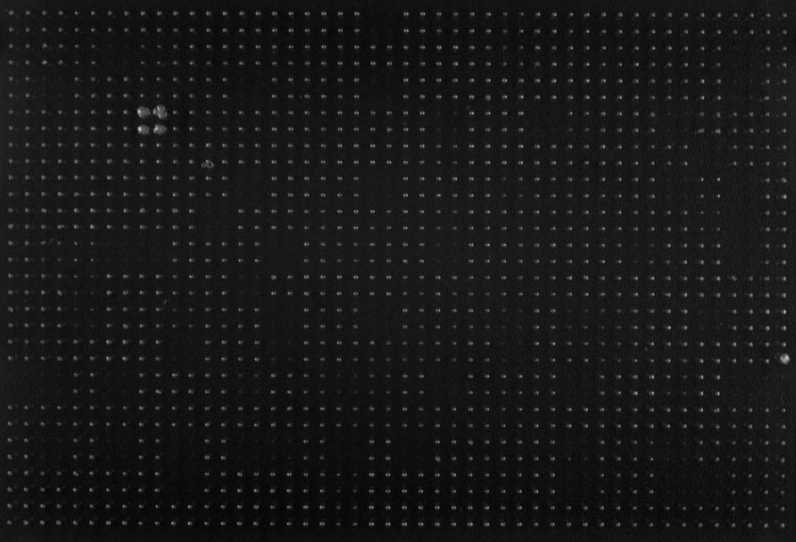

Supplement: Supplemental Material [file supp_g3.116.032607_FileS2.zip › IndividualImagesForSupplementalFile2/mel_A8-TF1-40mM3AT-after7days.1sc.png]

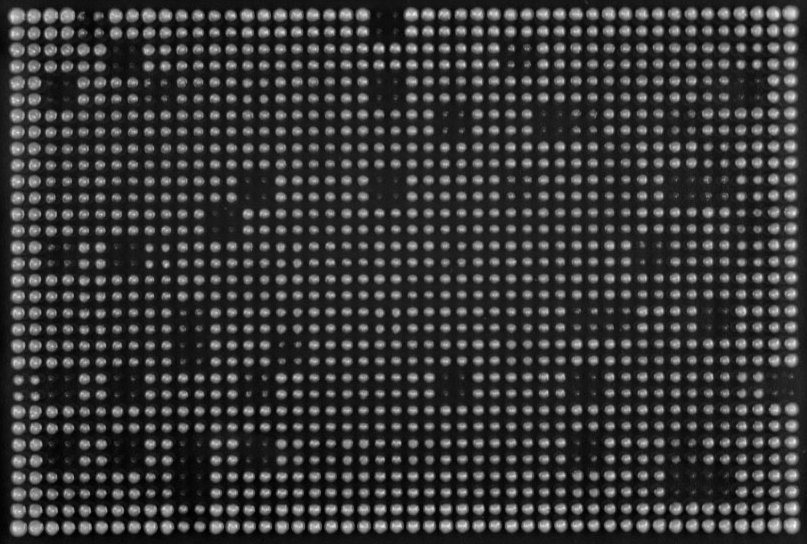

Supplement: Supplemental Material [file supp_g3.116.032607_FileS2.zip › IndividualImagesForSupplementalFile2/mel_A8-TF1-no3AT-3days-1536.1sc.png]

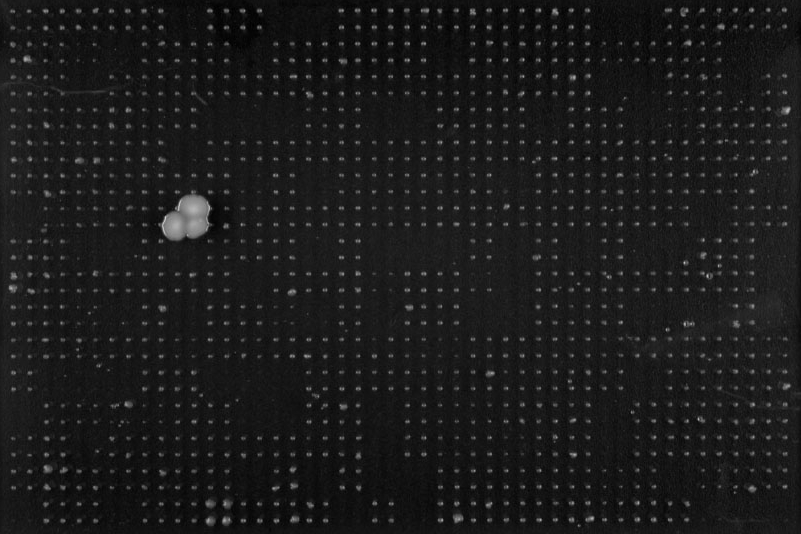

Supplement: Supplemental Material [file supp_g3.116.032607_FileS2.zip › IndividualImagesForSupplementalFile2/mel_A8-TF2-10mM3AT-after10days.png]

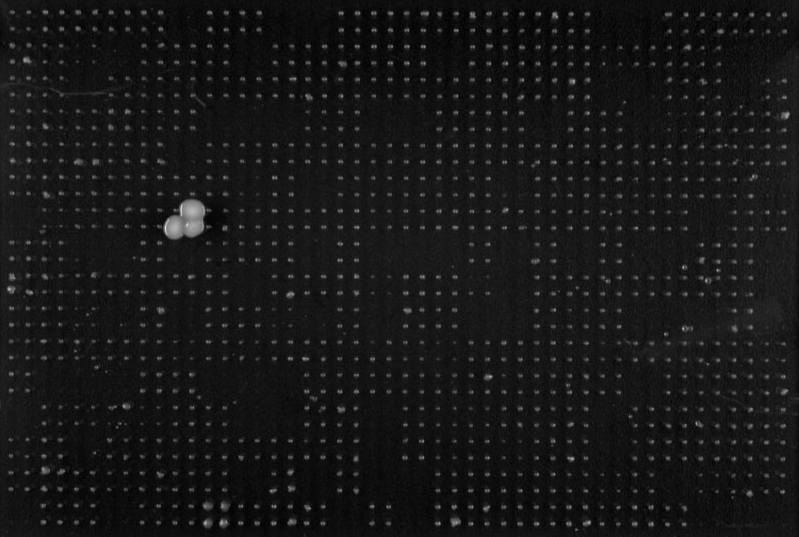

Supplement: Supplemental Material [file supp_g3.116.032607_FileS2.zip › IndividualImagesForSupplementalFile2/mel_A8-TF2-10mM3AT-after7days.1sc.png]

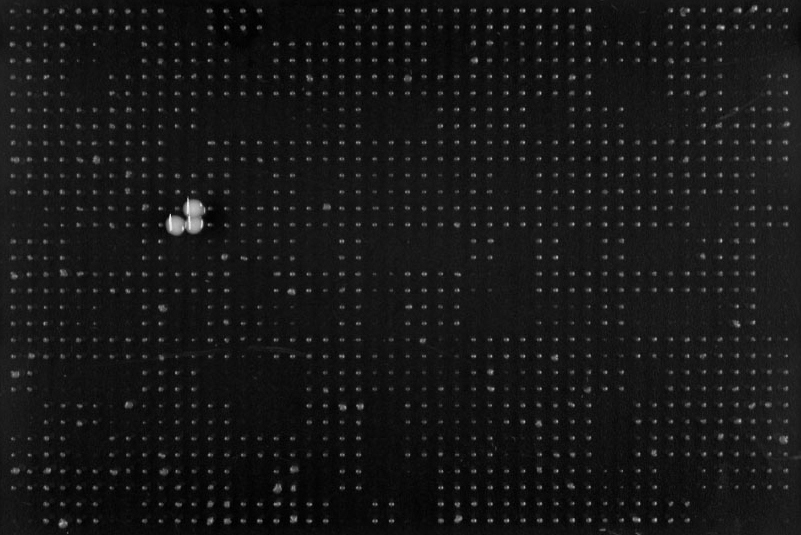

Supplement: Supplemental Material [file supp_g3.116.032607_FileS2.zip › IndividualImagesForSupplementalFile2/mel_A8-TF2-20mM3AT-after10days.png]

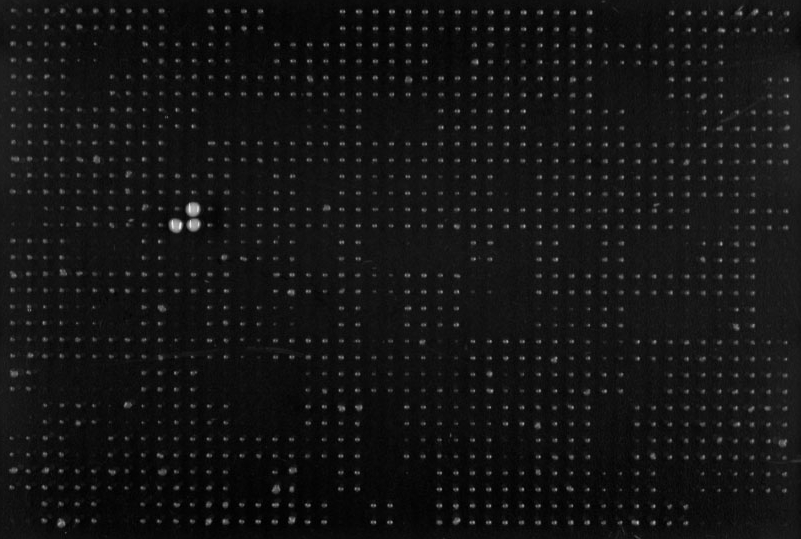

Supplement: Supplemental Material [file supp_g3.116.032607_FileS2.zip › IndividualImagesForSupplementalFile2/mel_A8-TF2-20mM3AT-after7days.1sc.png]

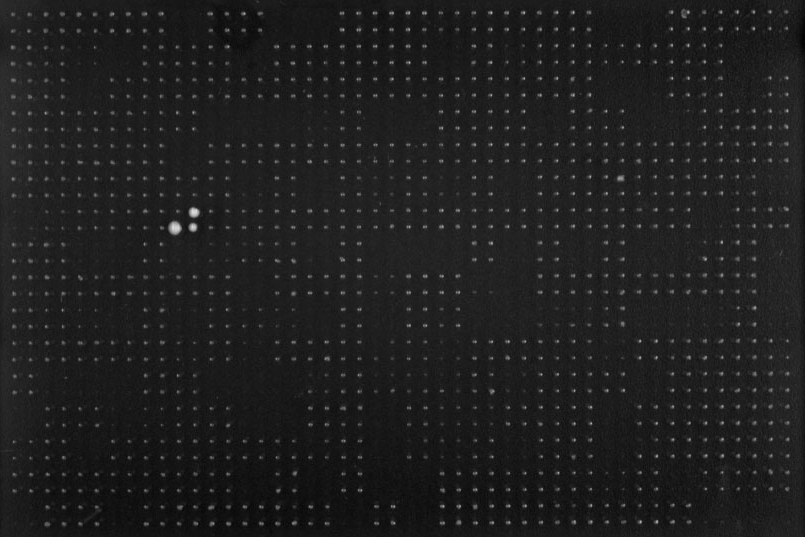

Supplement: Supplemental Material [file supp_g3.116.032607_FileS2.zip › IndividualImagesForSupplementalFile2/mel_A8-TF2-40mM3AT-after10days.png]

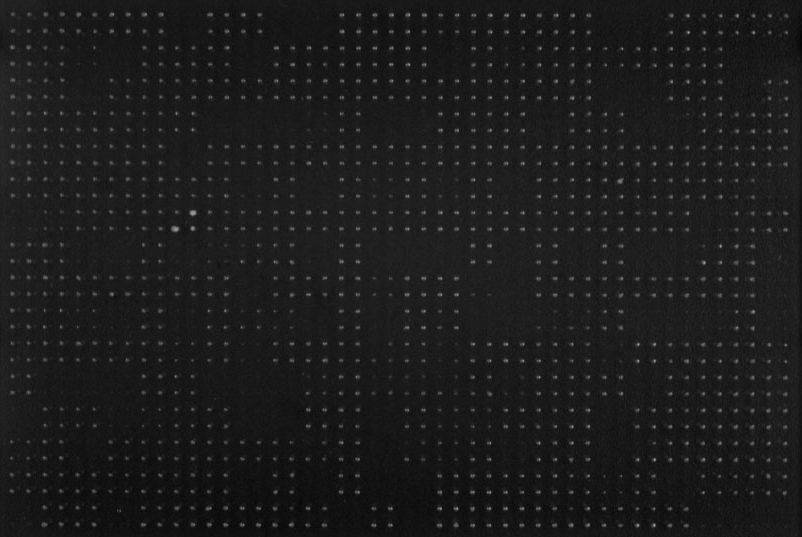

Supplement: Supplemental Material [file supp_g3.116.032607_FileS2.zip › IndividualImagesForSupplementalFile2/mel_A8-TF2-40mM3AT-after7days.1sc.png]

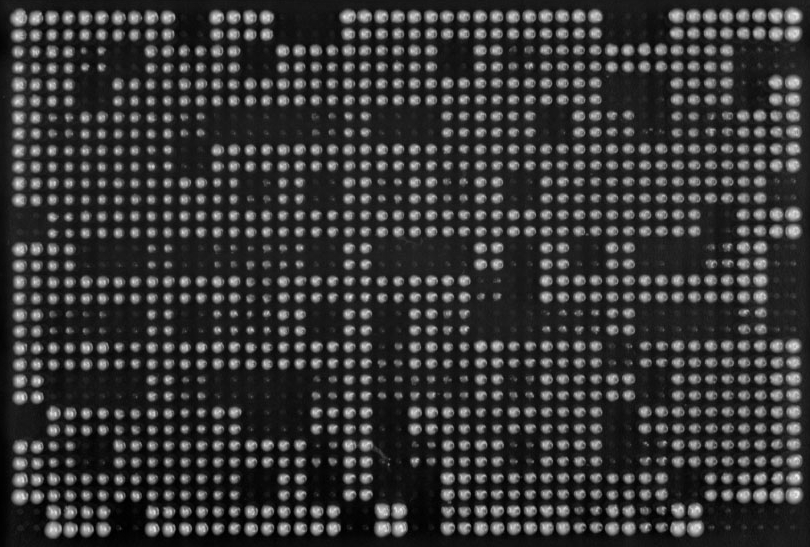

Supplement: Supplemental Material [file supp_g3.116.032607_FileS2.zip › IndividualImagesForSupplementalFile2/mel_A8-TF2-no3AT-3days-1536.1sc.png]

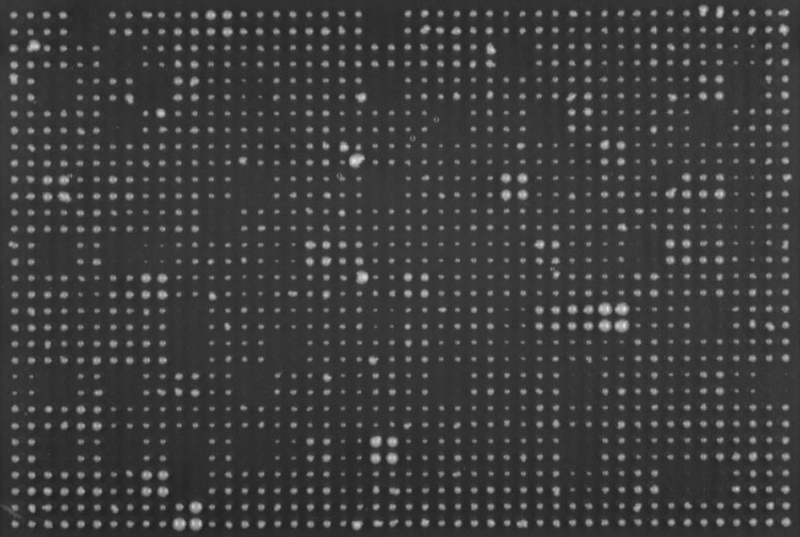

Supplement: Supplemental Material [file supp_g3.116.032607_FileS2.zip › IndividualImagesForSupplementalFile2/pse_B1-TF1-20mM3AT-after10days.png]

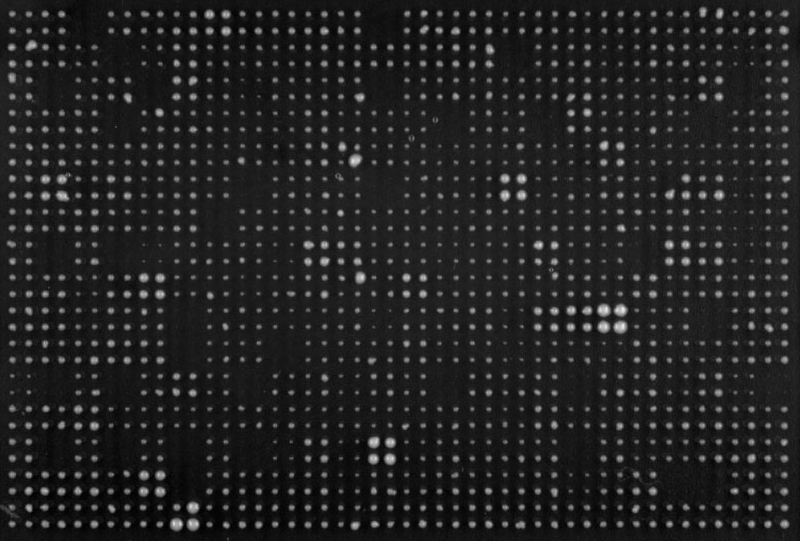

Supplement: Supplemental Material [file supp_g3.116.032607_FileS2.zip › IndividualImagesForSupplementalFile2/pse_B1-TF1-20mM3AT-after7days.1sc.png]

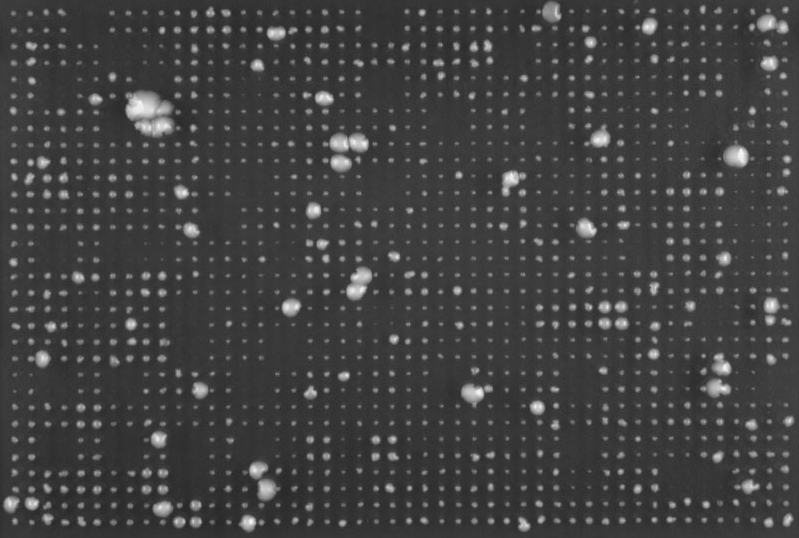

Supplement: Supplemental Material [file supp_g3.116.032607_FileS2.zip › IndividualImagesForSupplementalFile2/pse_B1-TF1-40mM3AT-after10days.png]

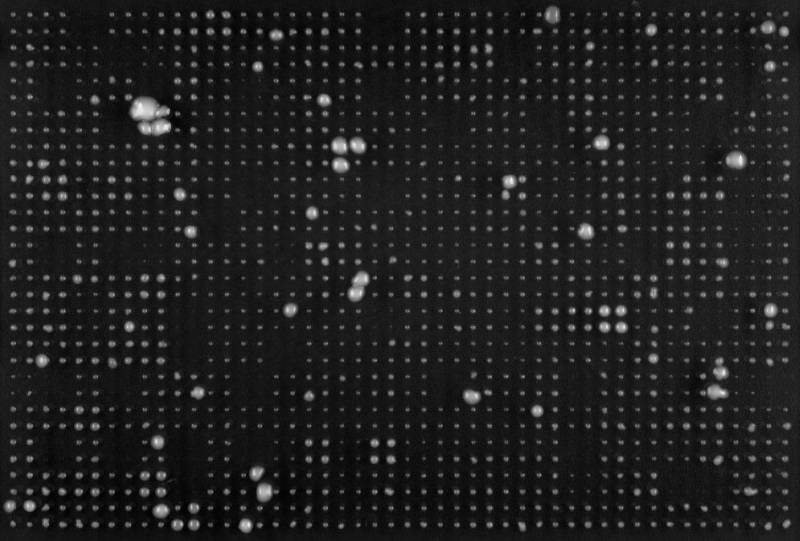

Supplement: Supplemental Material [file supp_g3.116.032607_FileS2.zip › IndividualImagesForSupplementalFile2/pse_B1-TF1-40mM3AT-after7days.1sc.png]

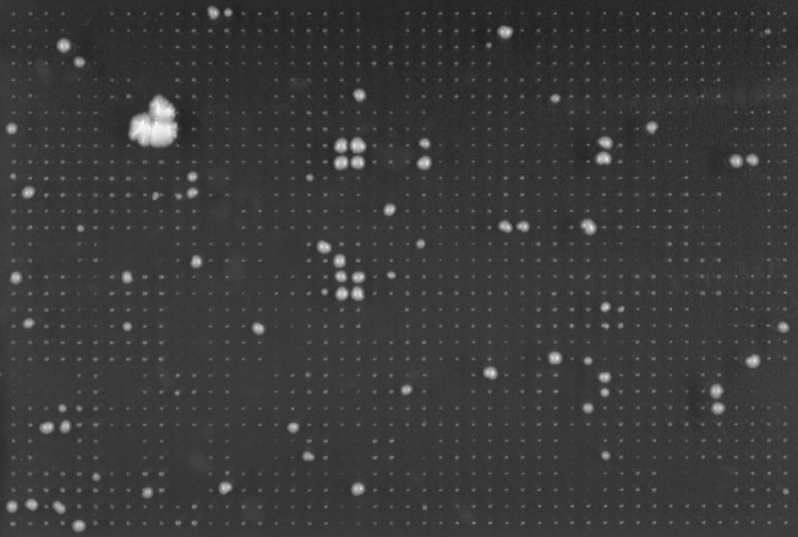

Supplement: Supplemental Material [file supp_g3.116.032607_FileS2.zip › IndividualImagesForSupplementalFile2/pse_B1-TF1-80mM3AT-after10days.png]

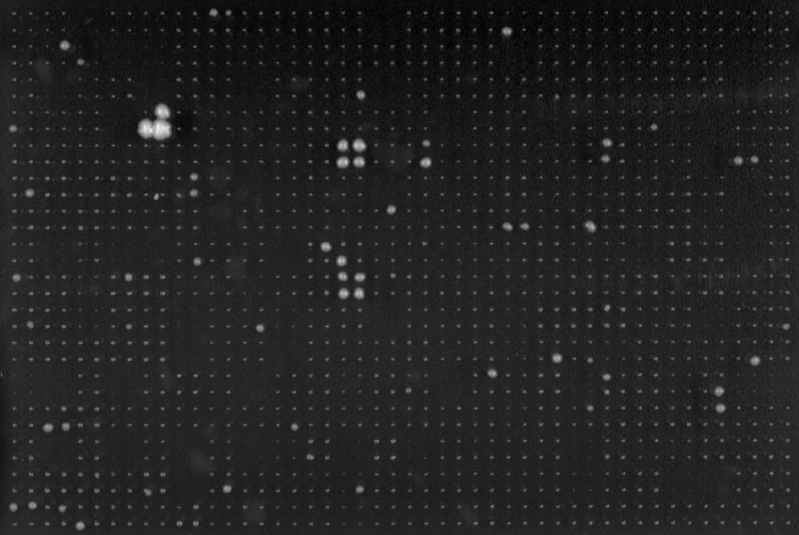

Supplement: Supplemental Material [file supp_g3.116.032607_FileS2.zip › IndividualImagesForSupplementalFile2/pse_B1-TF1-80mM3AT-after7days.1sc.png]

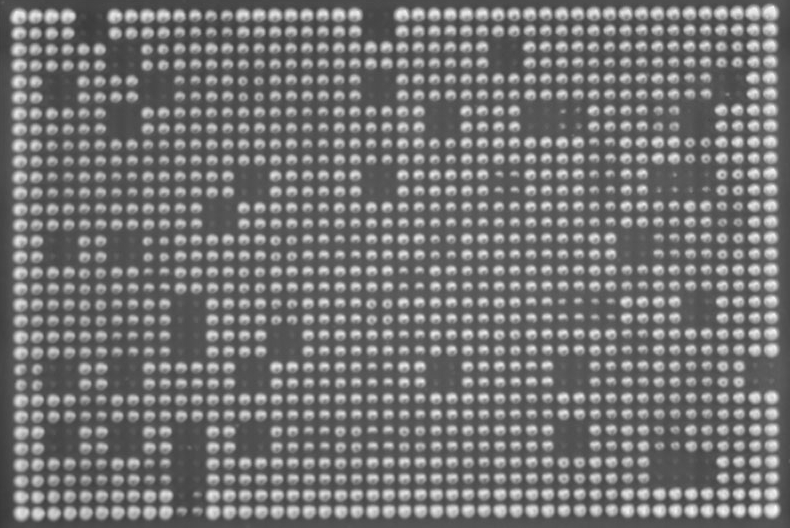

Supplement: Supplemental Material [file supp_g3.116.032607_FileS2.zip › IndividualImagesForSupplementalFile2/pse_B1-TF1-no3AT-1536-3days.1sc.png]

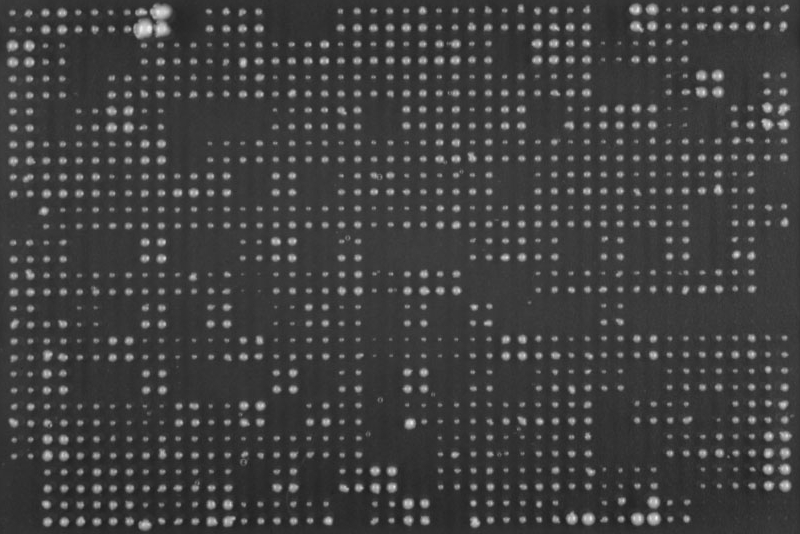

Supplement: Supplemental Material [file supp_g3.116.032607_FileS2.zip › IndividualImagesForSupplementalFile2/pse_B1-TF2-20mM3AT-after10days.png]

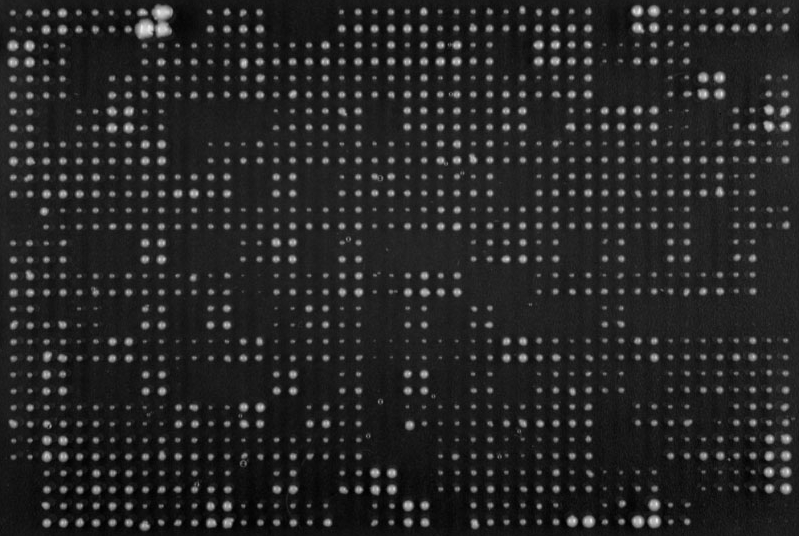

Supplement: Supplemental Material [file supp_g3.116.032607_FileS2.zip › IndividualImagesForSupplementalFile2/pse_B1-TF2-20mM3AT-after7days.1sc.png]

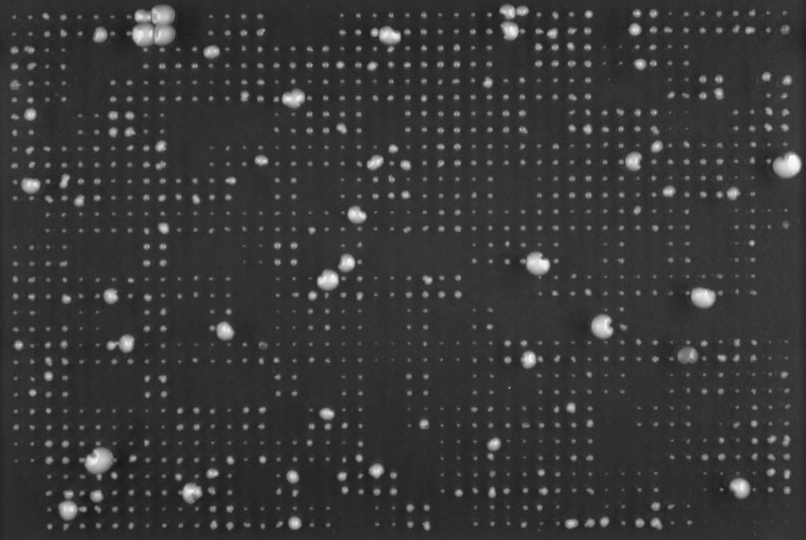

Supplement: Supplemental Material [file supp_g3.116.032607_FileS2.zip › IndividualImagesForSupplementalFile2/pse_B1-TF2-40mM3AT-after10days.png]

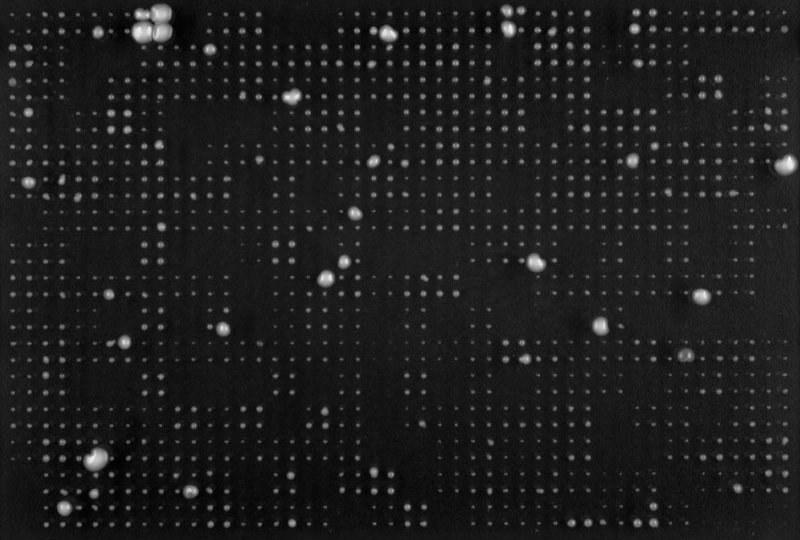

Supplement: Supplemental Material [file supp_g3.116.032607_FileS2.zip › IndividualImagesForSupplementalFile2/pse_B1-TF2-40mM3AT-after7days.1sc.png]

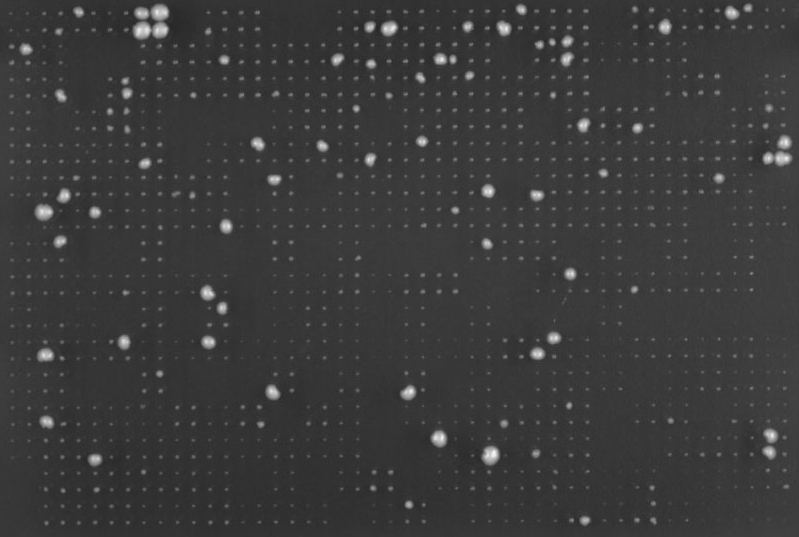

Supplement: Supplemental Material [file supp_g3.116.032607_FileS2.zip › IndividualImagesForSupplementalFile2/pse_B1-TF2-60mM3AT-after10days.png]

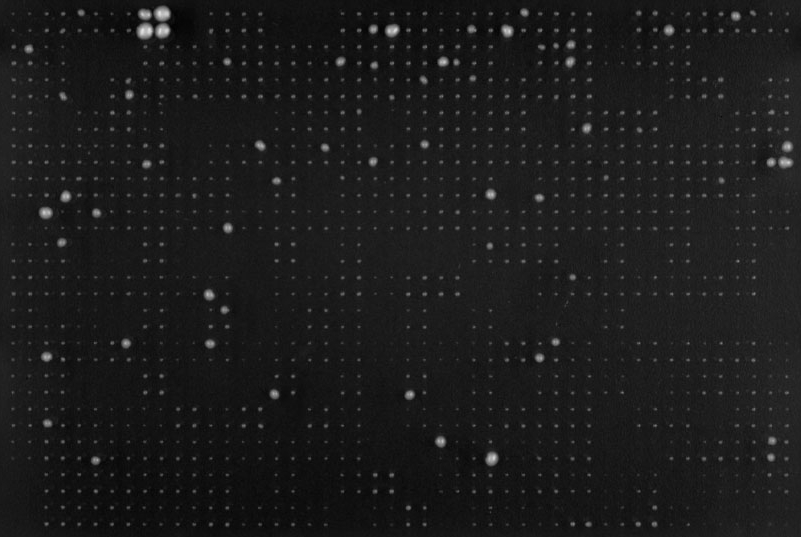

Supplement: Supplemental Material [file supp_g3.116.032607_FileS2.zip › IndividualImagesForSupplementalFile2/pse_B1-TF2-60mM3AT-after7days.1sc.png]

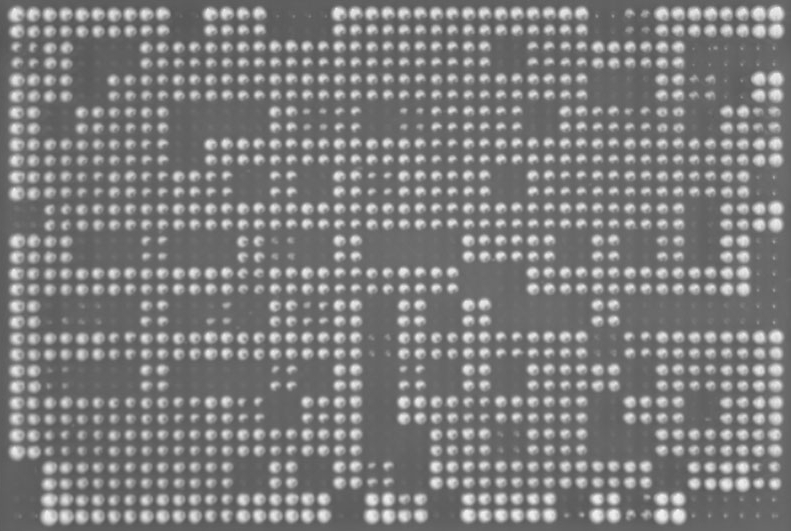

Supplement: Supplemental Material [file supp_g3.116.032607_FileS2.zip › IndividualImagesForSupplementalFile2/pse_B1-TF2-no3AT-1536-3days.1sc.png]

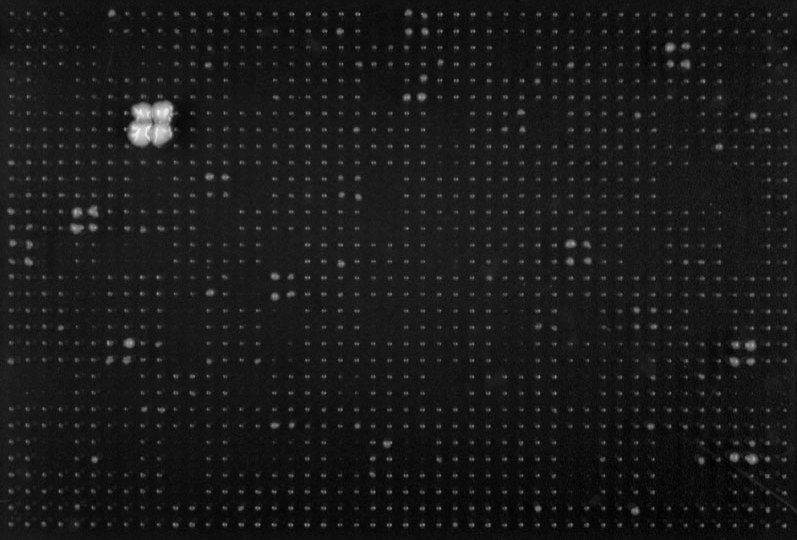

Supplement: Supplemental Material [file supp_g3.116.032607_FileS2.zip › IndividualImagesForSupplementalFile2/pse_B10-TF1-100mM3AT-after7days.png]

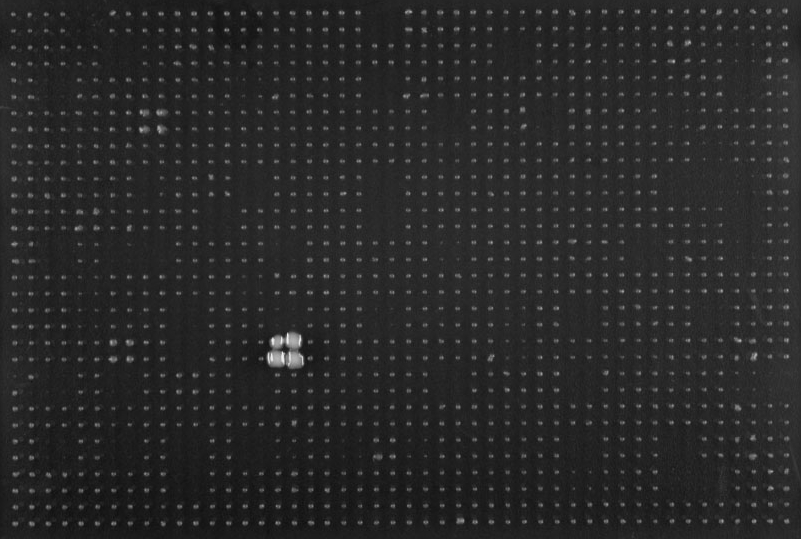

Supplement: Supplemental Material [file supp_g3.116.032607_FileS2.zip › IndividualImagesForSupplementalFile2/pse_B10-TF1-20mM3AT-after10days.png]

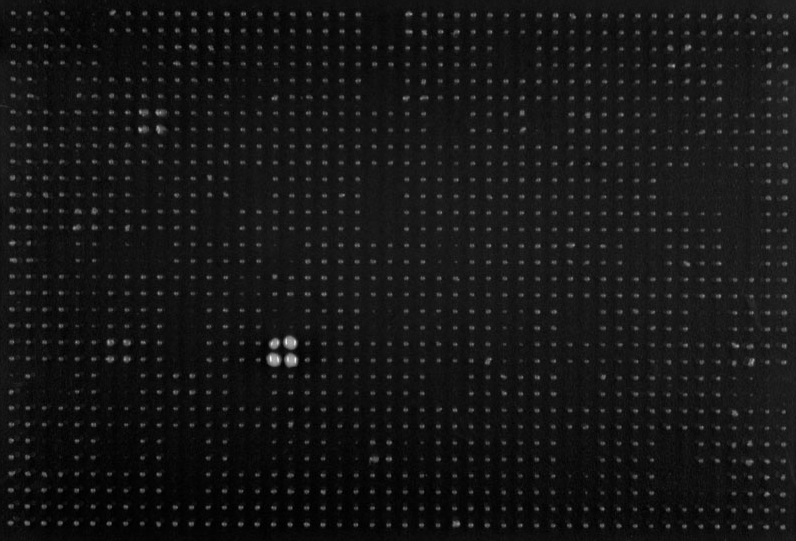

Supplement: Supplemental Material [file supp_g3.116.032607_FileS2.zip › IndividualImagesForSupplementalFile2/pse_B10-TF1-20mM3AT-after7days.1sc.png]

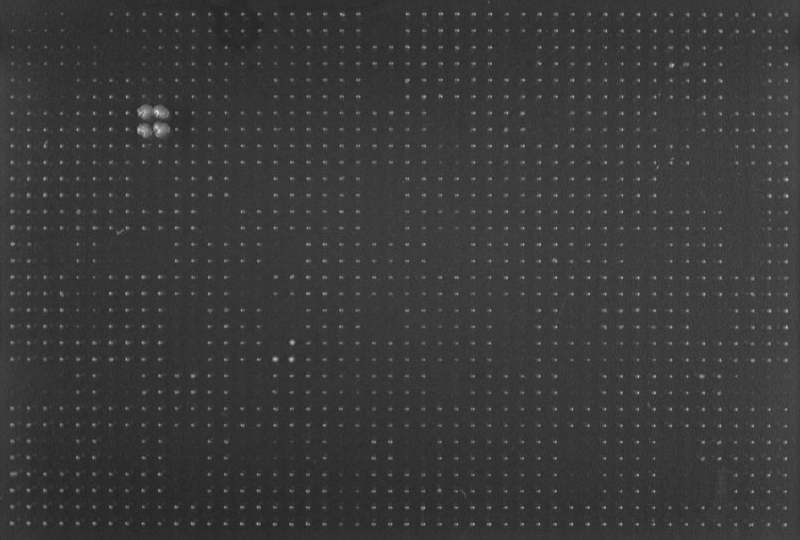

Supplement: Supplemental Material [file supp_g3.116.032607_FileS2.zip › IndividualImagesForSupplementalFile2/pse_B10-TF1-60mM3AT-after10days.png]

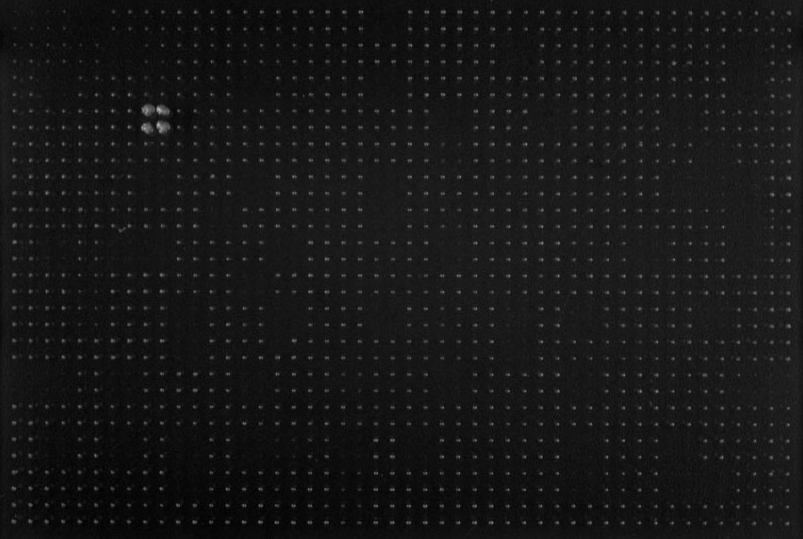

Supplement: Supplemental Material [file supp_g3.116.032607_FileS2.zip › IndividualImagesForSupplementalFile2/pse_B10-TF1-60mM3AT-after7days.1sc.png]

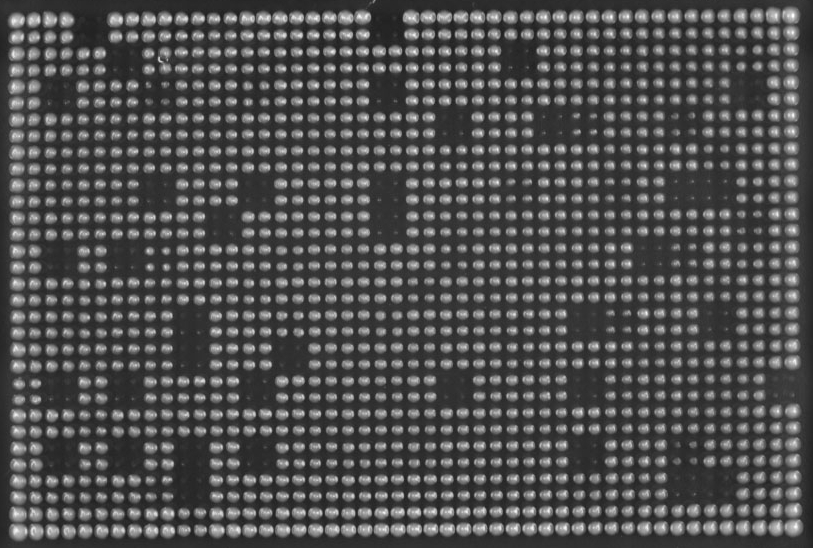

Supplement: Supplemental Material [file supp_g3.116.032607_FileS2.zip › IndividualImagesForSupplementalFile2/pse_B10-TF1-no3AT-3days-1536.1sc.png]

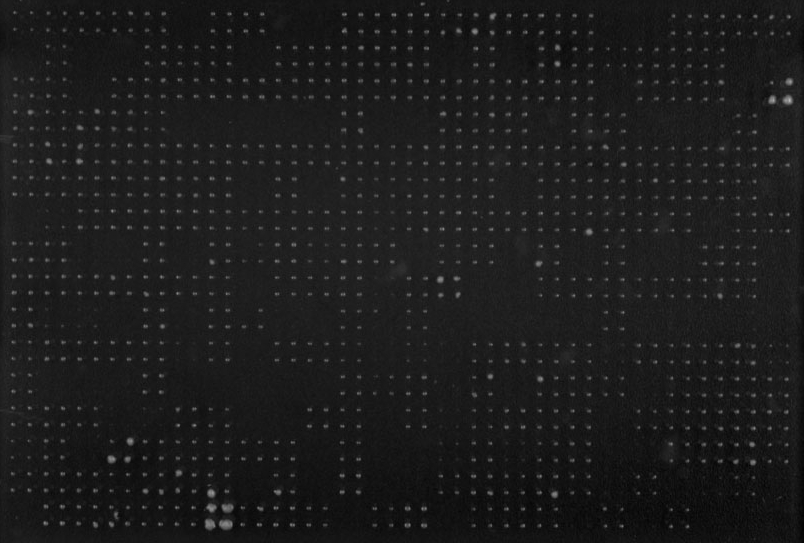

Supplement: Supplemental Material [file supp_g3.116.032607_FileS2.zip › IndividualImagesForSupplementalFile2/pse_B10-TF2-100mM3AT-after7days.png]

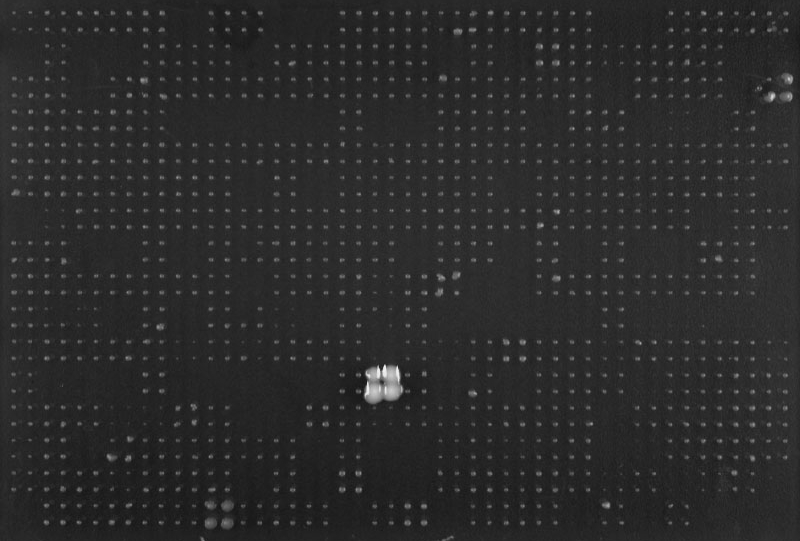

Supplement: Supplemental Material [file supp_g3.116.032607_FileS2.zip › IndividualImagesForSupplementalFile2/pse_B10-TF2-20mM3AT-after10days.png]

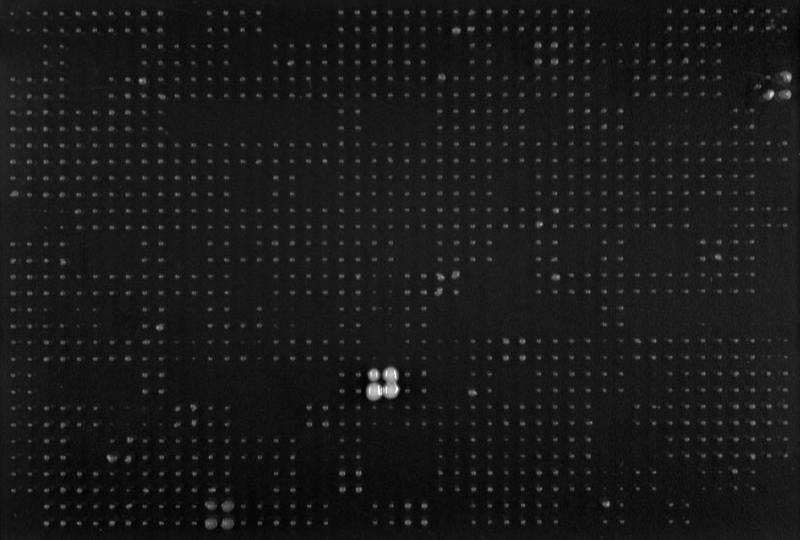

Supplement: Supplemental Material [file supp_g3.116.032607_FileS2.zip › IndividualImagesForSupplementalFile2/pse_B10-TF2-20mM3AT-after7days.1sc.png]

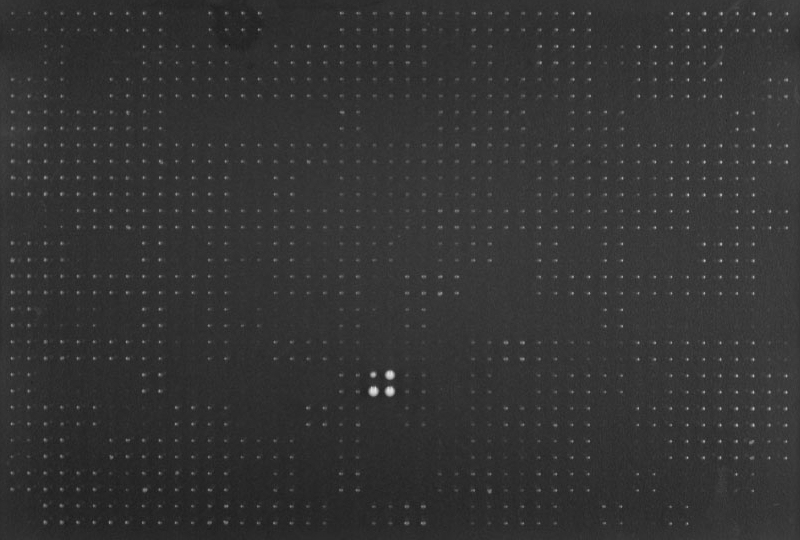

Supplement: Supplemental Material [file supp_g3.116.032607_FileS2.zip › IndividualImagesForSupplementalFile2/pse_B10-TF2-60mM3AT-after10days.png]

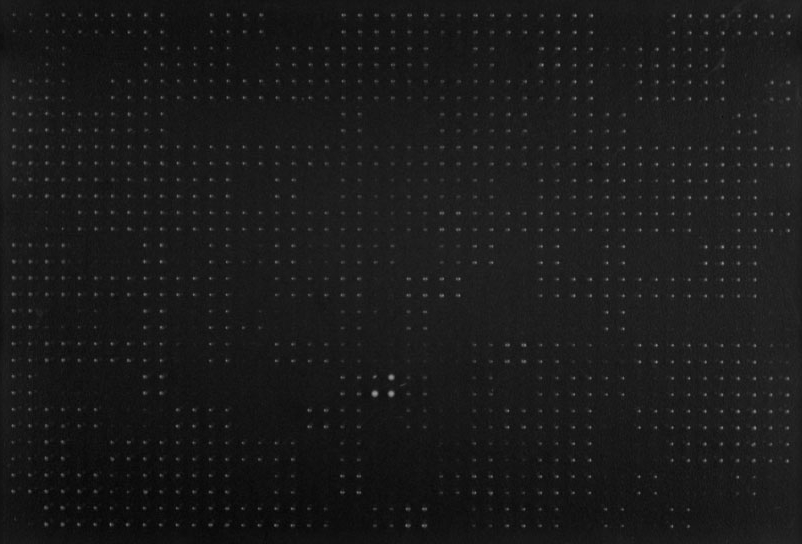

Supplement: Supplemental Material [file supp_g3.116.032607_FileS2.zip › IndividualImagesForSupplementalFile2/pse_B10-TF2-60mM3AT-after7days.1sc.png]

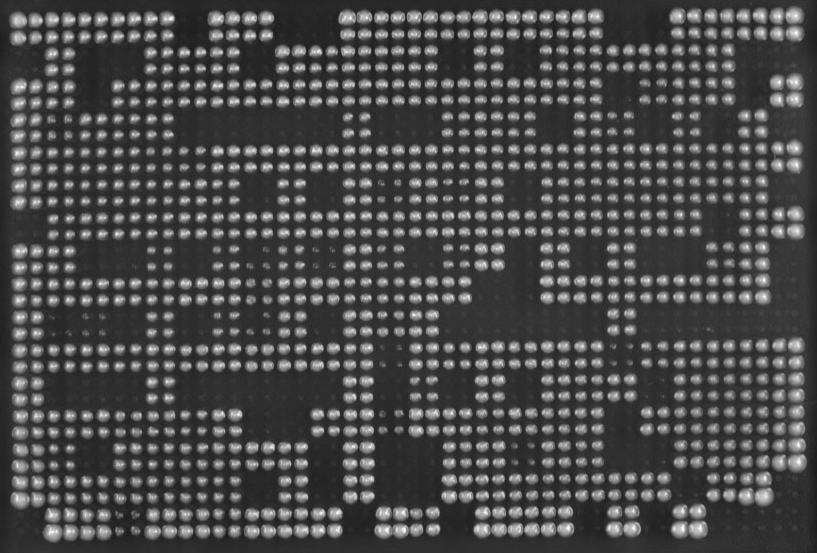

Supplement: Supplemental Material [file supp_g3.116.032607_FileS2.zip › IndividualImagesForSupplementalFile2/pse_B10-TF2-no3AT-3days-1536.1sc.png]

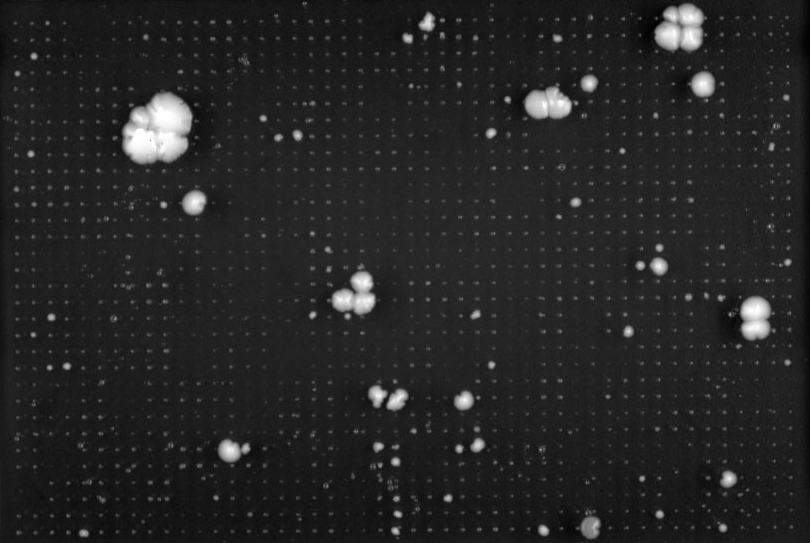

Supplement: Supplemental Material [file supp_g3.116.032607_FileS2.zip › IndividualImagesForSupplementalFile2/pse_B2-TF1-10mM3AT-after10days.png]

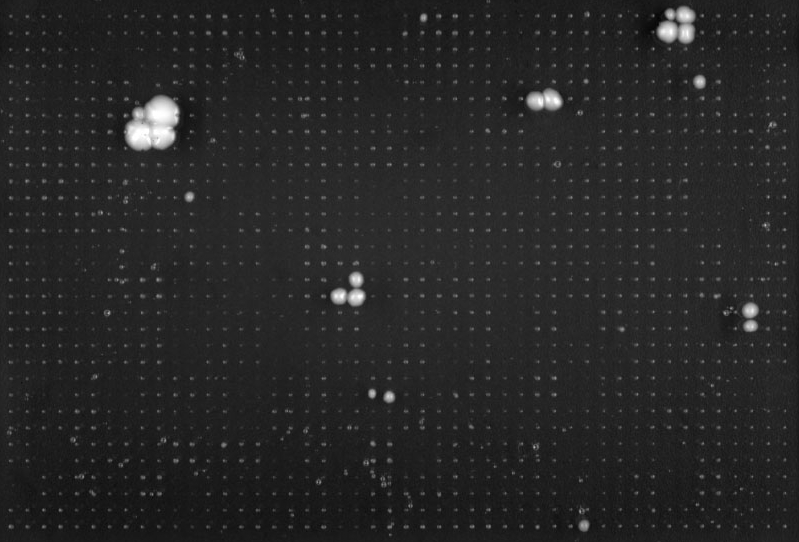

Supplement: Supplemental Material [file supp_g3.116.032607_FileS2.zip › IndividualImagesForSupplementalFile2/pse_B2-TF1-10mM3AT-after7days.1sc.png]

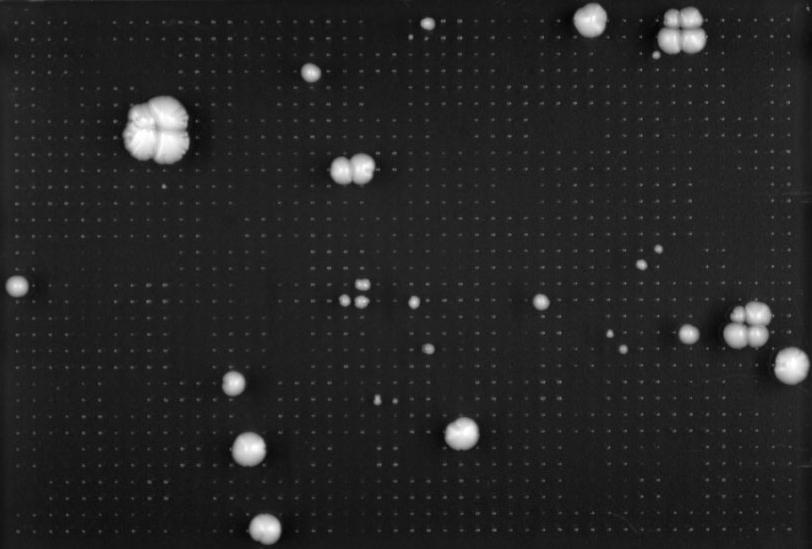

Supplement: Supplemental Material [file supp_g3.116.032607_FileS2.zip › IndividualImagesForSupplementalFile2/pse_B2-TF1-20mM3AT-after10days.png]

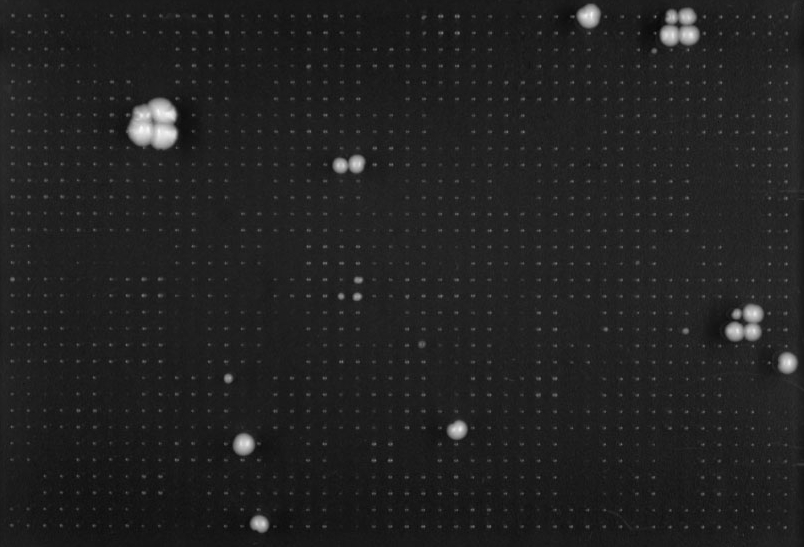

Supplement: Supplemental Material [file supp_g3.116.032607_FileS2.zip › IndividualImagesForSupplementalFile2/pse_B2-TF1-20mM3AT-after7days.1sc.png]

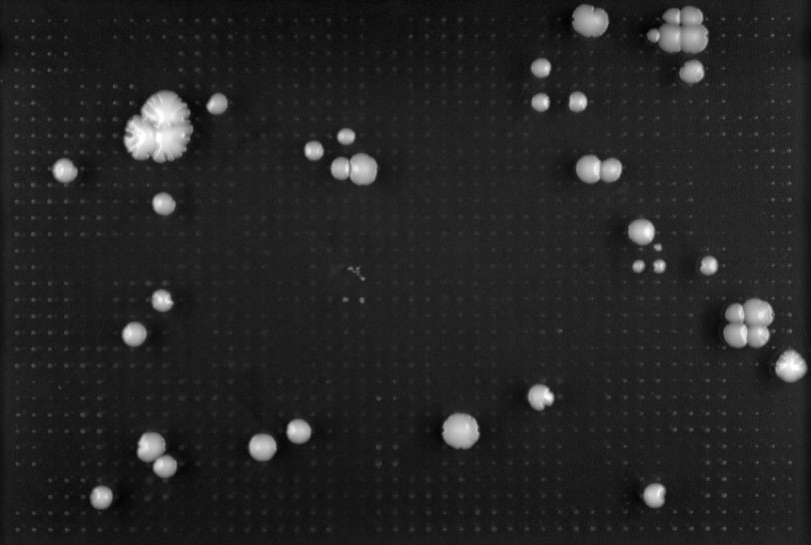

Supplement: Supplemental Material [file supp_g3.116.032607_FileS2.zip › IndividualImagesForSupplementalFile2/pse_B2-TF1-40mM3AT-after10days.png]

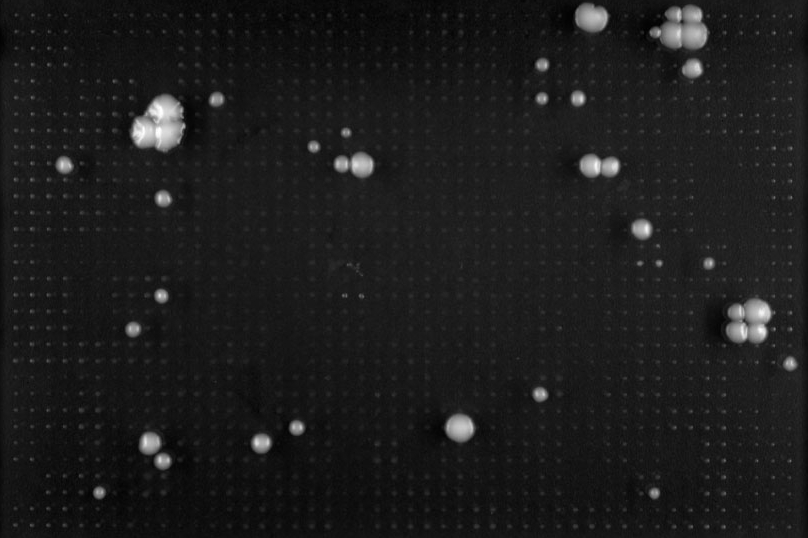

Supplement: Supplemental Material [file supp_g3.116.032607_FileS2.zip › IndividualImagesForSupplementalFile2/pse_B2-TF1-40mM3AT-after7days.1sc.png]

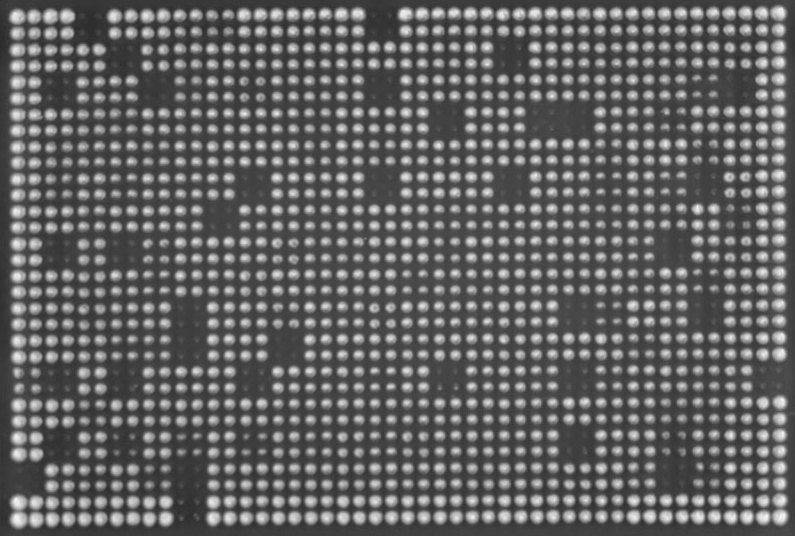

Supplement: Supplemental Material [file supp_g3.116.032607_FileS2.zip › IndividualImagesForSupplementalFile2/pse_B2-TF1-no3AT-1536-3days.1sc.png]

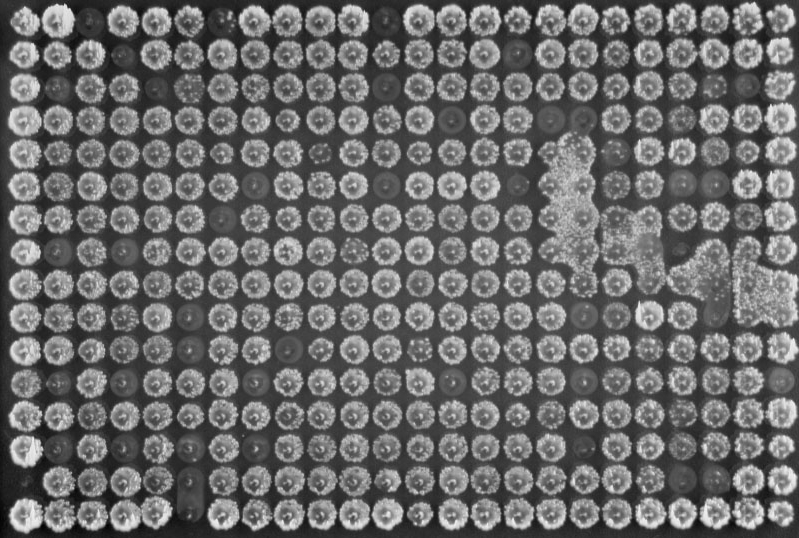

Supplement: Supplemental Material [file supp_g3.116.032607_FileS2.zip › IndividualImagesForSupplementalFile2/pse_B2-TF1-no3AT-after3days.1sc.png]

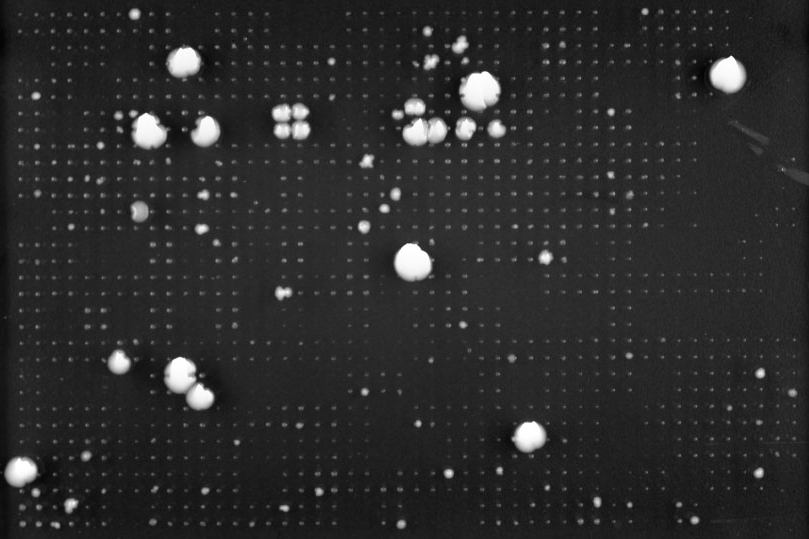

Supplement: Supplemental Material [file supp_g3.116.032607_FileS2.zip › IndividualImagesForSupplementalFile2/pse_B2-TF2-10mM3AT-after10days.png]

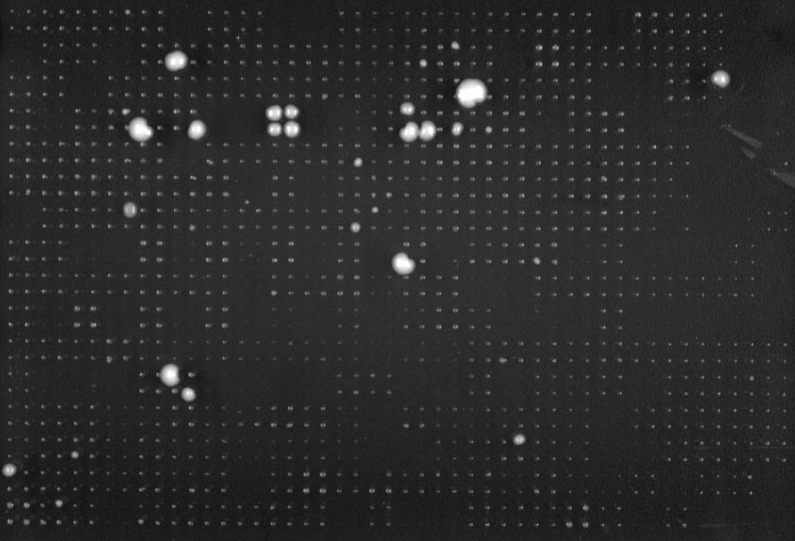

Supplement: Supplemental Material [file supp_g3.116.032607_FileS2.zip › IndividualImagesForSupplementalFile2/pse_B2-TF2-10mM3AT-after7days.1sc.png]

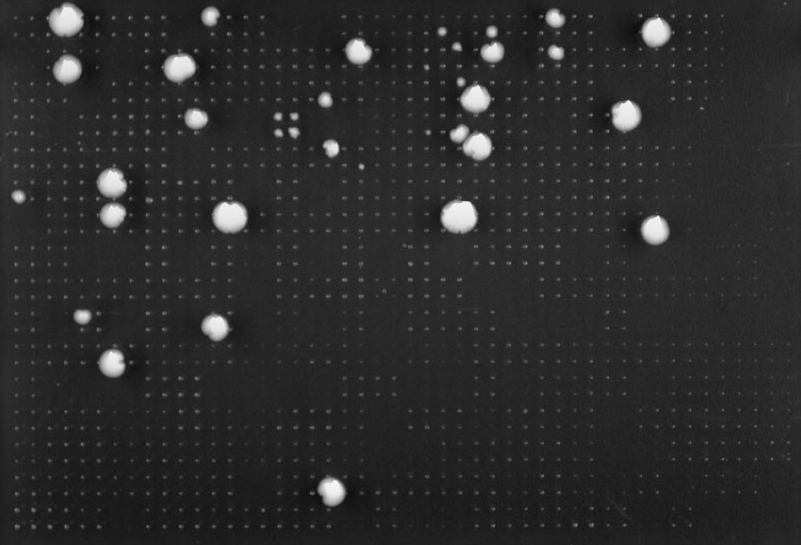

Supplement: Supplemental Material [file supp_g3.116.032607_FileS2.zip › IndividualImagesForSupplementalFile2/pse_B2-TF2-20mM3AT-after10days.png]

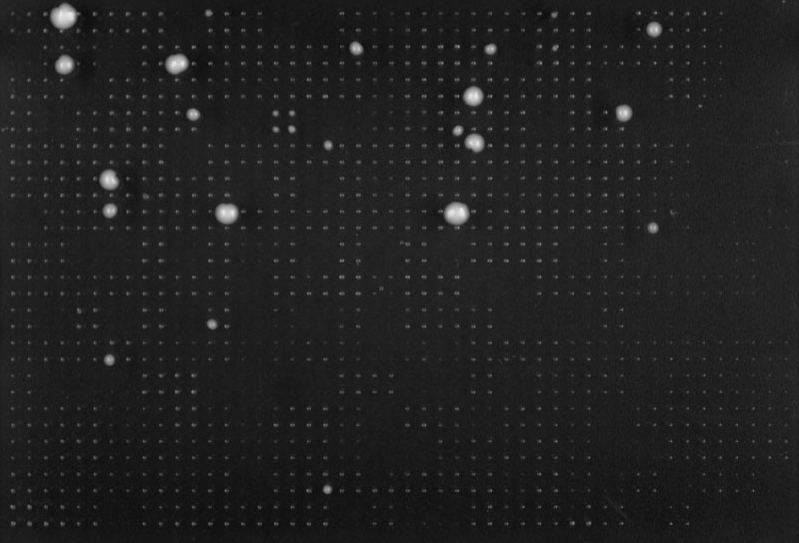

Supplement: Supplemental Material [file supp_g3.116.032607_FileS2.zip › IndividualImagesForSupplementalFile2/pse_B2-TF2-20mM3AT-after7days.1sc.png]

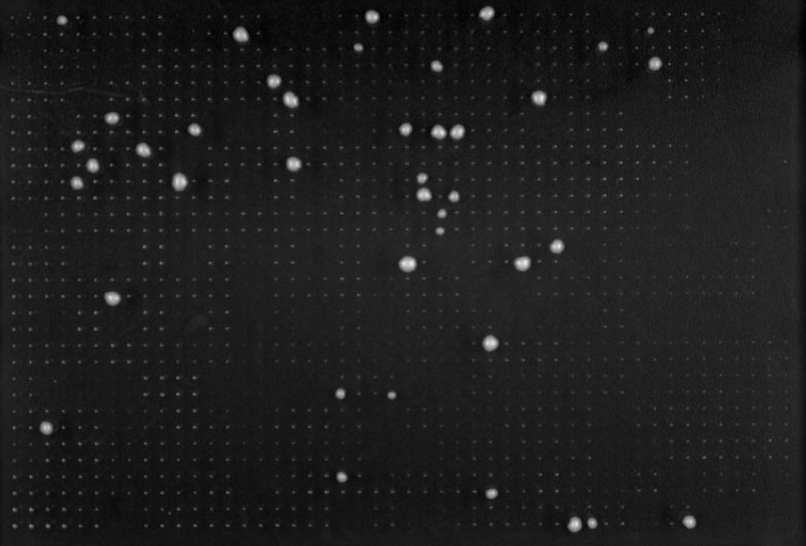

Supplement: Supplemental Material [file supp_g3.116.032607_FileS2.zip › IndividualImagesForSupplementalFile2/pse_B2-TF2-40mM3AT-after10days.png]

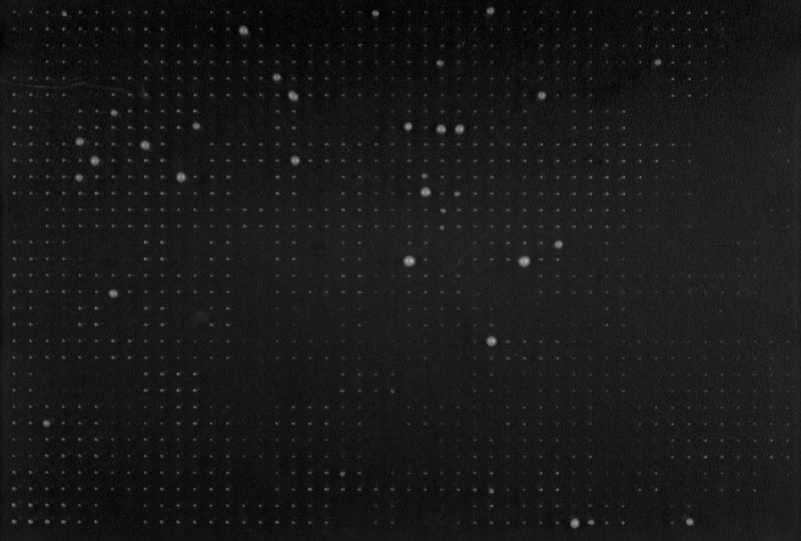

Supplement: Supplemental Material [file supp_g3.116.032607_FileS2.zip › IndividualImagesForSupplementalFile2/pse_B2-TF2-40mM3AT-after7days.1sc.png]

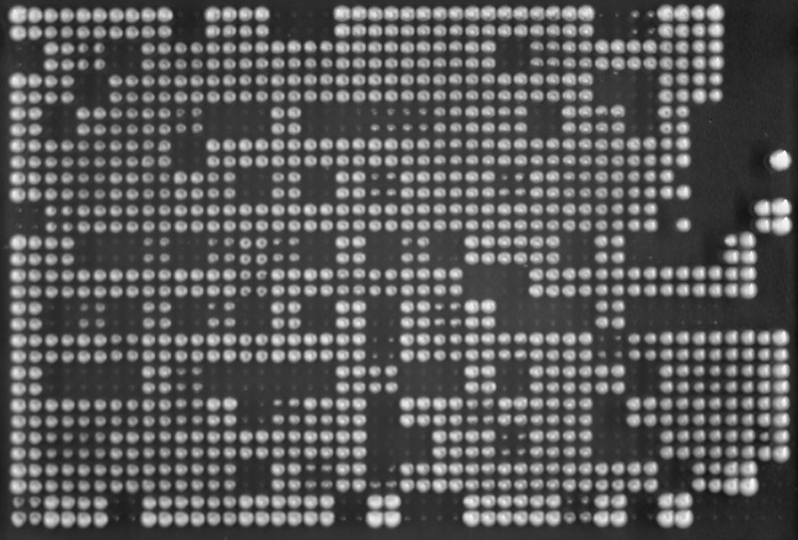

Supplement: Supplemental Material [file supp_g3.116.032607_FileS2.zip › IndividualImagesForSupplementalFile2/pse_B2-TF2-no3AT-1536-3days.1sc.png]

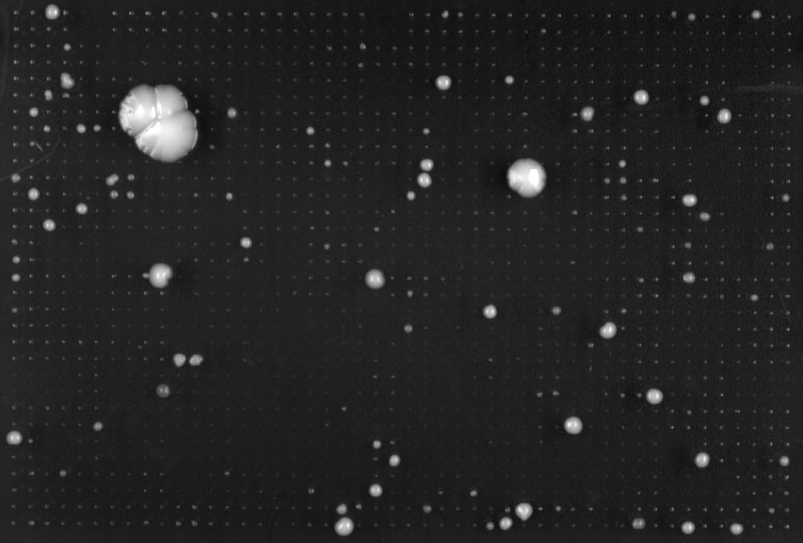

Supplement: Supplemental Material [file supp_g3.116.032607_FileS2.zip › IndividualImagesForSupplementalFile2/pse_B3-TF1-10mM3AT-after10days.png]

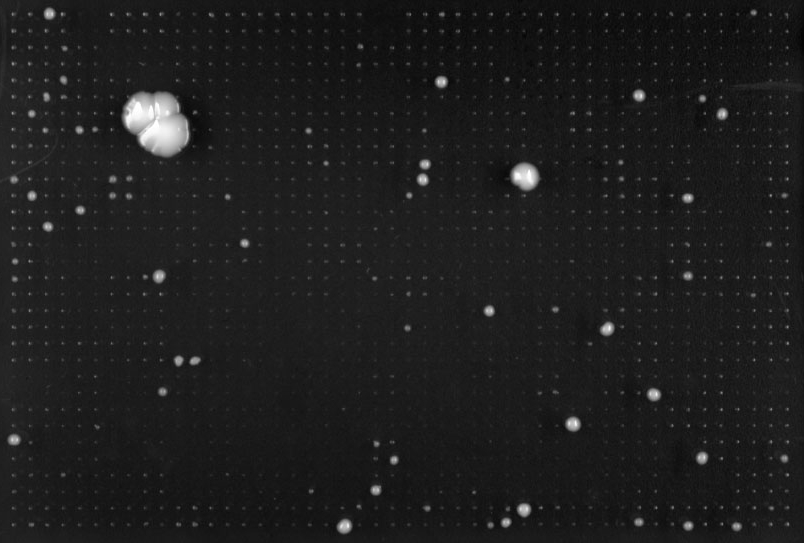

Supplement: Supplemental Material [file supp_g3.116.032607_FileS2.zip › IndividualImagesForSupplementalFile2/pse_B3-TF1-10mM3AT-after7days.1sc.png]

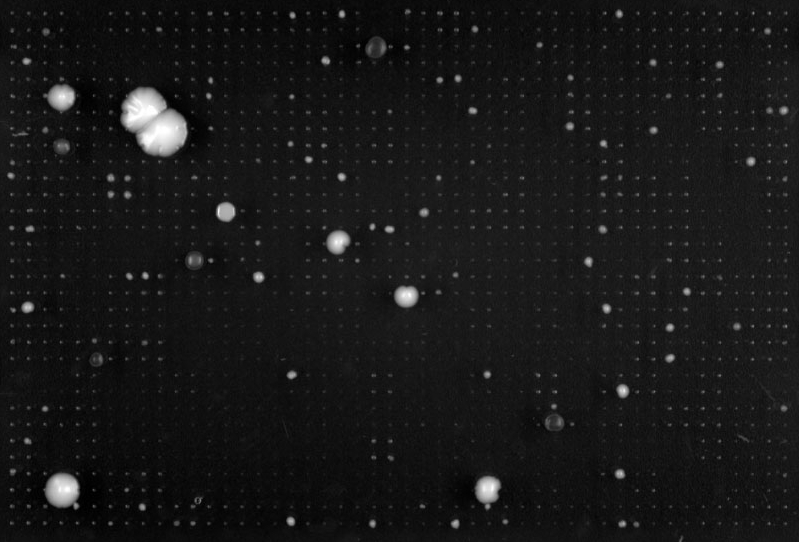

Supplement: Supplemental Material [file supp_g3.116.032607_FileS2.zip › IndividualImagesForSupplementalFile2/pse_B3-TF1-20mM3AT-after10days.png]

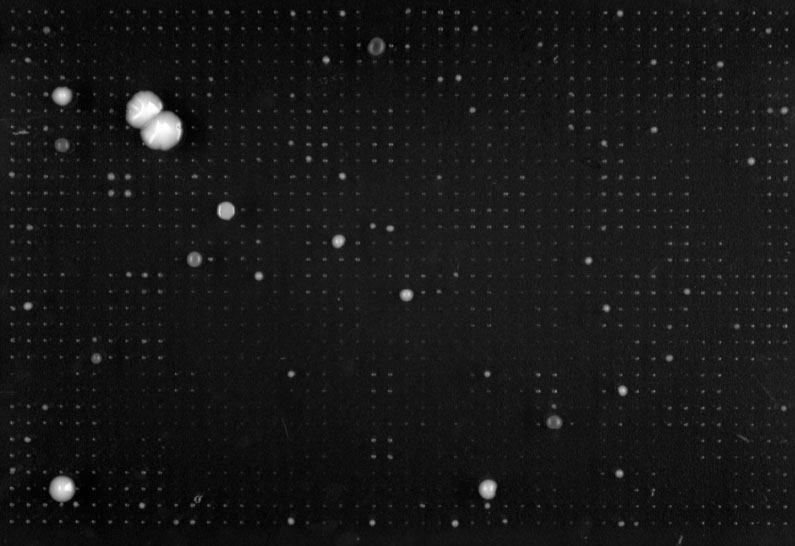

Supplement: Supplemental Material [file supp_g3.116.032607_FileS2.zip › IndividualImagesForSupplementalFile2/pse_B3-TF1-20mM3AT-after7days.1sc.png]

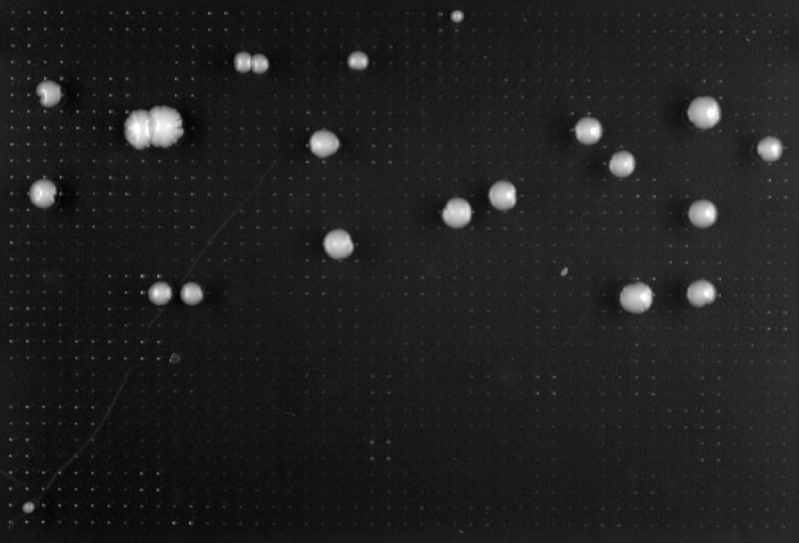

Supplement: Supplemental Material [file supp_g3.116.032607_FileS2.zip › IndividualImagesForSupplementalFile2/pse_B3-TF1-40mM3AT-after10days.png]

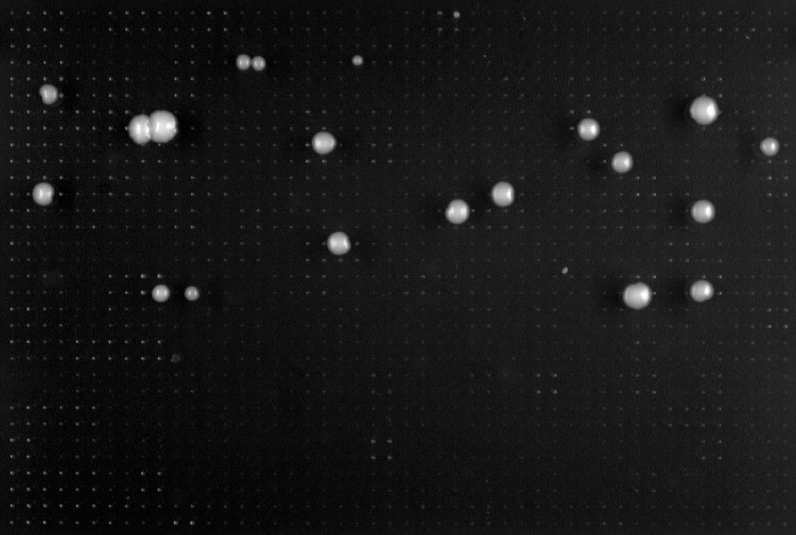

Supplement: Supplemental Material [file supp_g3.116.032607_FileS2.zip › IndividualImagesForSupplementalFile2/pse_B3-TF1-40mM3AT-after7days.1sc.png]

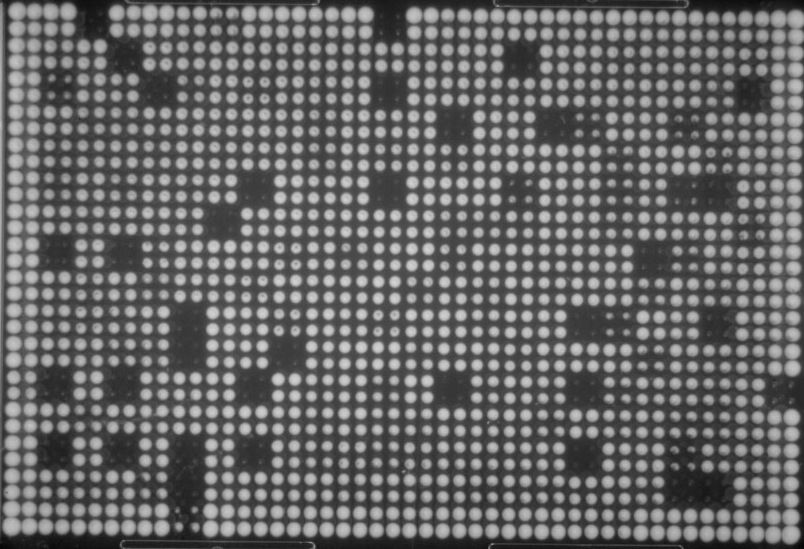

Supplement: Supplemental Material [file supp_g3.116.032607_FileS2.zip › IndividualImagesForSupplementalFile2/pse_B3-TF1-no3AT-1536-3days.1sc.png]

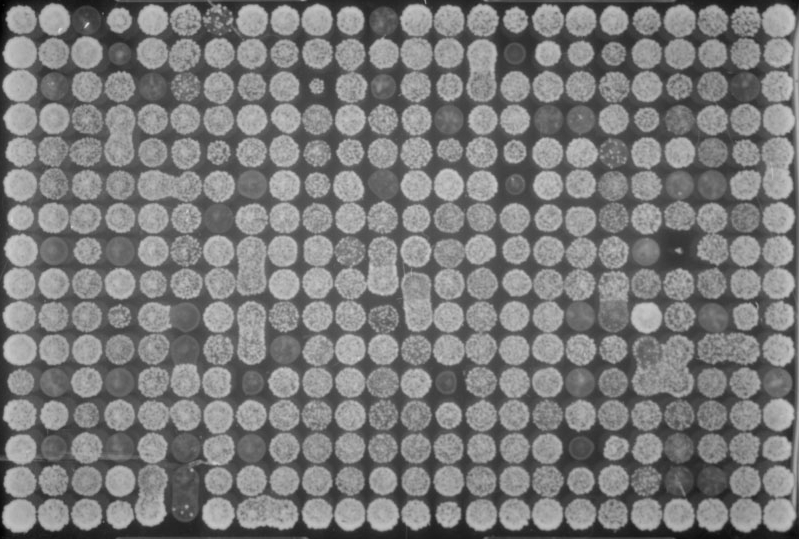

Supplement: Supplemental Material [file supp_g3.116.032607_FileS2.zip › IndividualImagesForSupplementalFile2/pse_B3-TF1-no3AT-after3days.1sc.png]

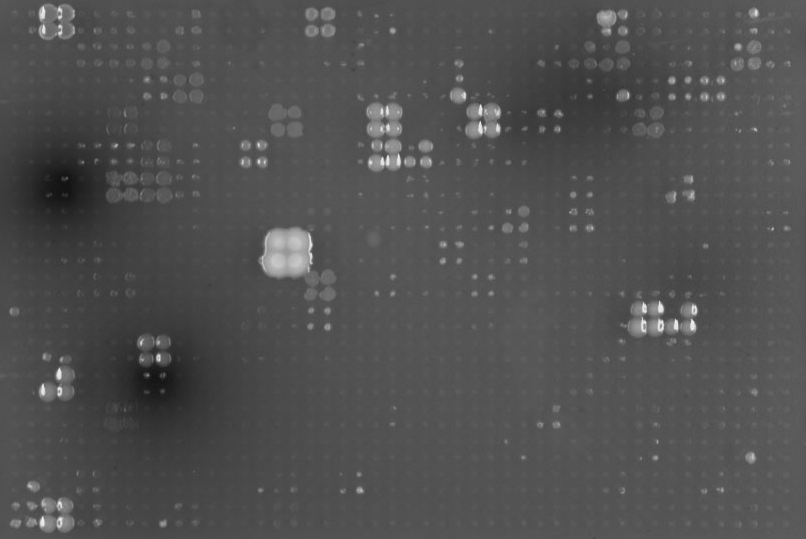

Supplement: Supplemental Material [file supp_g3.116.032607_FileS2.zip › IndividualImagesForSupplementalFile2/pse_B3-TF2-10mM3AT-9days-rep.1sc.png]

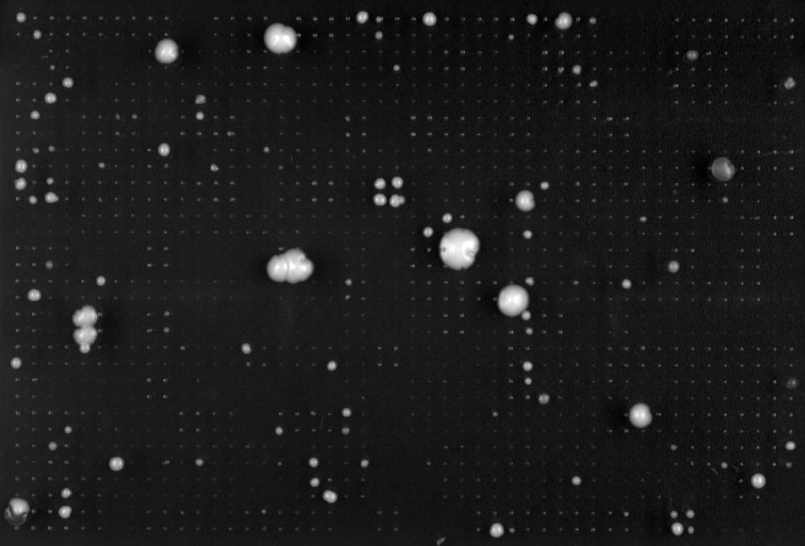

Supplement: Supplemental Material [file supp_g3.116.032607_FileS2.zip › IndividualImagesForSupplementalFile2/pse_B3-TF2-10mM3AT-after10days.png]

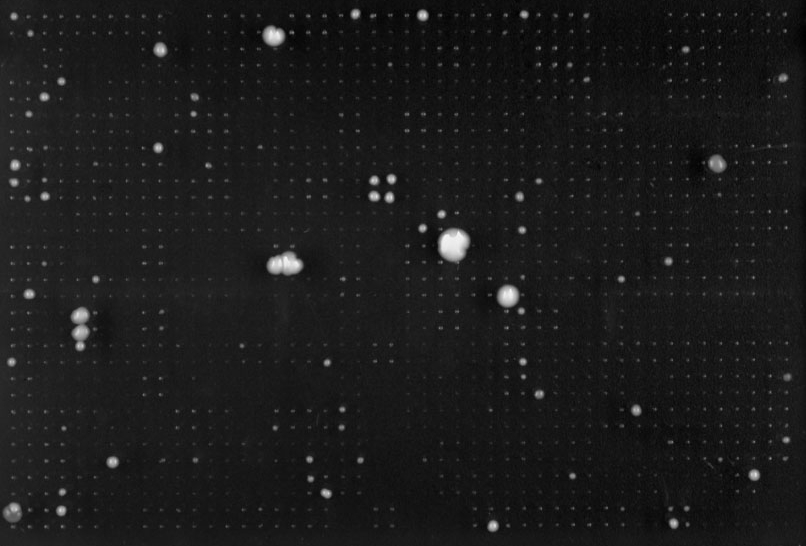

Supplement: Supplemental Material [file supp_g3.116.032607_FileS2.zip › IndividualImagesForSupplementalFile2/pse_B3-TF2-10mM3AT-after7days.1sc.png]

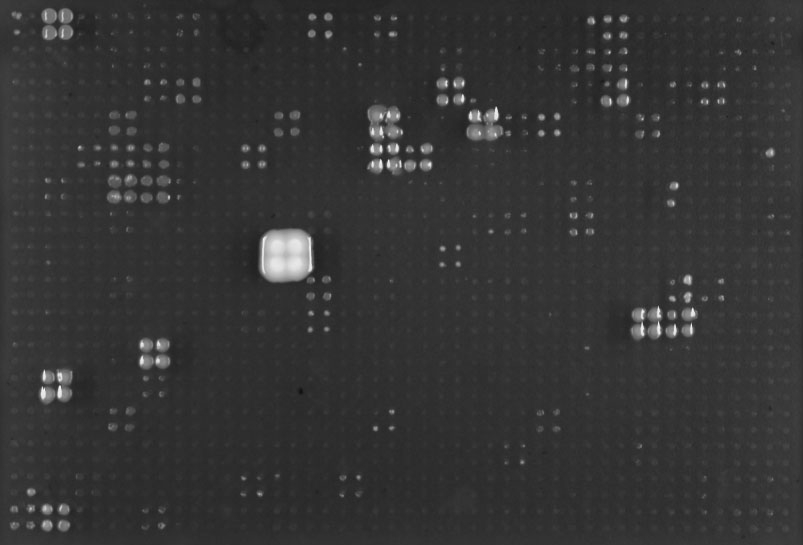

Supplement: Supplemental Material [file supp_g3.116.032607_FileS2.zip › IndividualImagesForSupplementalFile2/pse_B3-TF2-20mM3AT-9days-rep.1sc.png]

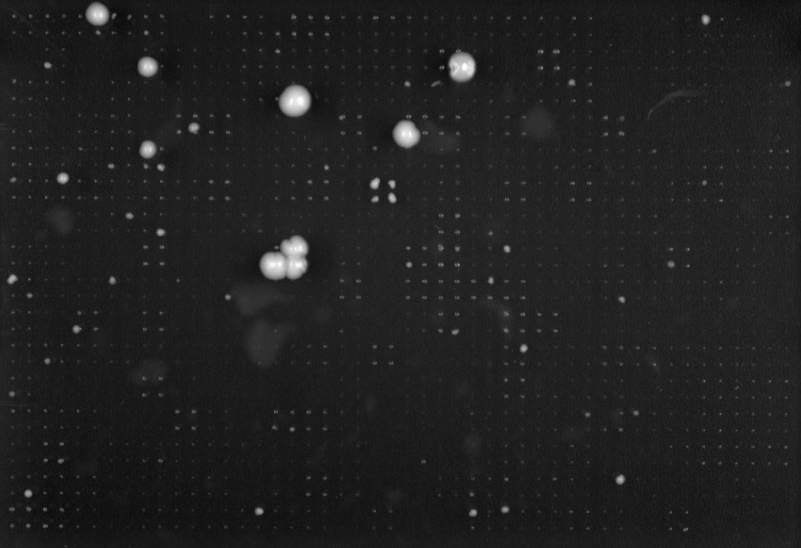

Supplement: Supplemental Material [file supp_g3.116.032607_FileS2.zip › IndividualImagesForSupplementalFile2/pse_B3-TF2-20mM3AT-after10days.png]

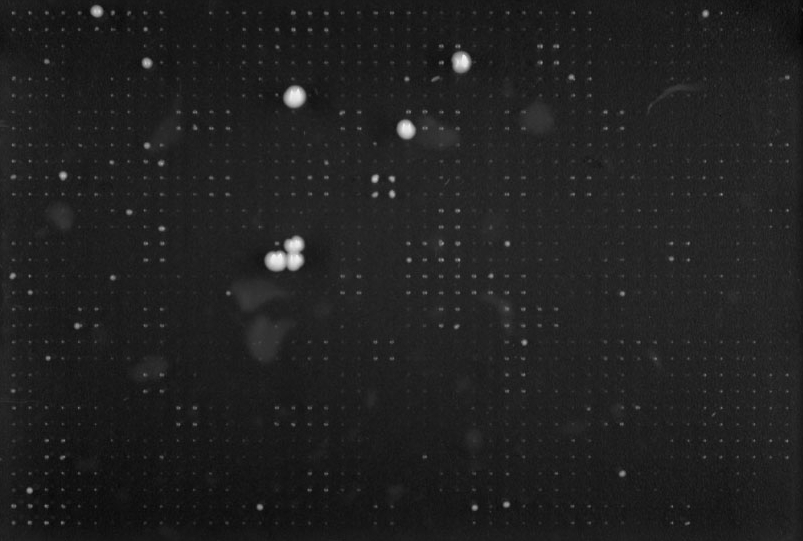

Supplement: Supplemental Material [file supp_g3.116.032607_FileS2.zip › IndividualImagesForSupplementalFile2/pse_B3-TF2-20mM3AT-after7days.1sc.png]

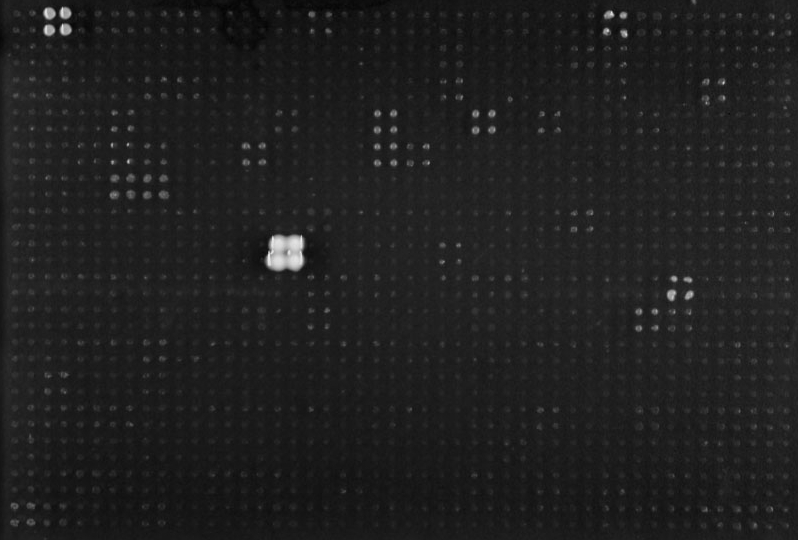

Supplement: Supplemental Material [file supp_g3.116.032607_FileS2.zip › IndividualImagesForSupplementalFile2/pse_B3-TF2-40mM3AT-9days-rep.1sc.png]

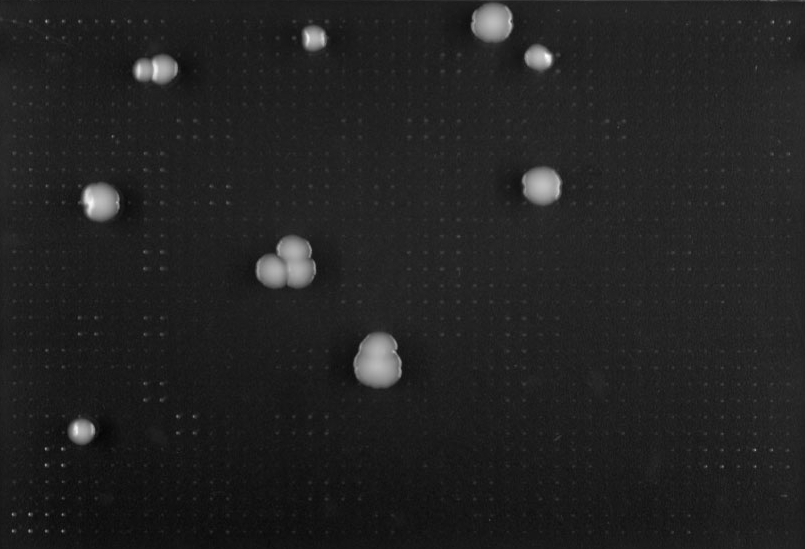

Supplement: Supplemental Material [file supp_g3.116.032607_FileS2.zip › IndividualImagesForSupplementalFile2/pse_B3-TF2-40mM3AT-after10days.png]

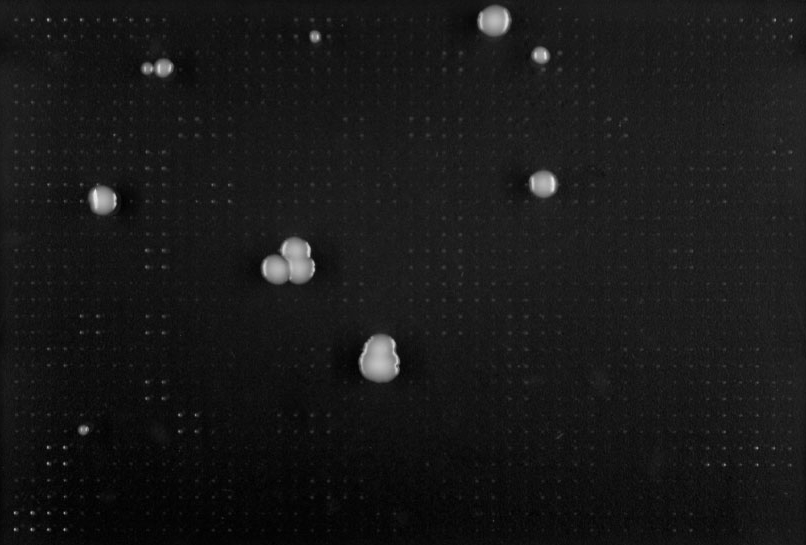

Supplement: Supplemental Material [file supp_g3.116.032607_FileS2.zip › IndividualImagesForSupplementalFile2/pse_B3-TF2-40mM3AT-after7days.1sc.png]

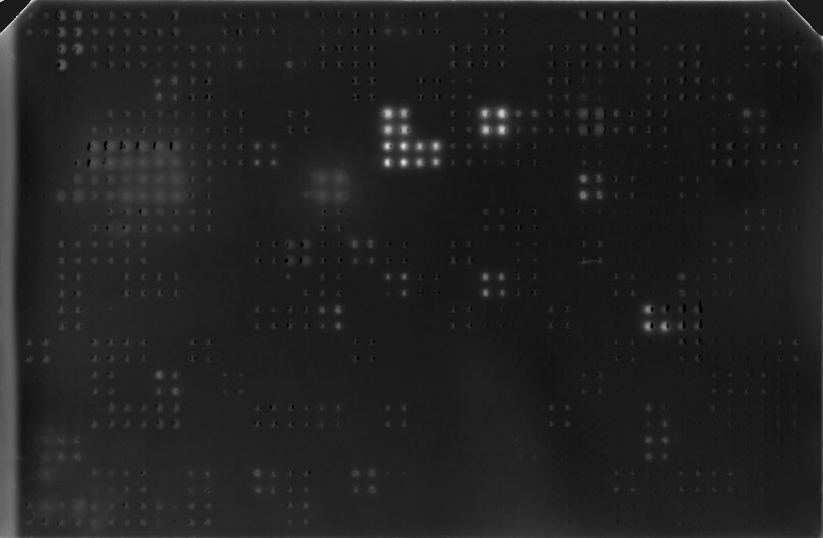

Supplement: Supplemental Material [file supp_g3.116.032607_FileS2.zip › IndividualImagesForSupplementalFile2/pse_B3-TF2-LacZ-48hrs-contam.1sc.png]

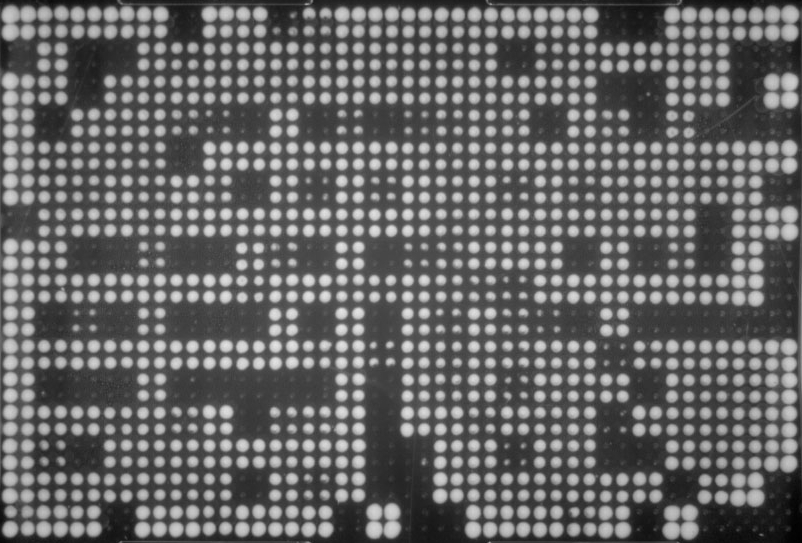

Supplement: Supplemental Material [file supp_g3.116.032607_FileS2.zip › IndividualImagesForSupplementalFile2/pse_B3-TF2-no3AT-1536-3days.1sc.png]

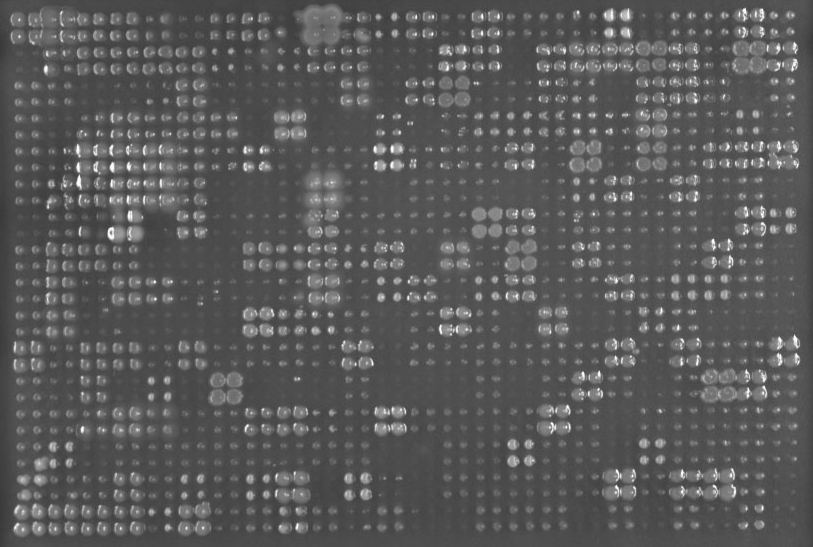

Supplement: Supplemental Material [file supp_g3.116.032607_FileS2.zip › IndividualImagesForSupplementalFile2/pse_B3-TF2-no3ATrep1536-6dycnt.png]

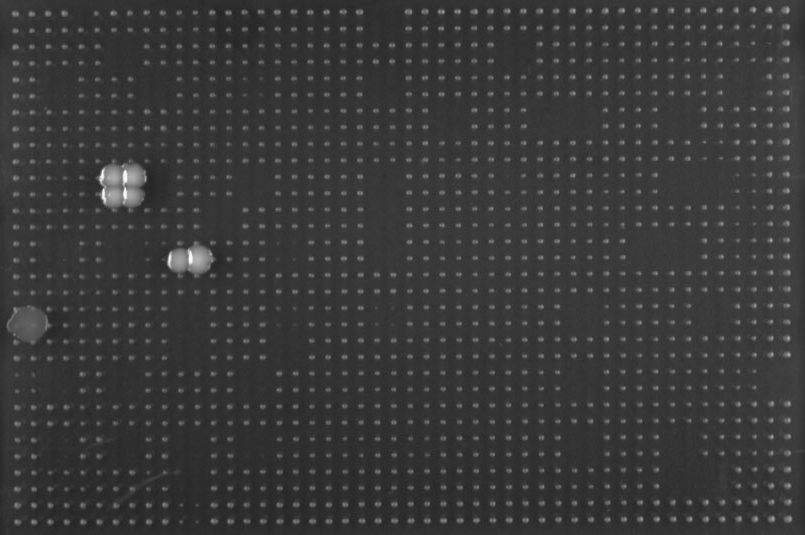

Supplement: Supplemental Material [file supp_g3.116.032607_FileS2.zip › IndividualImagesForSupplementalFile2/pse_B4-TF1-10mM3AT-after10days.png]

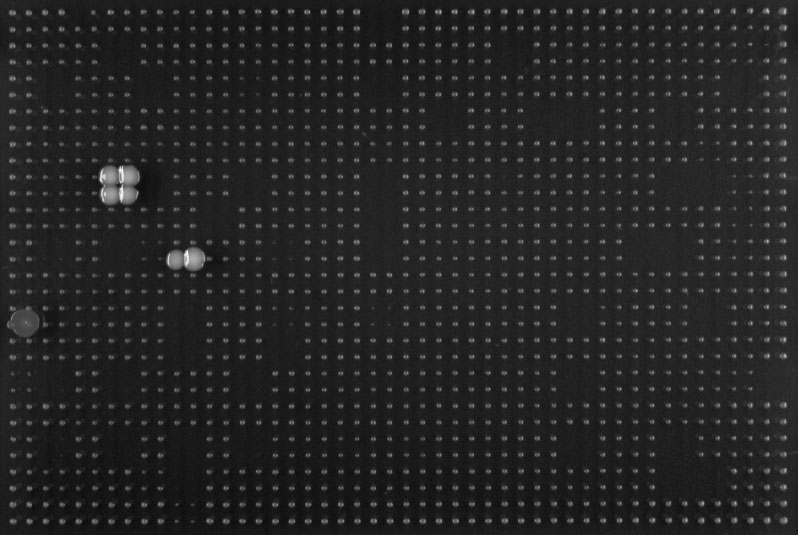

Supplement: Supplemental Material [file supp_g3.116.032607_FileS2.zip › IndividualImagesForSupplementalFile2/pse_B4-TF1-10mM3AT-after7days.1sc.png]

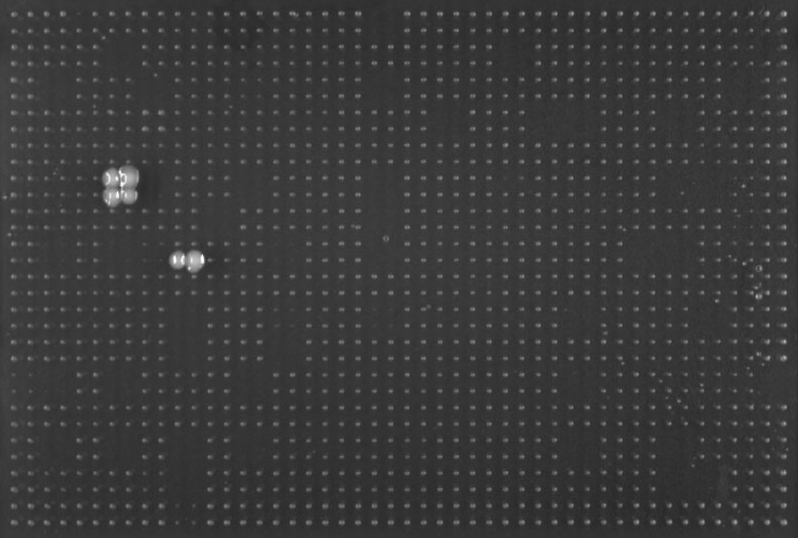

Supplement: Supplemental Material [file supp_g3.116.032607_FileS2.zip › IndividualImagesForSupplementalFile2/pse_B4-TF1-20mM3AT-after10days.png]

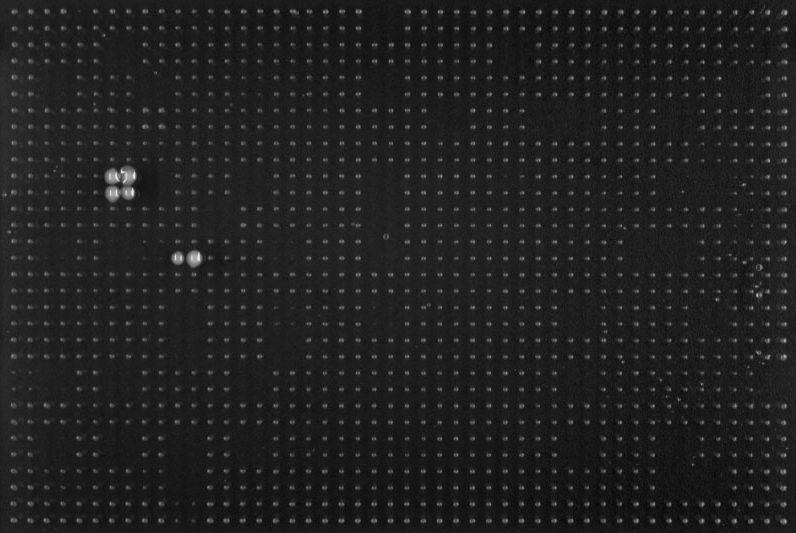

Supplement: Supplemental Material [file supp_g3.116.032607_FileS2.zip › IndividualImagesForSupplementalFile2/pse_B4-TF1-20mM3AT-after7days.1sc.png]

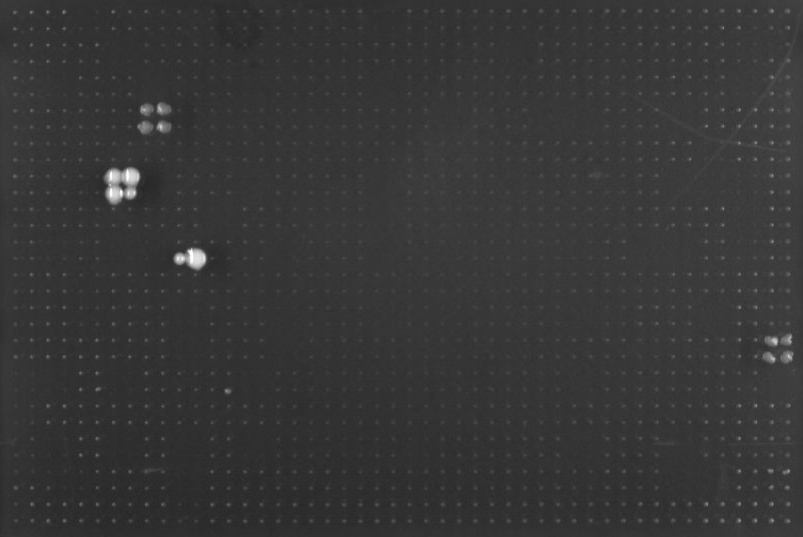

Supplement: Supplemental Material [file supp_g3.116.032607_FileS2.zip › IndividualImagesForSupplementalFile2/pse_B4-TF1-60mM3AT-after10days.png]

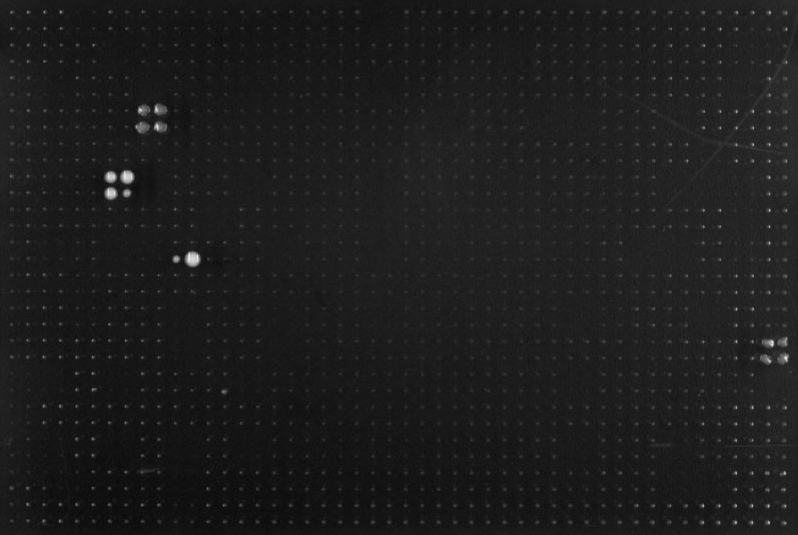

Supplement: Supplemental Material [file supp_g3.116.032607_FileS2.zip › IndividualImagesForSupplementalFile2/pse_B4-TF1-60mM3AT-after7days.1sc.png]

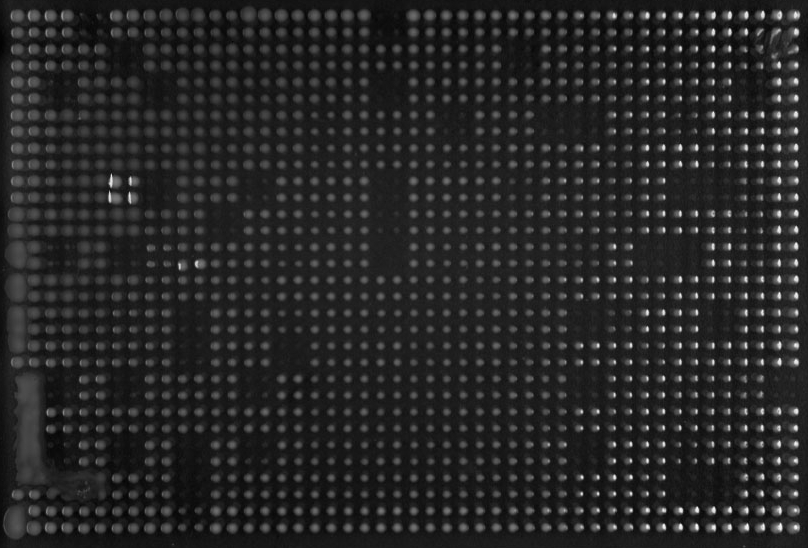

Supplement: Supplemental Material [file supp_g3.116.032607_FileS2.zip › IndividualImagesForSupplementalFile2/pse_B4-TF1-no3AT-1536-3days.1sc.png]

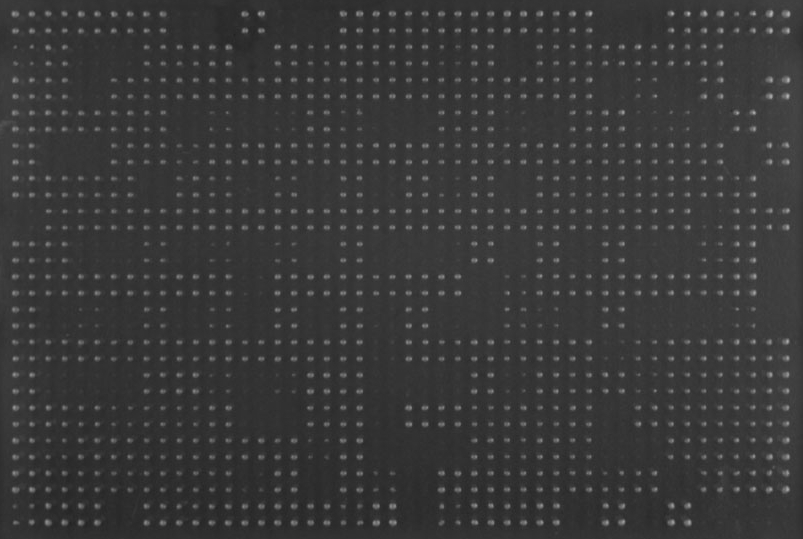

Supplement: Supplemental Material [file supp_g3.116.032607_FileS2.zip › IndividualImagesForSupplementalFile2/pse_B4-TF2-10mM3AT-after10days.png]

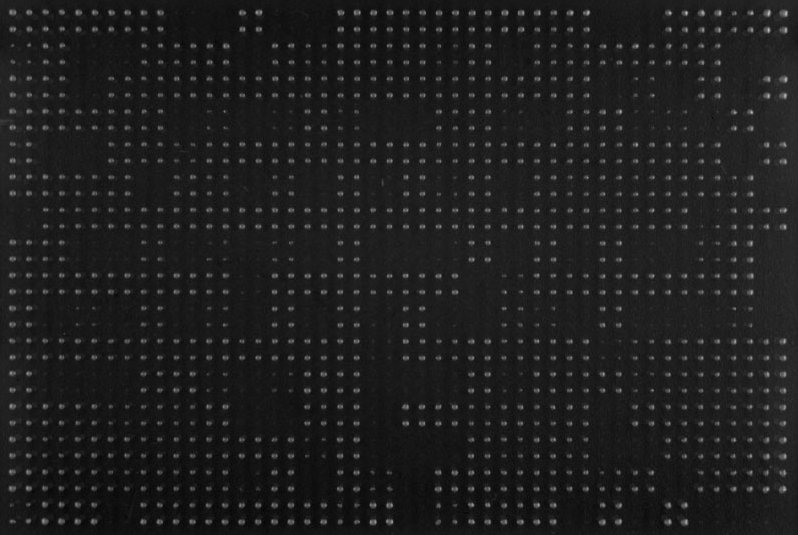

Supplement: Supplemental Material [file supp_g3.116.032607_FileS2.zip › IndividualImagesForSupplementalFile2/pse_B4-TF2-10mM3AT-after7days.1sc.png]

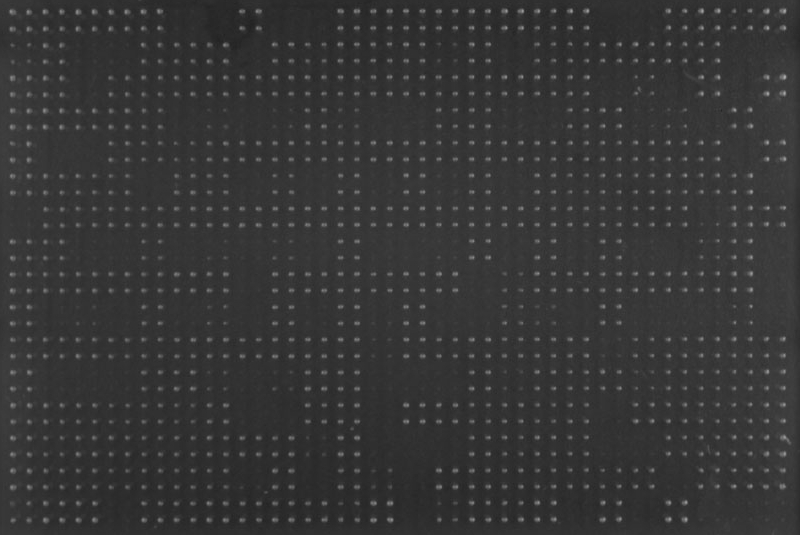

Supplement: Supplemental Material [file supp_g3.116.032607_FileS2.zip › IndividualImagesForSupplementalFile2/pse_B4-TF2-20mM3AT-after10days.png]

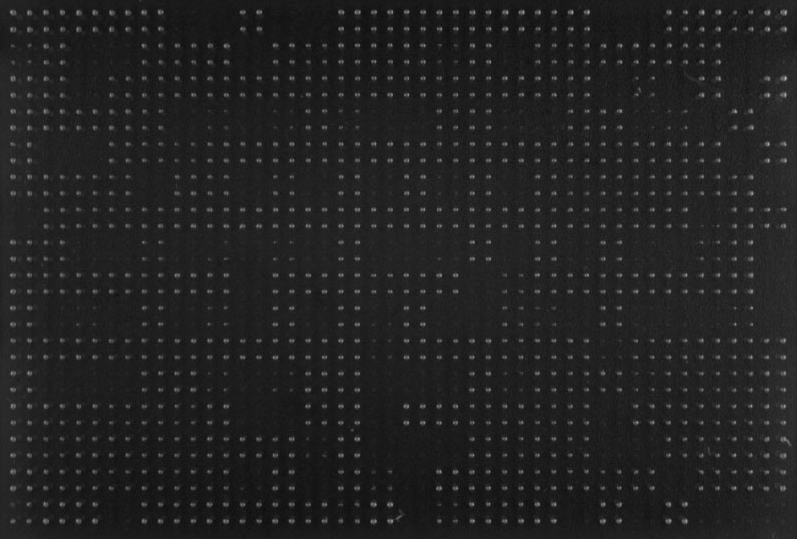

Supplement: Supplemental Material [file supp_g3.116.032607_FileS2.zip › IndividualImagesForSupplementalFile2/pse_B4-TF2-20mM3AT-after7days.1sc.png]

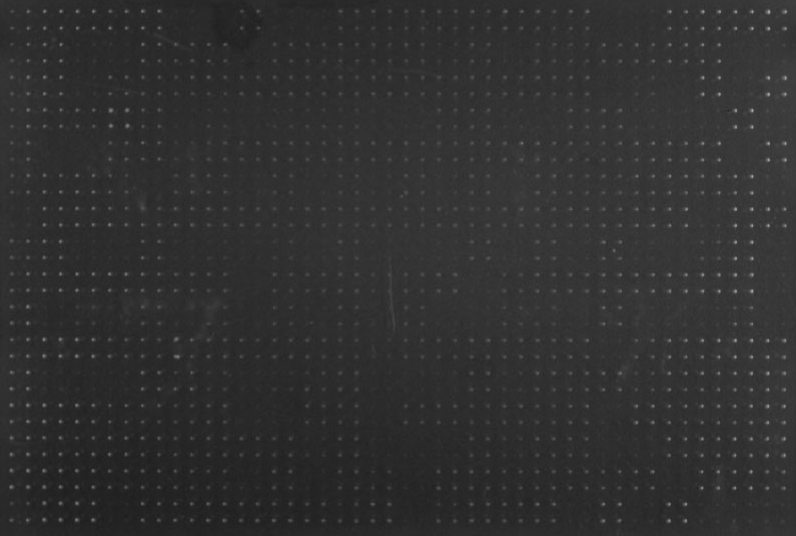

Supplement: Supplemental Material [file supp_g3.116.032607_FileS2.zip › IndividualImagesForSupplementalFile2/pse_B4-TF2-60mM3AT-after10days.png]

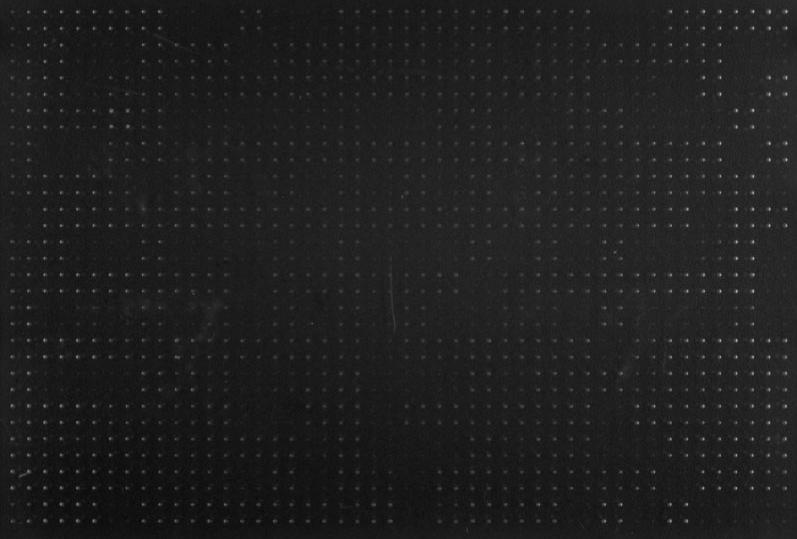

Supplement: Supplemental Material [file supp_g3.116.032607_FileS2.zip › IndividualImagesForSupplementalFile2/pse_B4-TF2-60mM3AT-after7days.1sc.png]

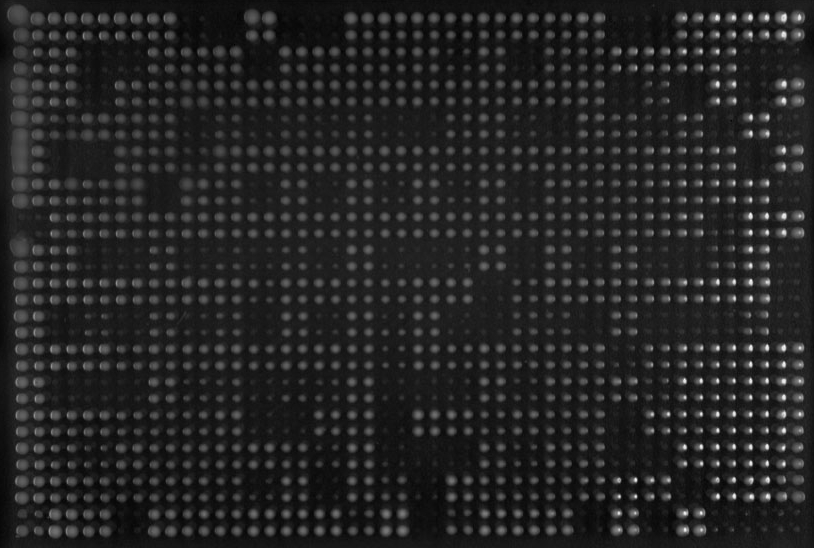

Supplement: Supplemental Material [file supp_g3.116.032607_FileS2.zip › IndividualImagesForSupplementalFile2/pse_B4-TF2-no3AT-1536-3days.1sc.png]

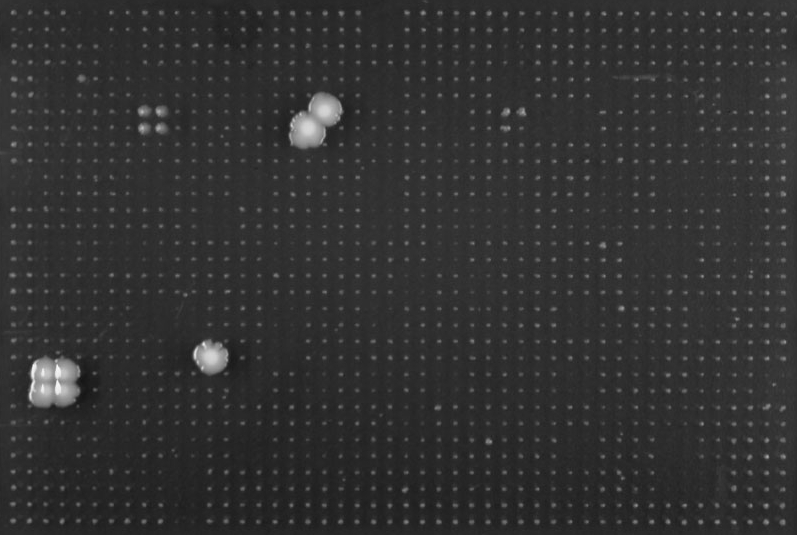

Supplement: Supplemental Material [file supp_g3.116.032607_FileS2.zip › IndividualImagesForSupplementalFile2/pse_B5-TF1-10mM3AT-after10days.png]

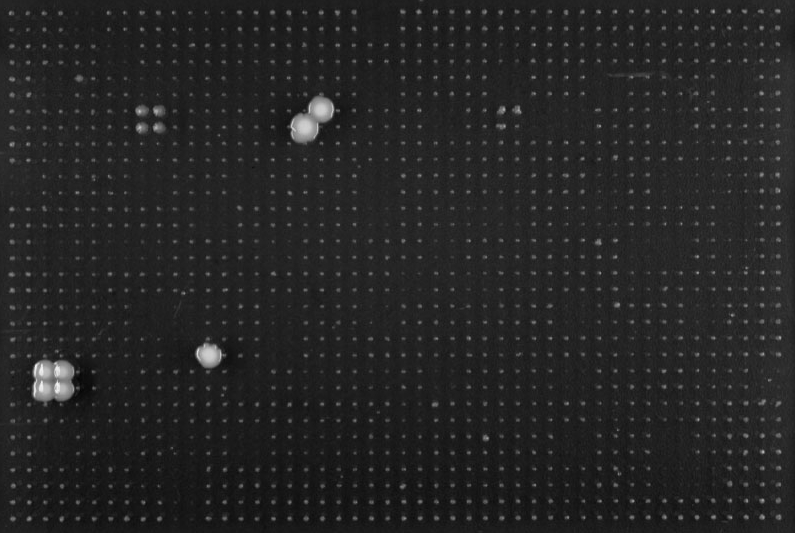

Supplement: Supplemental Material [file supp_g3.116.032607_FileS2.zip › IndividualImagesForSupplementalFile2/pse_B5-TF1-10mM3AT-after7days.1sc.png]

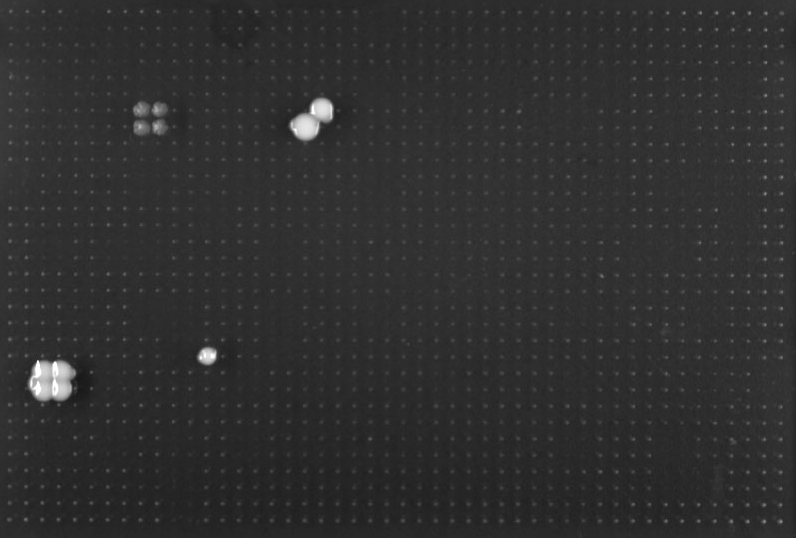

Supplement: Supplemental Material [file supp_g3.116.032607_FileS2.zip › IndividualImagesForSupplementalFile2/pse_B5-TF1-40mM3AT-after10days.png]

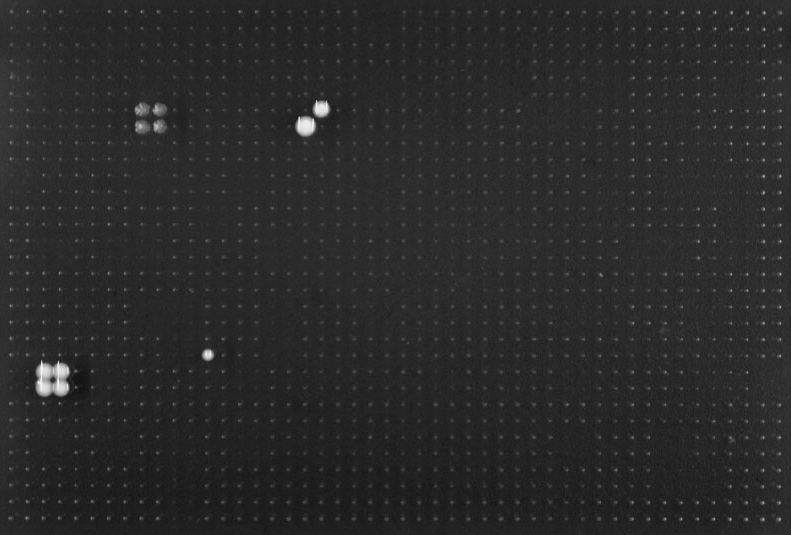

Supplement: Supplemental Material [file supp_g3.116.032607_FileS2.zip › IndividualImagesForSupplementalFile2/pse_B5-TF1-40mM3AT-after7days.1sc.png]

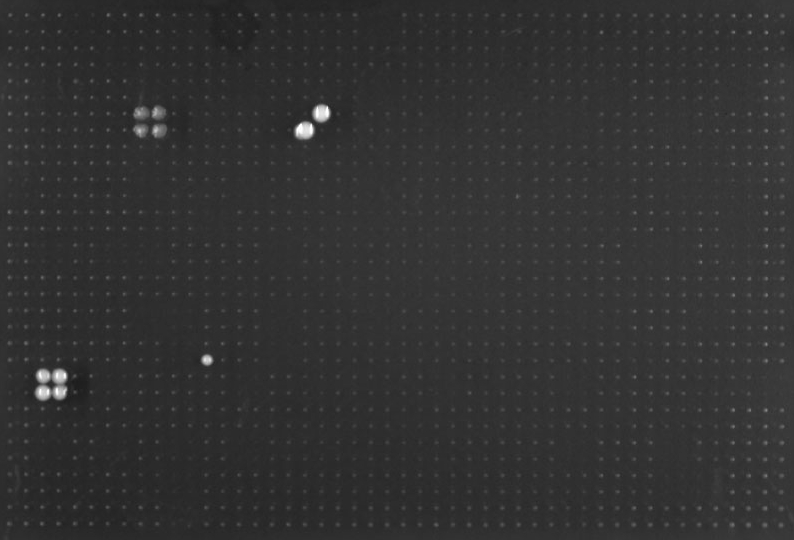

Supplement: Supplemental Material [file supp_g3.116.032607_FileS2.zip › IndividualImagesForSupplementalFile2/pse_B5-TF1-60mM3AT-after10days.png]
