# Supplementary figures and images for: Potential Direct Regulators of the Drosophila yellow Gene Identified by Yeast One-Hybrid and RNAi Screens (part 1 of 3)
Source: G3 (Bethesda). 2016 Aug 12;6(10):3419–30. doi: 10.1534/g3.116.032607 (PMC5068961; doi:10.1534/g3.116.032607)

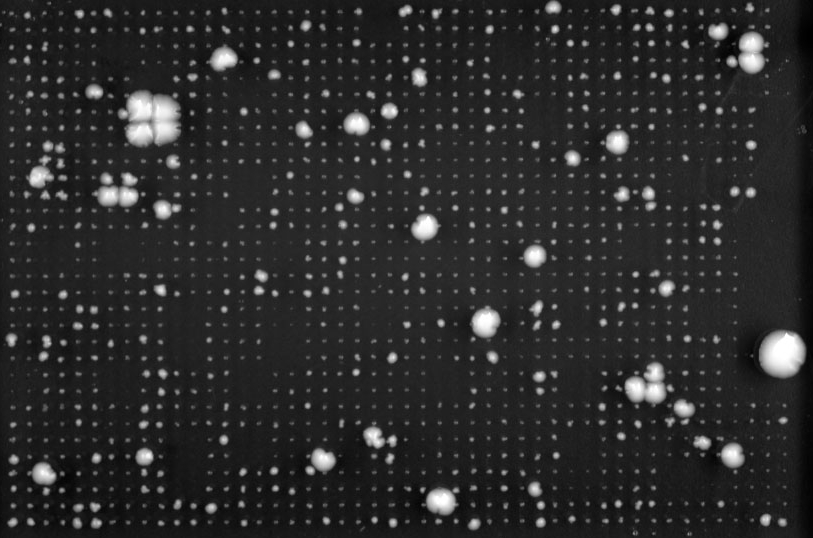

Supplement: Supplemental Material [file supp_g3.116.032607_FileS2.zip › IndividualImagesForSupplementalFile2/mel_A1-TF1-10mM3AT-10daysafter.png]

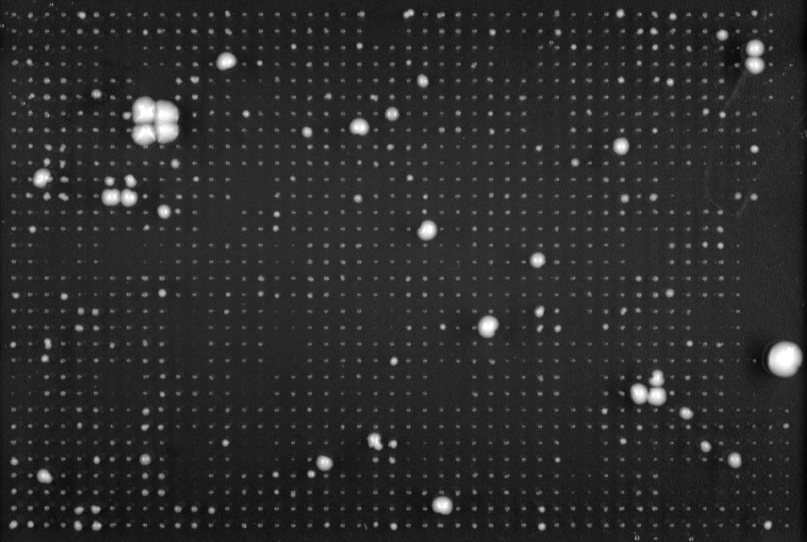

Supplement: Supplemental Material [file supp_g3.116.032607_FileS2.zip › IndividualImagesForSupplementalFile2/mel_A1-TF1-10mM3AT-7daysafter.1sc.png]

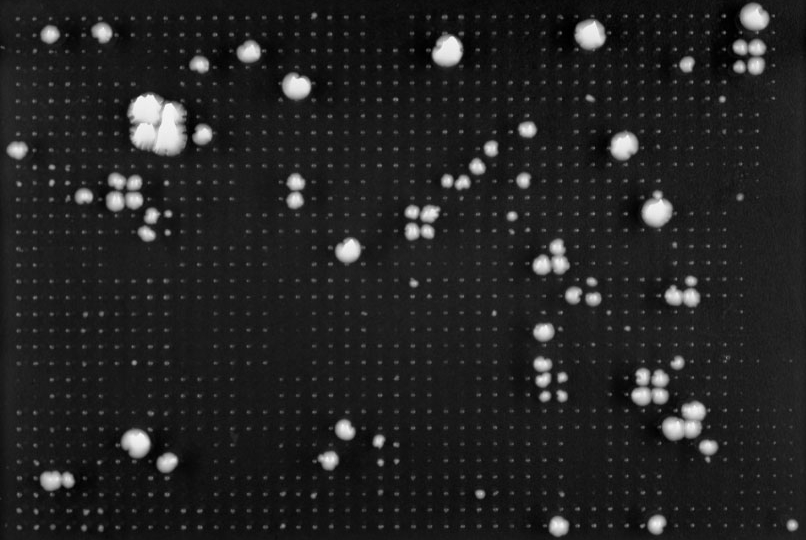

Supplement: Supplemental Material [file supp_g3.116.032607_FileS2.zip › IndividualImagesForSupplementalFile2/mel_A1-TF1-20mM3AT-10daysafter.png]

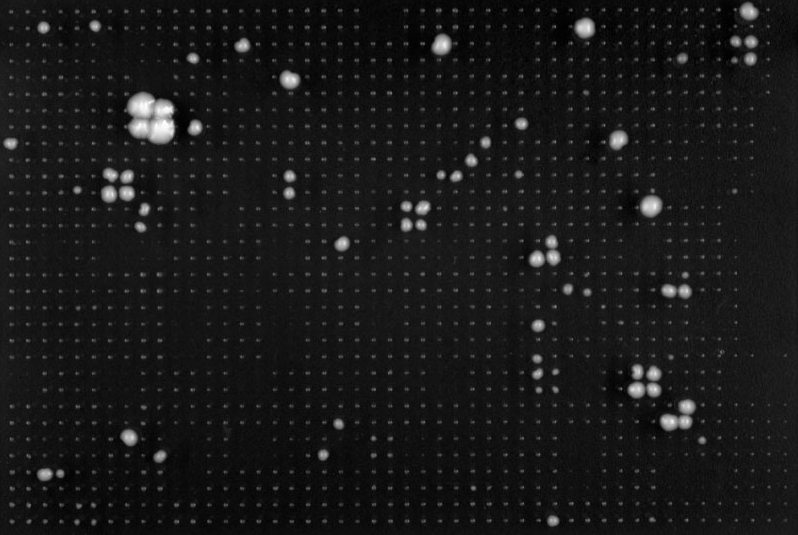

Supplement: Supplemental Material [file supp_g3.116.032607_FileS2.zip › IndividualImagesForSupplementalFile2/mel_A1-TF1-20mM3AT-7daysafter.1sc.png]

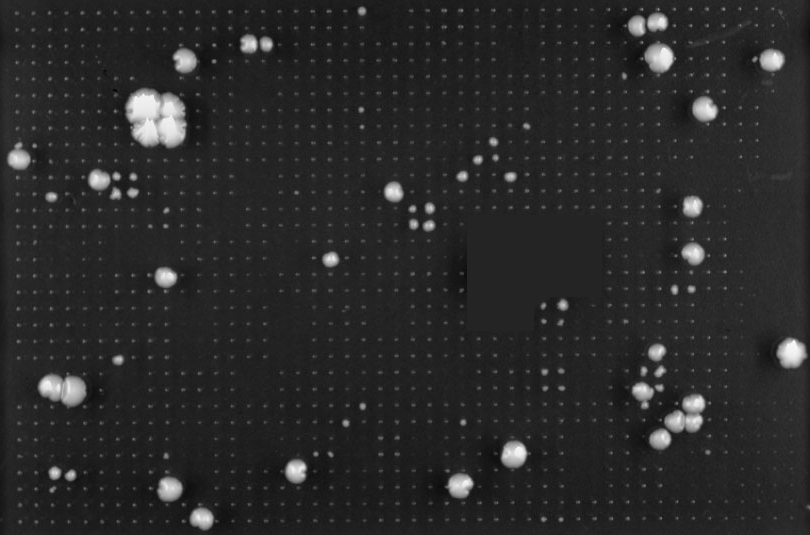

Supplement: Supplemental Material [file supp_g3.116.032607_FileS2.zip › IndividualImagesForSupplementalFile2/mel_A1-TF1-40mM3AT-10daysafter.png]

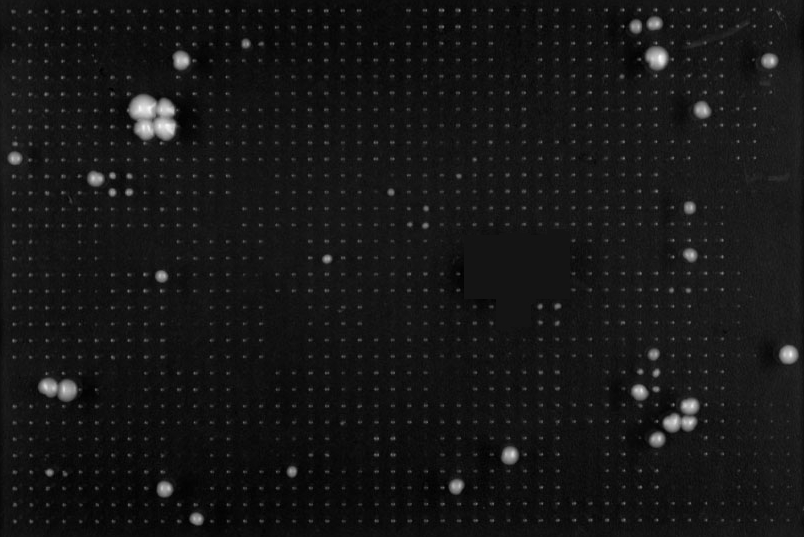

Supplement: Supplemental Material [file supp_g3.116.032607_FileS2.zip › IndividualImagesForSupplementalFile2/mel_A1-TF1-40mM3AT-7daysafter.1sc.png]

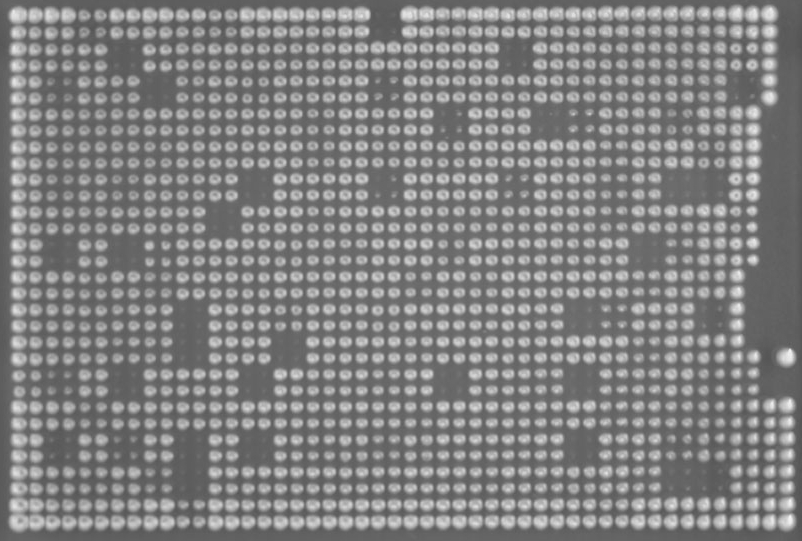

Supplement: Supplemental Material [file supp_g3.116.032607_FileS2.zip › IndividualImagesForSupplementalFile2/mel_A1-TF1-no3AT-1536-3days.1sc.png]

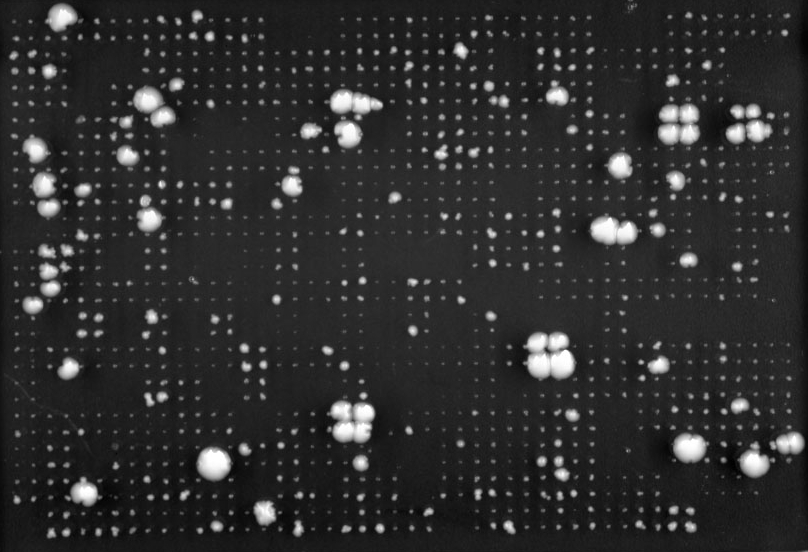

Supplement: Supplemental Material [file supp_g3.116.032607_FileS2.zip › IndividualImagesForSupplementalFile2/mel_A1-TF2-10mM3AT-10daysafter.png]

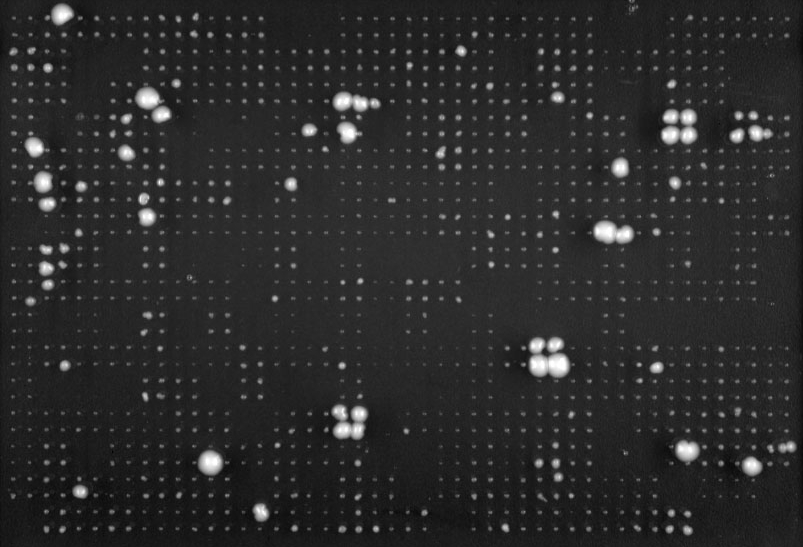

Supplement: Supplemental Material [file supp_g3.116.032607_FileS2.zip › IndividualImagesForSupplementalFile2/mel_A1-TF2-10mM3AT-7daysafter.1sc.png]

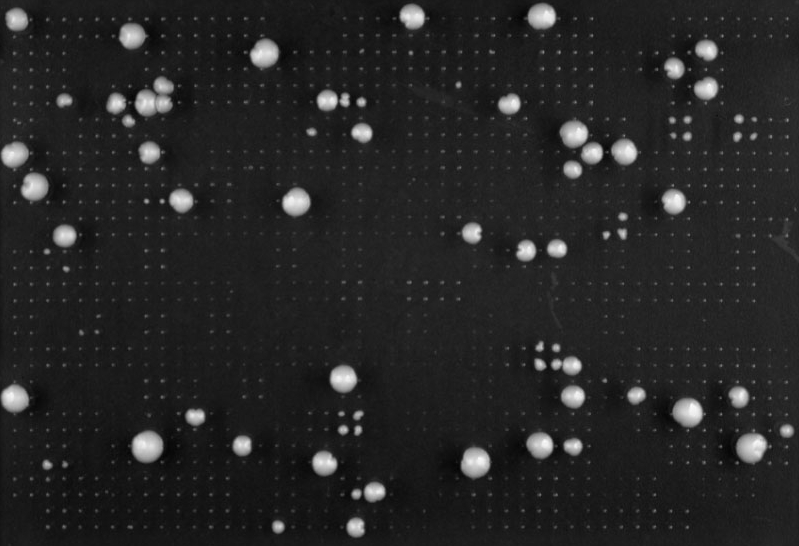

Supplement: Supplemental Material [file supp_g3.116.032607_FileS2.zip › IndividualImagesForSupplementalFile2/mel_A1-TF2-40mM3AT-10daysafter.png]

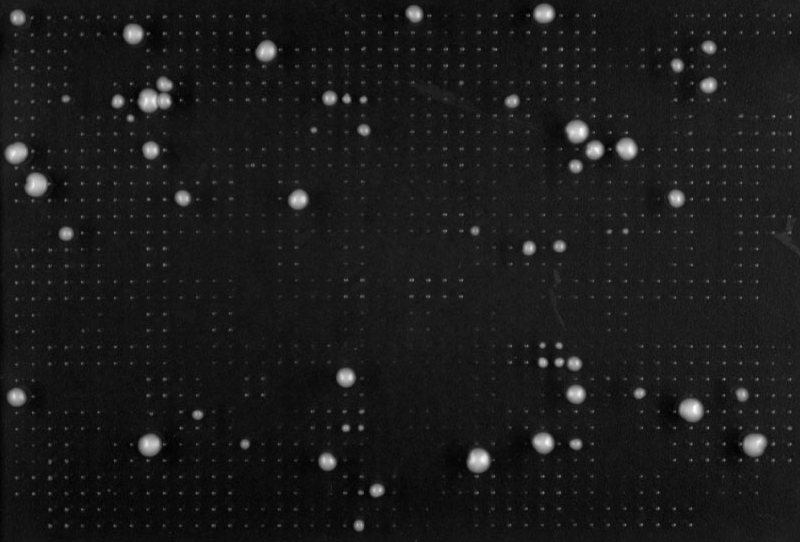

Supplement: Supplemental Material [file supp_g3.116.032607_FileS2.zip › IndividualImagesForSupplementalFile2/mel_A1-TF2-40mM3AT-7daysafter.1sc.png]

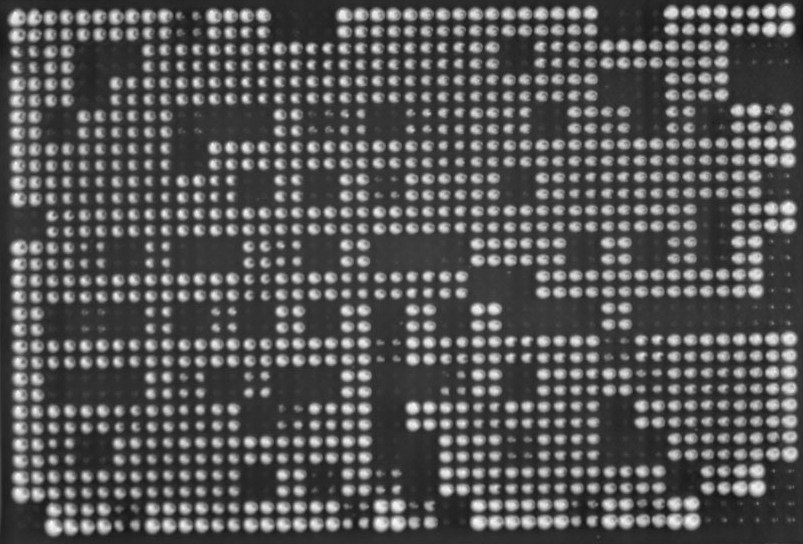

Supplement: Supplemental Material [file supp_g3.116.032607_FileS2.zip › IndividualImagesForSupplementalFile2/mel_A1-TF2-no3AT-1536-3days.1sc.png]

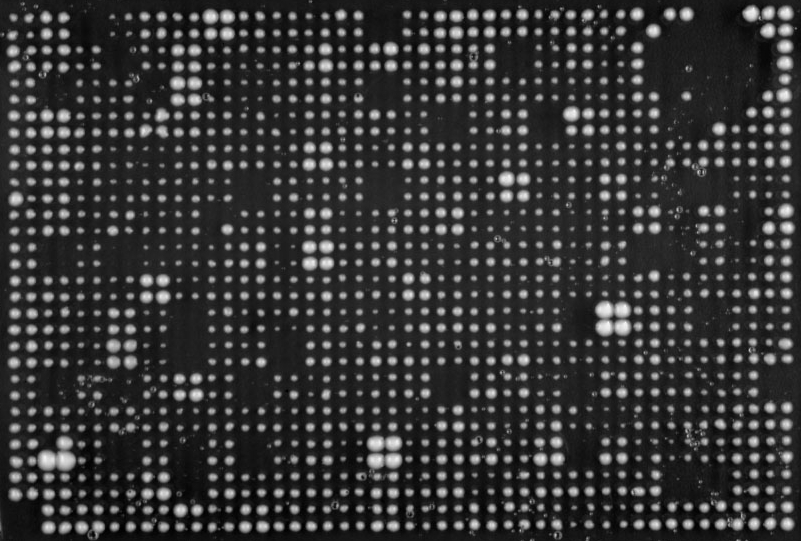

Supplement: Supplemental Material [file supp_g3.116.032607_FileS2.zip › IndividualImagesForSupplementalFile2/mel_A2-TF1-10mM3AT-after10days.png]

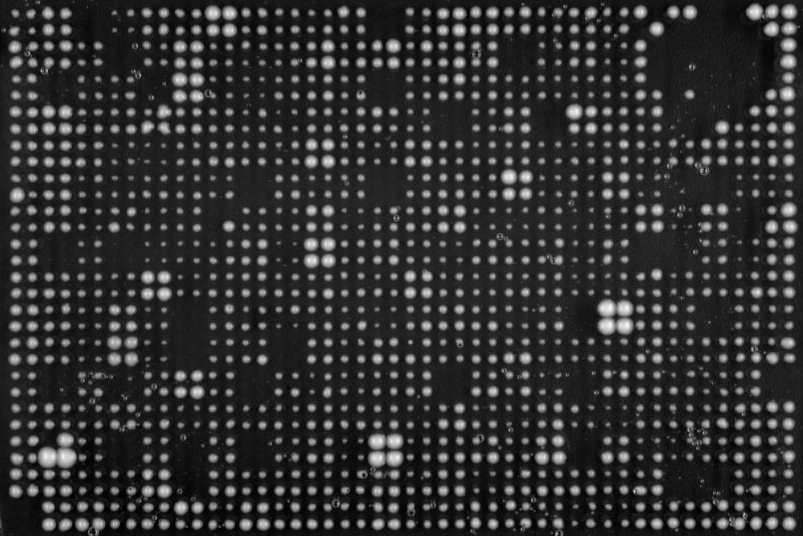

Supplement: Supplemental Material [file supp_g3.116.032607_FileS2.zip › IndividualImagesForSupplementalFile2/mel_A2-TF1-10mM3AT-after7days.1sc.png]

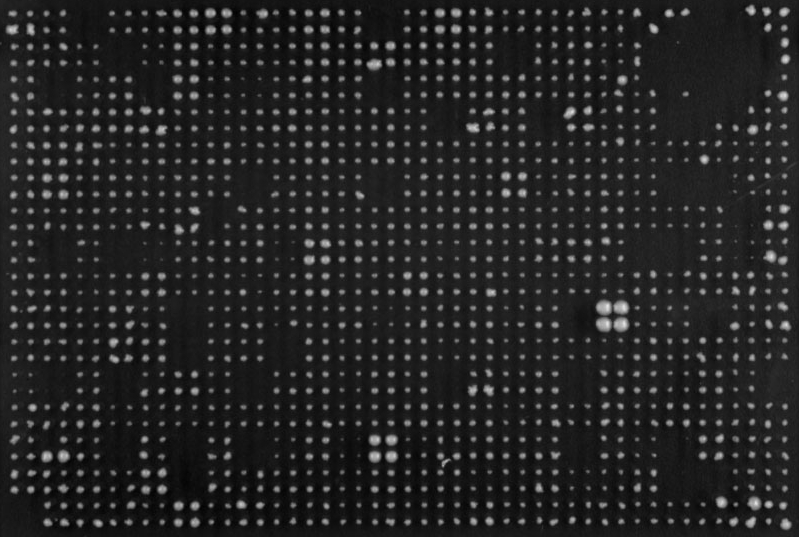

Supplement: Supplemental Material [file supp_g3.116.032607_FileS2.zip › IndividualImagesForSupplementalFile2/mel_A2-TF1-40mM3AT-after10days.png]

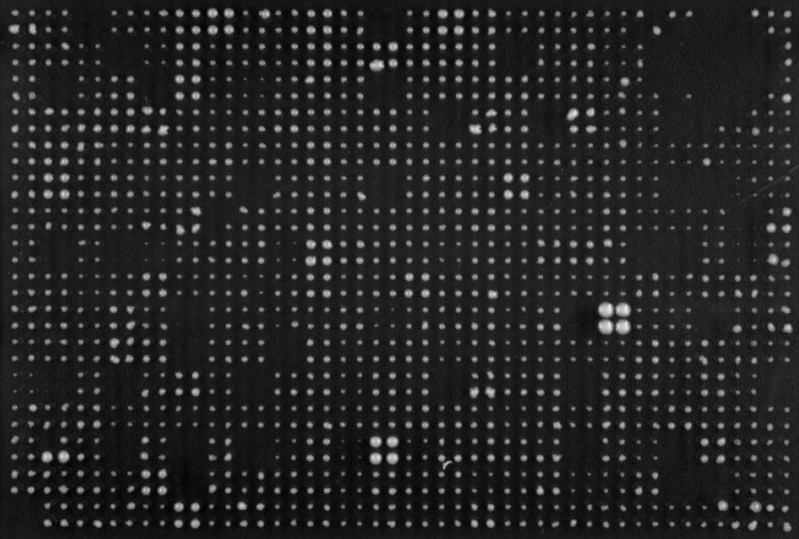

Supplement: Supplemental Material [file supp_g3.116.032607_FileS2.zip › IndividualImagesForSupplementalFile2/mel_A2-TF1-40mM3AT-after7days.1sc.png]

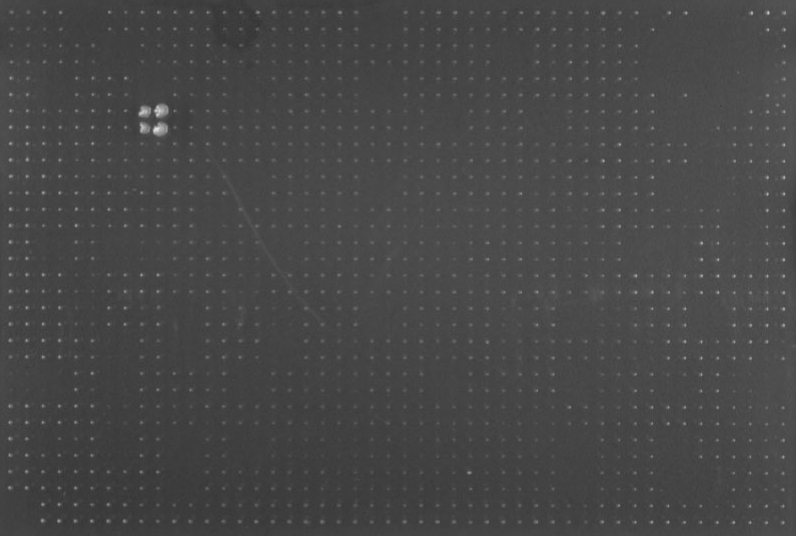

Supplement: Supplemental Material [file supp_g3.116.032607_FileS2.zip › IndividualImagesForSupplementalFile2/mel_A2-TF1-60mM3AT-after9days.1sc.png]

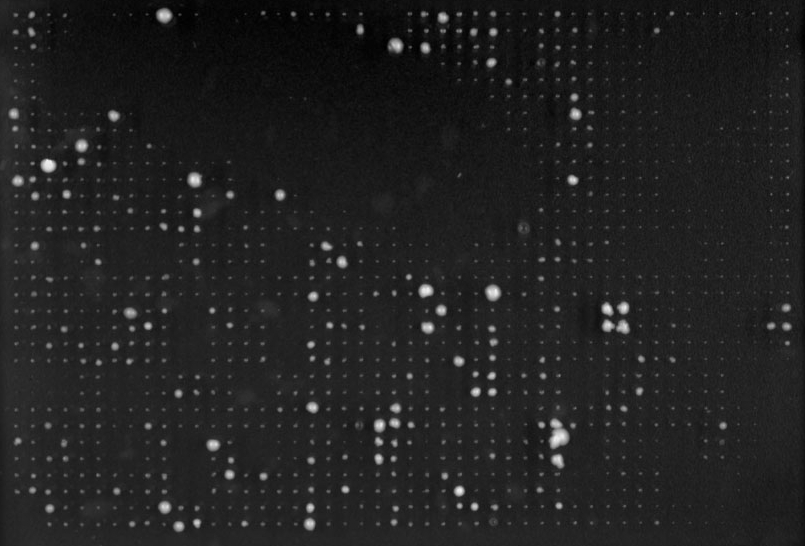

Supplement: Supplemental Material [file supp_g3.116.032607_FileS2.zip › IndividualImagesForSupplementalFile2/mel_A2-TF1-80mM3AT-after10days.png]

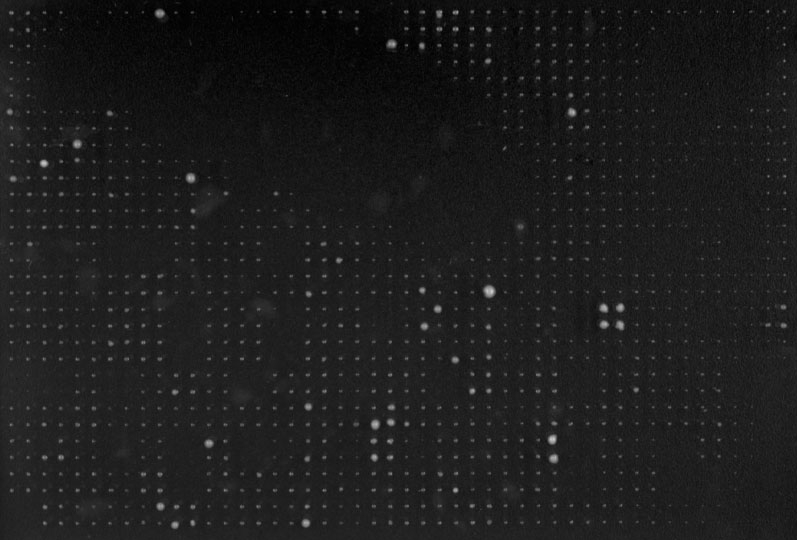

Supplement: Supplemental Material [file supp_g3.116.032607_FileS2.zip › IndividualImagesForSupplementalFile2/mel_A2-TF1-80mM3AT-after7days.1sc.png]

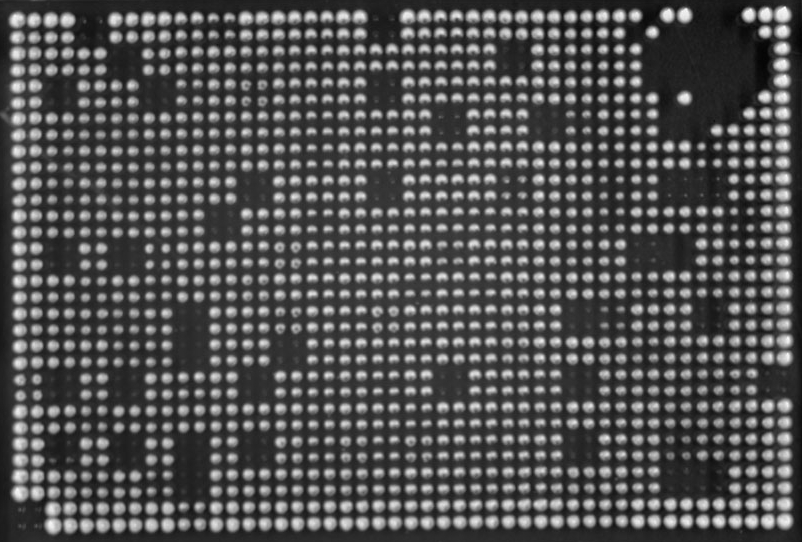

Supplement: Supplemental Material [file supp_g3.116.032607_FileS2.zip › IndividualImagesForSupplementalFile2/mel_A2-TF1-no3AT-1536-3days.1sc.png]

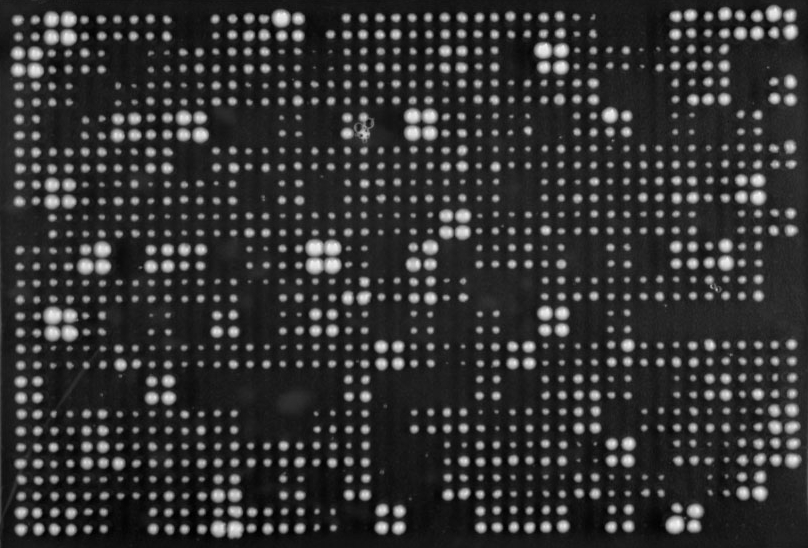

Supplement: Supplemental Material [file supp_g3.116.032607_FileS2.zip › IndividualImagesForSupplementalFile2/mel_A2-TF2-10mM3AT-after10days.png]

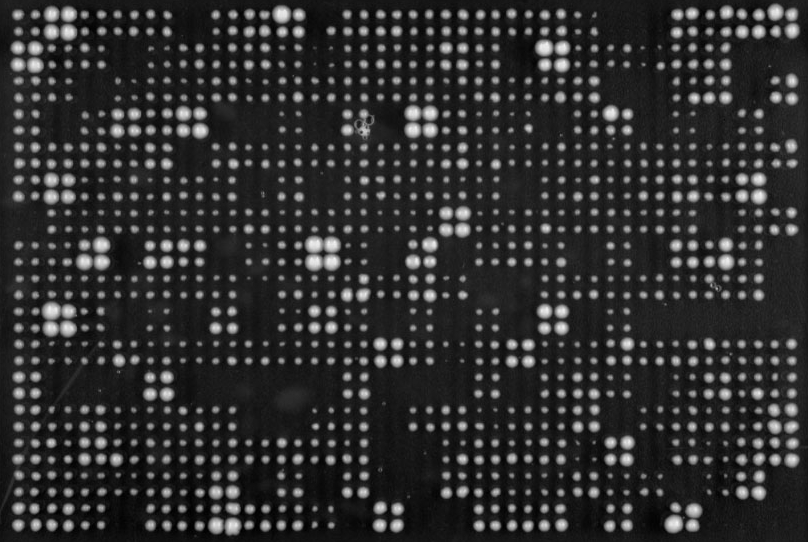

Supplement: Supplemental Material [file supp_g3.116.032607_FileS2.zip › IndividualImagesForSupplementalFile2/mel_A2-TF2-10mM3AT-after7days.1sc.png]

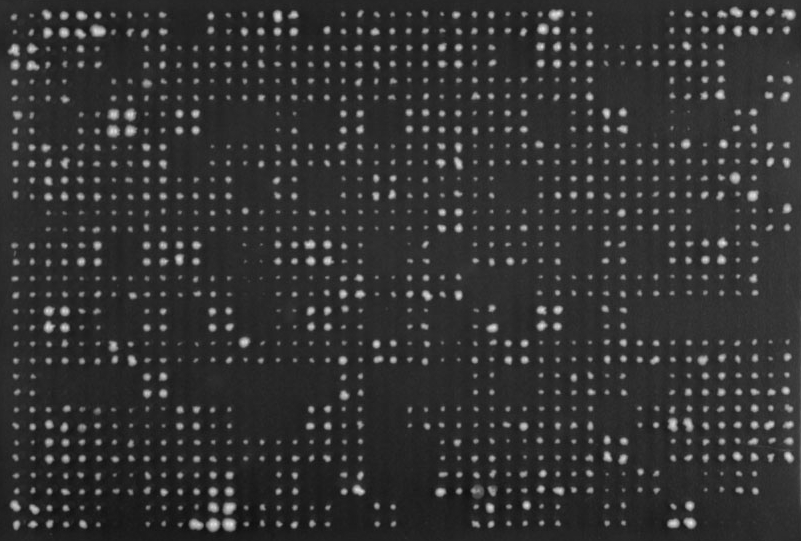

Supplement: Supplemental Material [file supp_g3.116.032607_FileS2.zip › IndividualImagesForSupplementalFile2/mel_A2-TF2-40mM3AT-after10days.png]

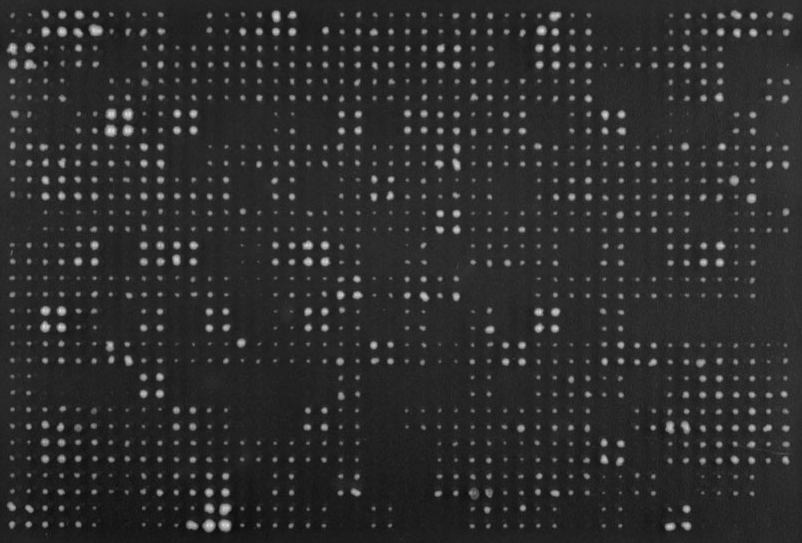

Supplement: Supplemental Material [file supp_g3.116.032607_FileS2.zip › IndividualImagesForSupplementalFile2/mel_A2-TF2-40mM3AT-after7days.1sc.png]

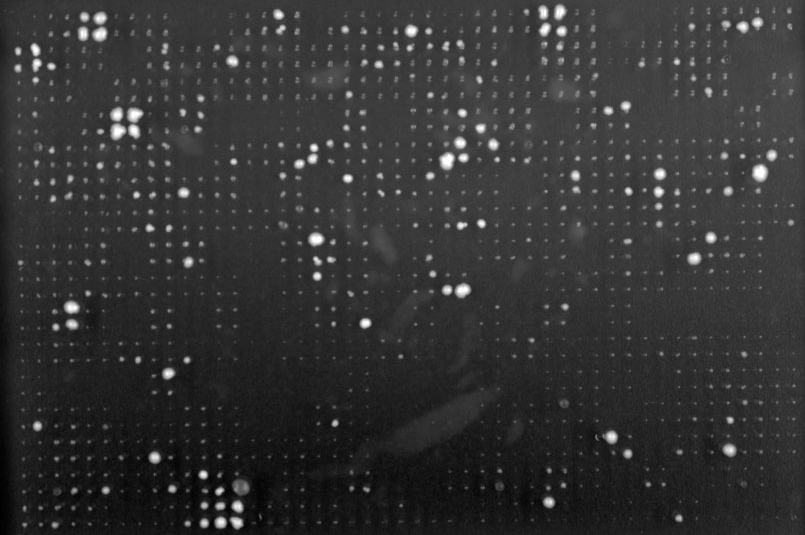

Supplement: Supplemental Material [file supp_g3.116.032607_FileS2.zip › IndividualImagesForSupplementalFile2/mel_A2-TF2-80mM3AT-after10days.png]

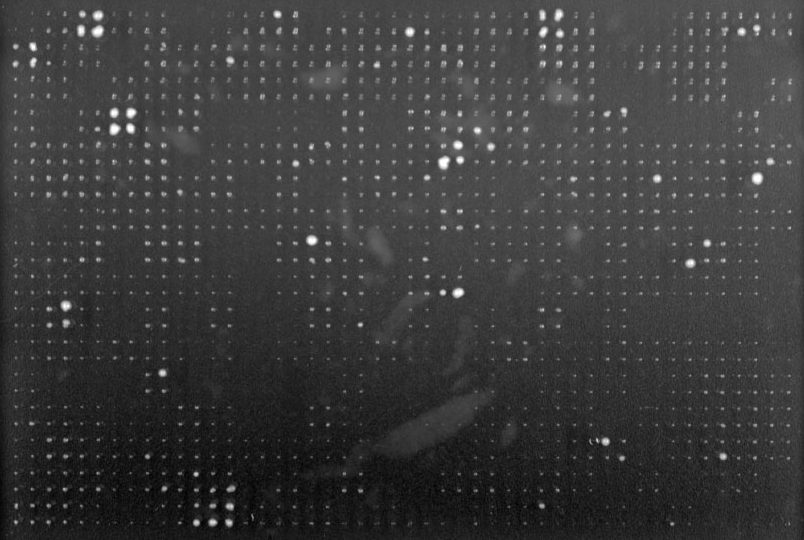

Supplement: Supplemental Material [file supp_g3.116.032607_FileS2.zip › IndividualImagesForSupplementalFile2/mel_A2-TF2-80mM3AT-after7days.1sc.png]

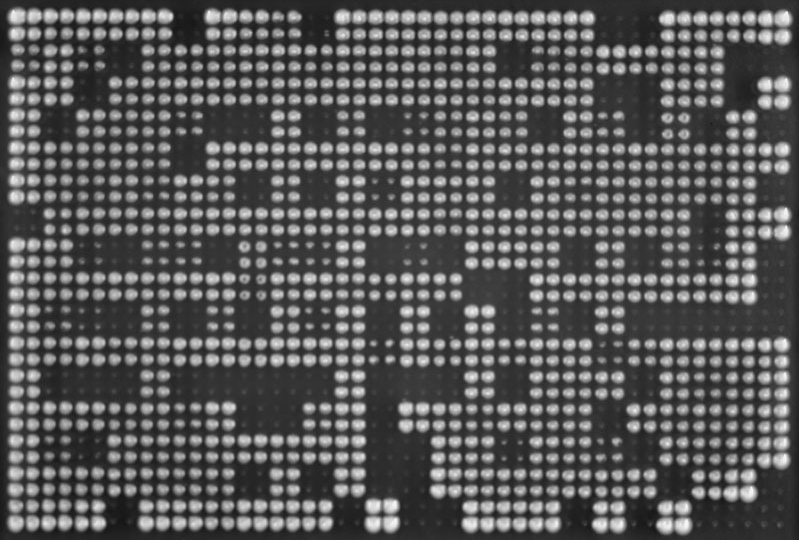

Supplement: Supplemental Material [file supp_g3.116.032607_FileS2.zip › IndividualImagesForSupplementalFile2/mel_A2-TF2-no3AT-1536-3days.1sc.png]

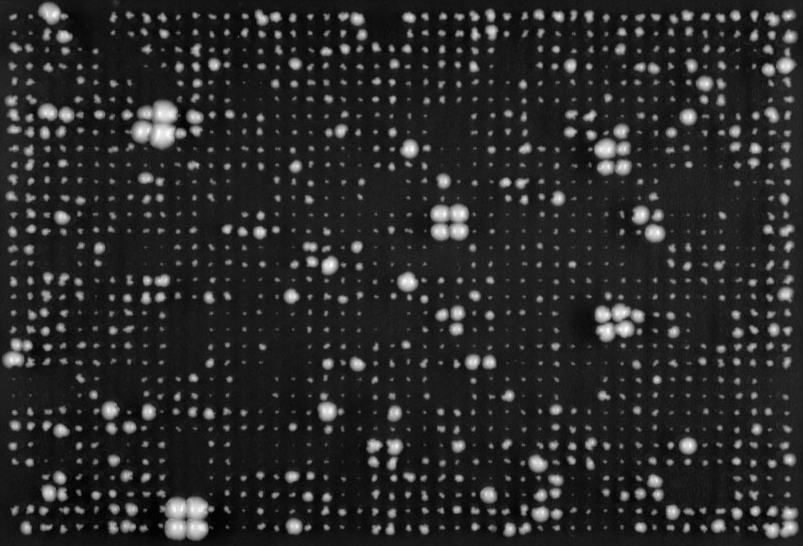

Supplement: Supplemental Material [file supp_g3.116.032607_FileS2.zip › IndividualImagesForSupplementalFile2/mel_A3-TF1-10mM3AT-after10days.png]

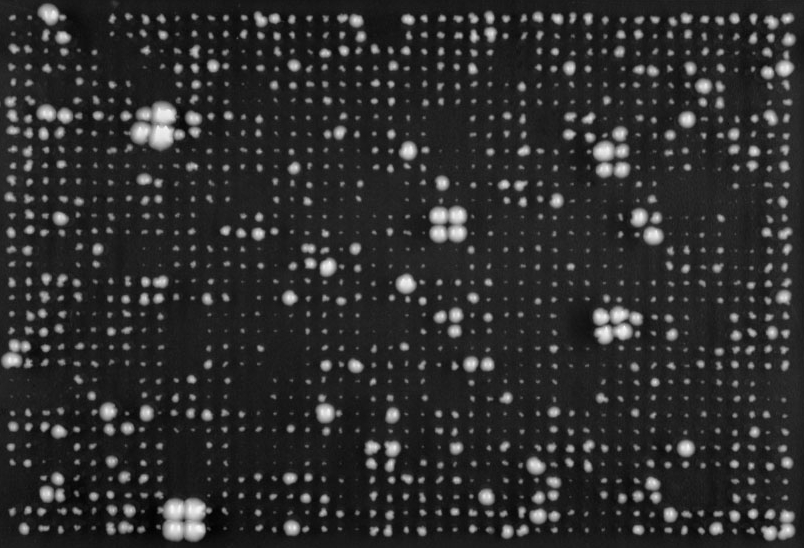

Supplement: Supplemental Material [file supp_g3.116.032607_FileS2.zip › IndividualImagesForSupplementalFile2/mel_A3-TF1-10mM3AT-after7days.1sc.png]

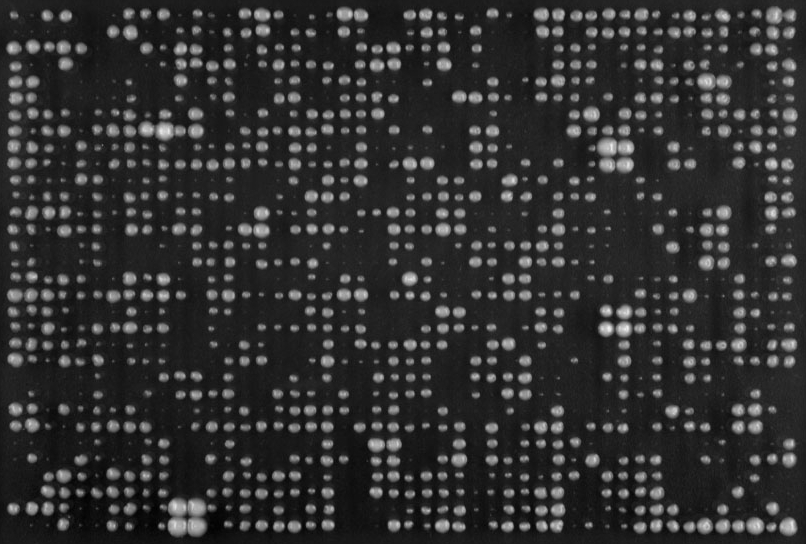

Supplement: Supplemental Material [file supp_g3.116.032607_FileS2.zip › IndividualImagesForSupplementalFile2/mel_A3-TF1-40mM3AT-after10days.png]

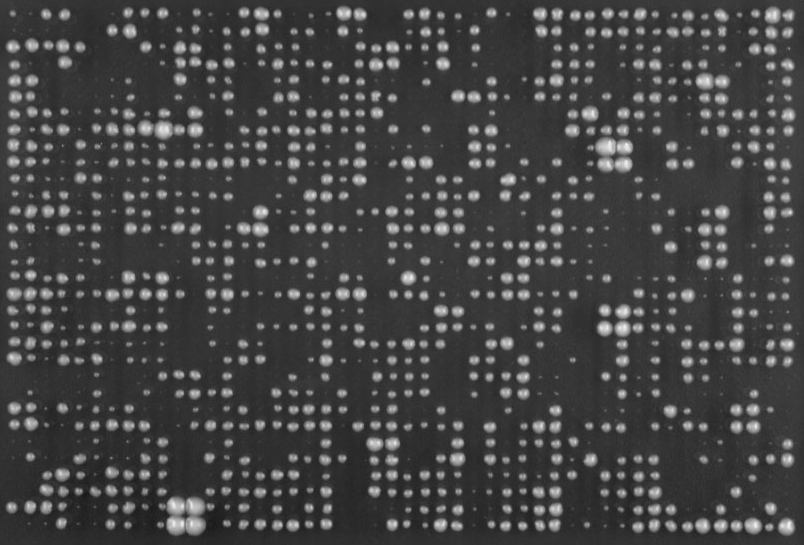

Supplement: Supplemental Material [file supp_g3.116.032607_FileS2.zip › IndividualImagesForSupplementalFile2/mel_A3-TF1-40mM3AT-after7days.1sc.png]

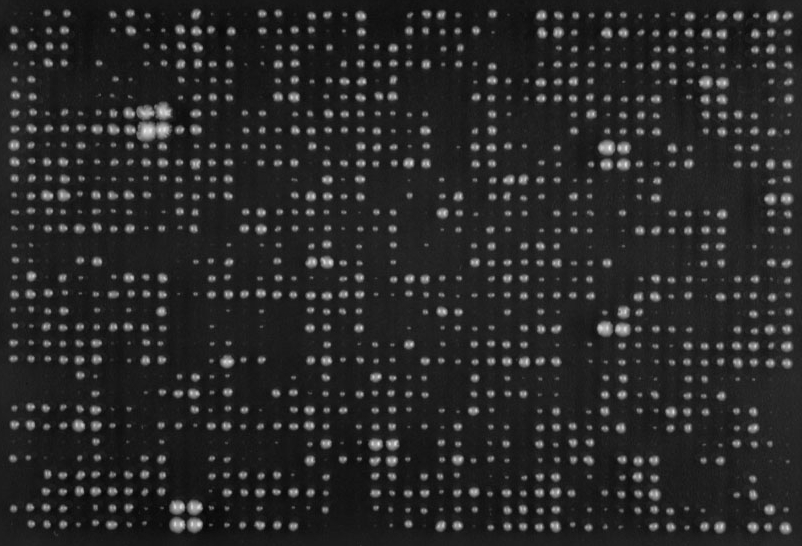

Supplement: Supplemental Material [file supp_g3.116.032607_FileS2.zip › IndividualImagesForSupplementalFile2/mel_A3-TF1-60mM3AT-after10days.png]

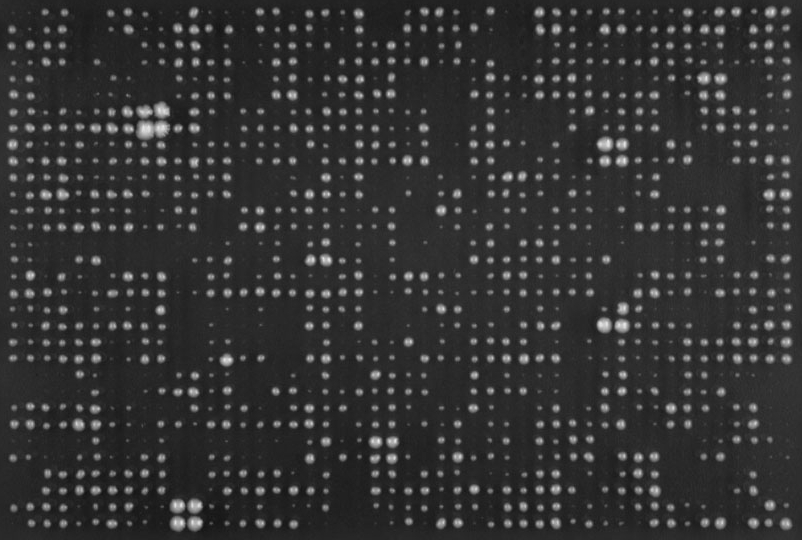

Supplement: Supplemental Material [file supp_g3.116.032607_FileS2.zip › IndividualImagesForSupplementalFile2/mel_A3-TF1-60mM3AT-after7days.1sc.png]

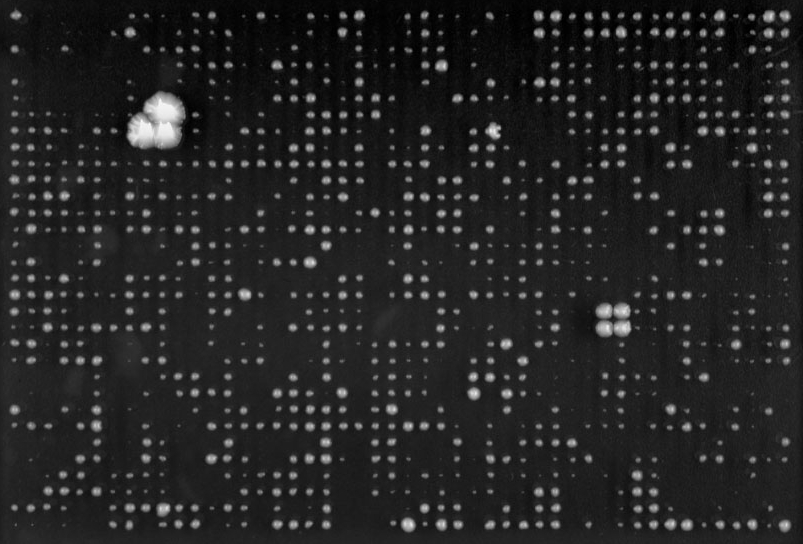

Supplement: Supplemental Material [file supp_g3.116.032607_FileS2.zip › IndividualImagesForSupplementalFile2/mel_A3-TF1-80mM3AT-after10days.png]

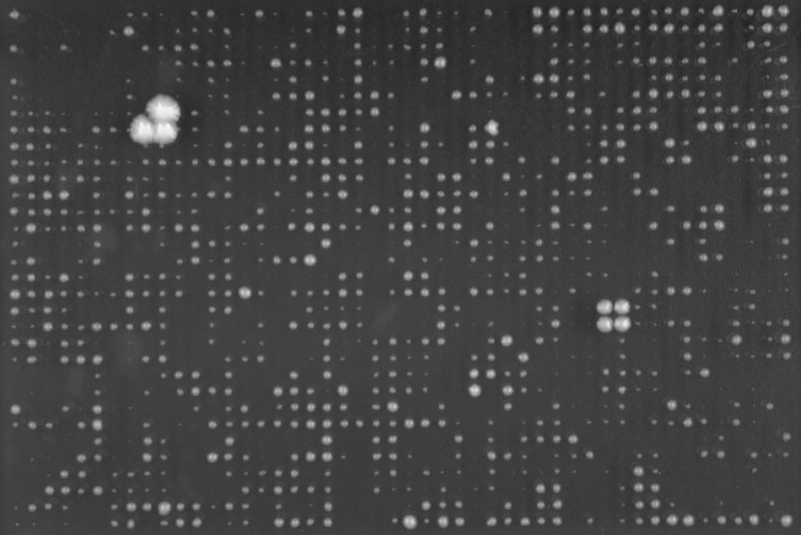

Supplement: Supplemental Material [file supp_g3.116.032607_FileS2.zip › IndividualImagesForSupplementalFile2/mel_A3-TF1-80mM3AT-after7days.1sc.png]

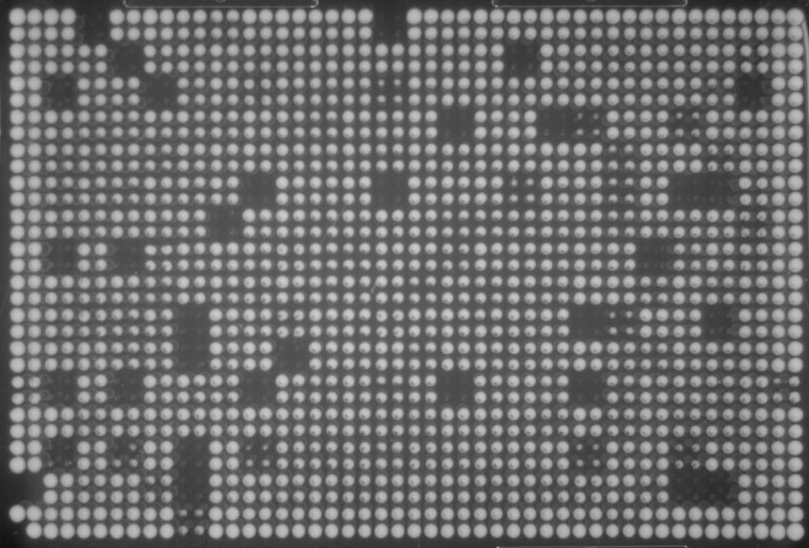

Supplement: Supplemental Material [file supp_g3.116.032607_FileS2.zip › IndividualImagesForSupplementalFile2/mel_A3-TF1-no3AT-1536-3days.1sc.png]

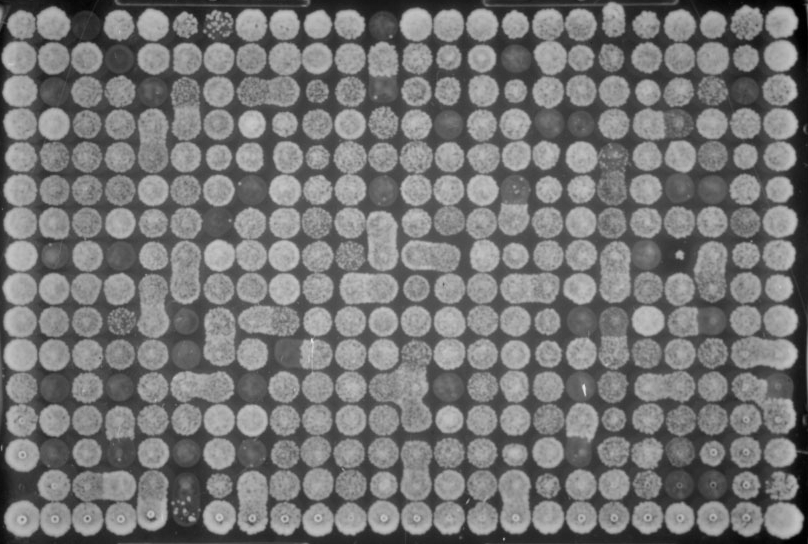

Supplement: Supplemental Material [file supp_g3.116.032607_FileS2.zip › IndividualImagesForSupplementalFile2/mel_A3-TF1-no3AT-after3days.1sc.png]

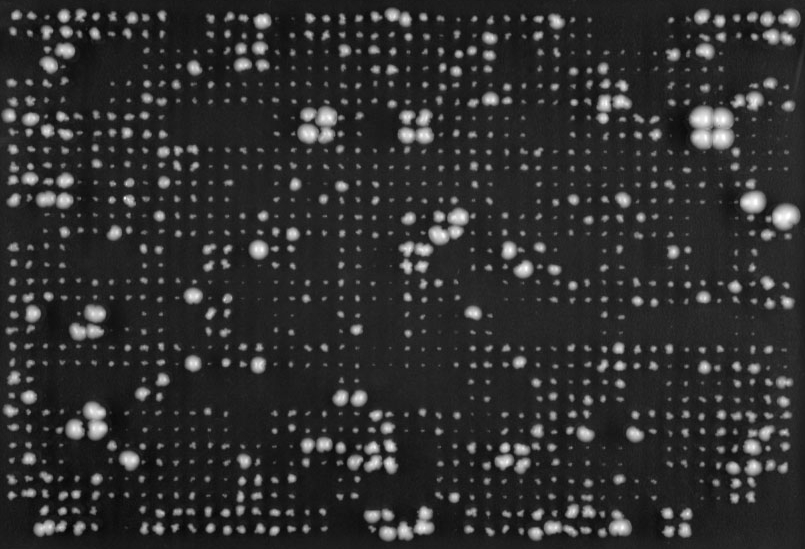

Supplement: Supplemental Material [file supp_g3.116.032607_FileS2.zip › IndividualImagesForSupplementalFile2/mel_A3-TF2-10mM3AT-after10days.png]

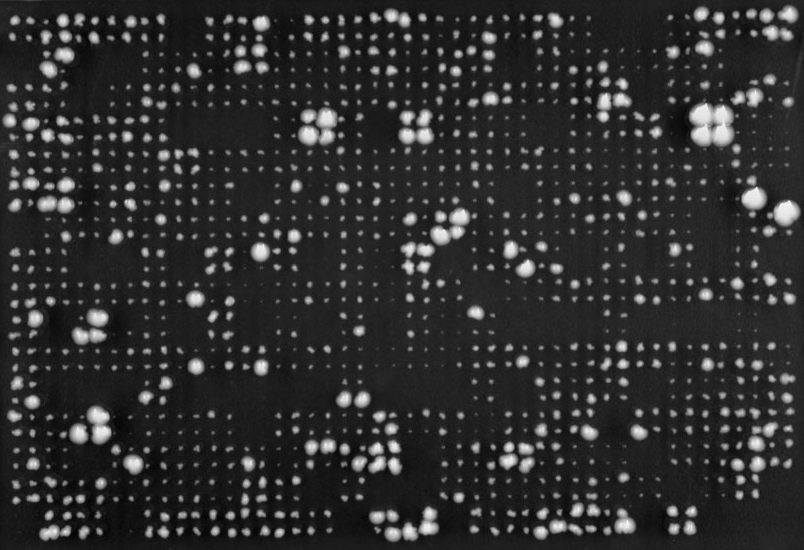

Supplement: Supplemental Material [file supp_g3.116.032607_FileS2.zip › IndividualImagesForSupplementalFile2/mel_A3-TF2-10mM3AT-after7days.1sc.png]

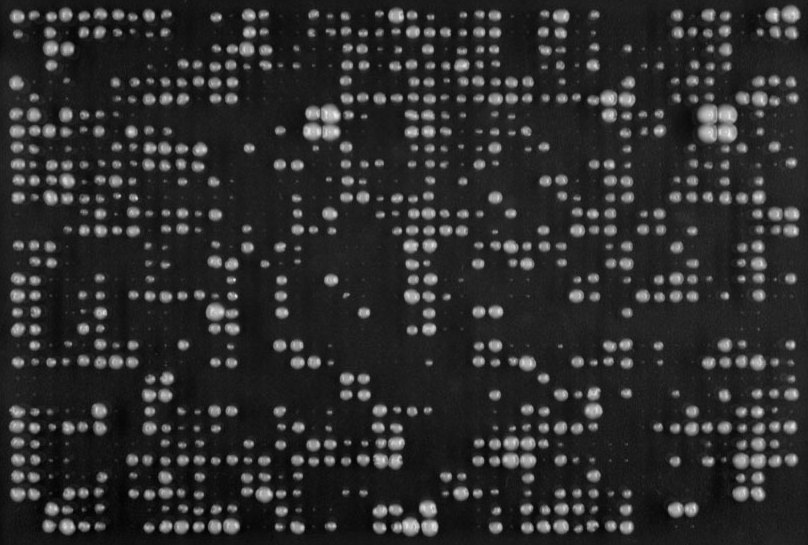

Supplement: Supplemental Material [file supp_g3.116.032607_FileS2.zip › IndividualImagesForSupplementalFile2/mel_A3-TF2-40mM3AT-after10days.png]

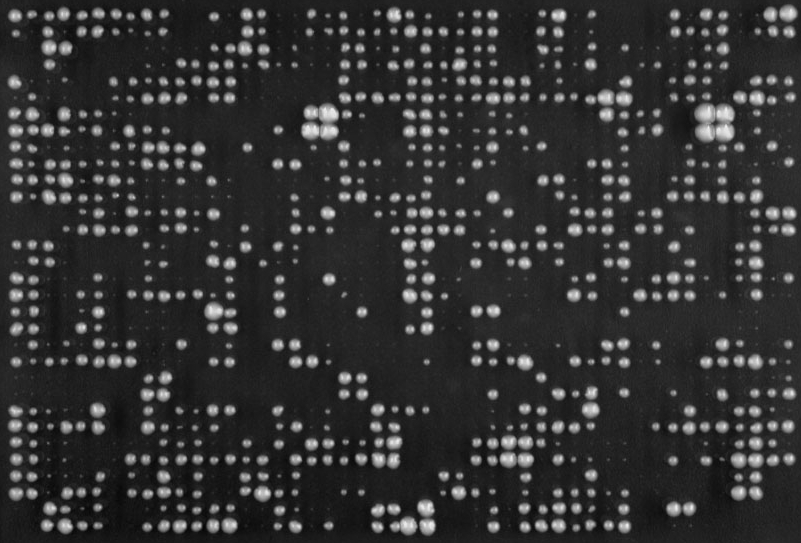

Supplement: Supplemental Material [file supp_g3.116.032607_FileS2.zip › IndividualImagesForSupplementalFile2/mel_A3-TF2-40mM3AT-after7days.1sc.png]

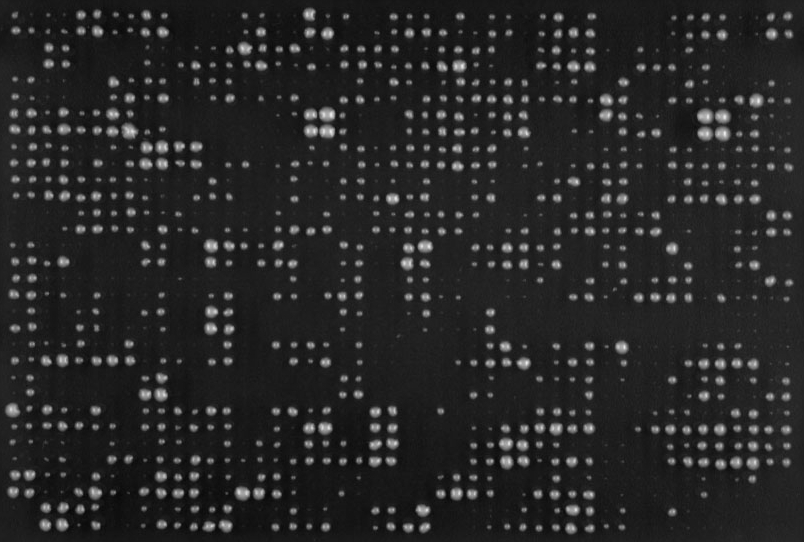

Supplement: Supplemental Material [file supp_g3.116.032607_FileS2.zip › IndividualImagesForSupplementalFile2/mel_A3-TF2-60mM3AT-after10days.png]

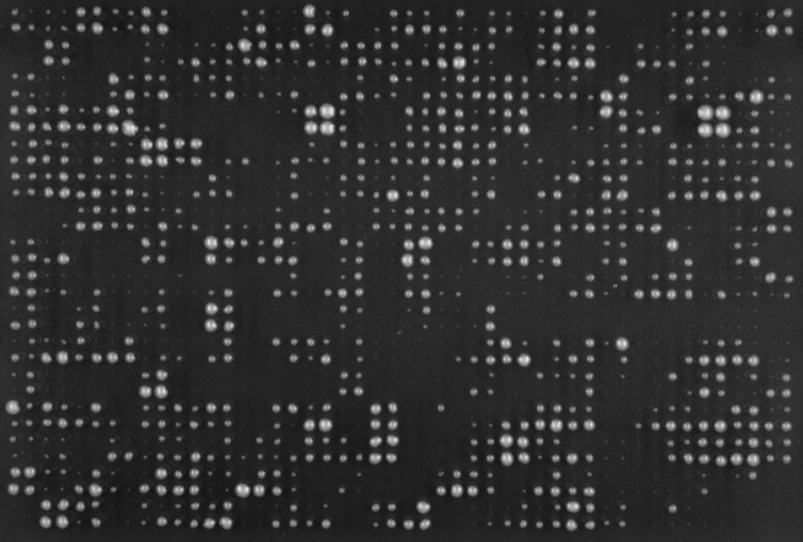

Supplement: Supplemental Material [file supp_g3.116.032607_FileS2.zip › IndividualImagesForSupplementalFile2/mel_A3-TF2-60mM3AT-after7days.1sc.png]

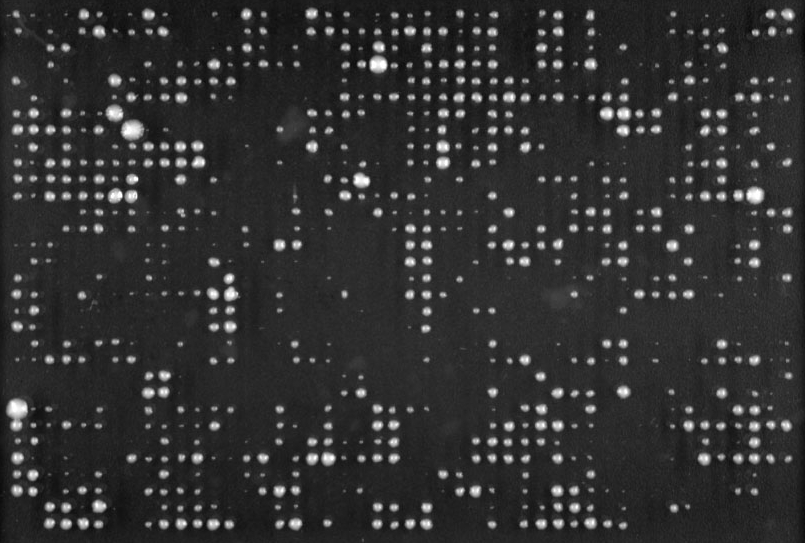

Supplement: Supplemental Material [file supp_g3.116.032607_FileS2.zip › IndividualImagesForSupplementalFile2/mel_A3-TF2-80mM3AT-after10days.png]

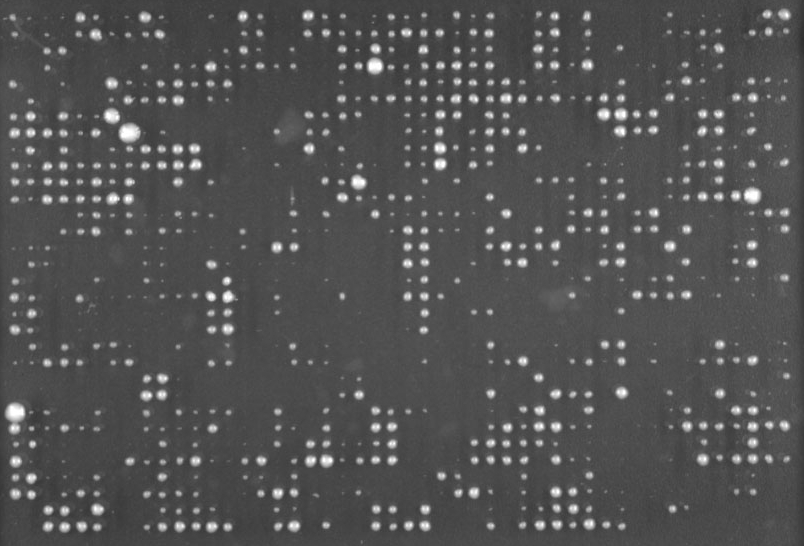

Supplement: Supplemental Material [file supp_g3.116.032607_FileS2.zip › IndividualImagesForSupplementalFile2/mel_A3-TF2-80mM3AT-after7days.1sc.png]

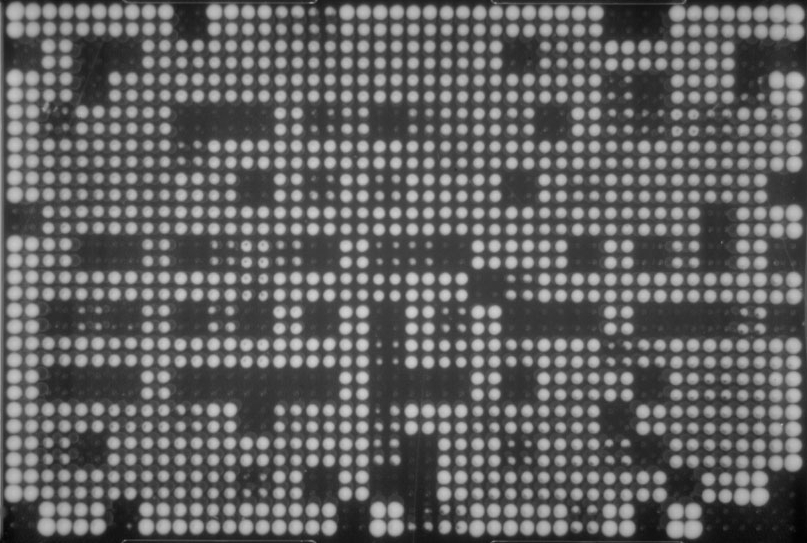

Supplement: Supplemental Material [file supp_g3.116.032607_FileS2.zip › IndividualImagesForSupplementalFile2/mel_A3-TF2-no3AT-1536-3days.1sc.png]

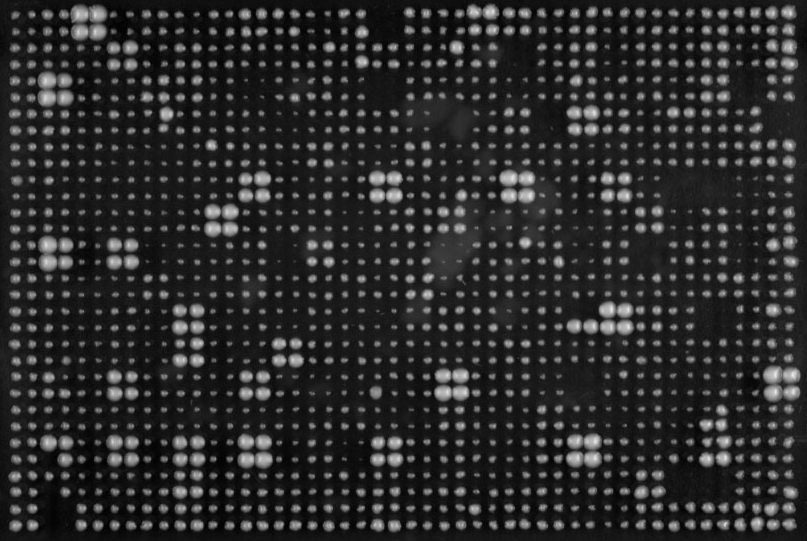

Supplement: Supplemental Material [file supp_g3.116.032607_FileS2.zip › IndividualImagesForSupplementalFile2/mel_A4-TF1-20mM3AT-after10days.png]

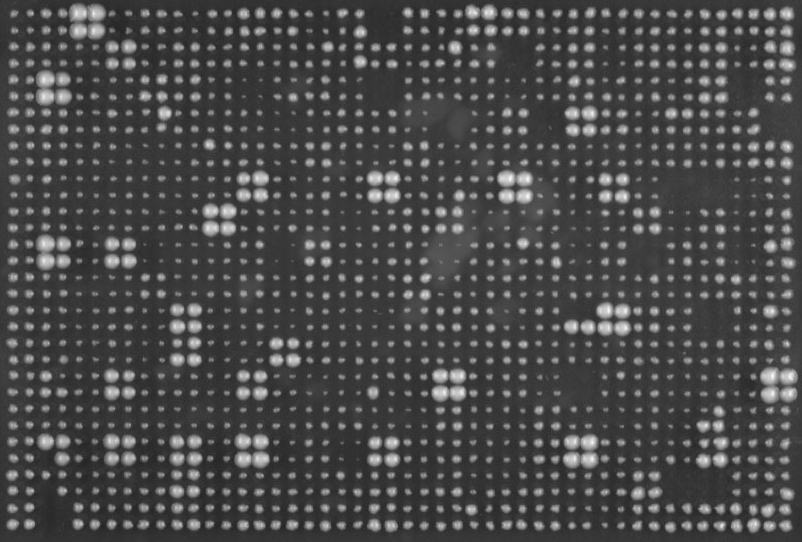

Supplement: Supplemental Material [file supp_g3.116.032607_FileS2.zip › IndividualImagesForSupplementalFile2/mel_A4-TF1-20mM3AT-after7days.1sc.png]

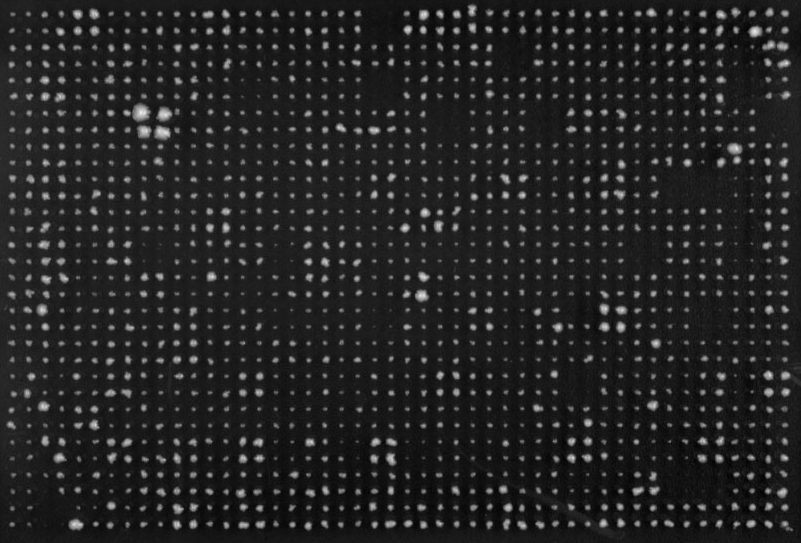

Supplement: Supplemental Material [file supp_g3.116.032607_FileS2.zip › IndividualImagesForSupplementalFile2/mel_A4-TF1-40mM3AT-after10days.png]

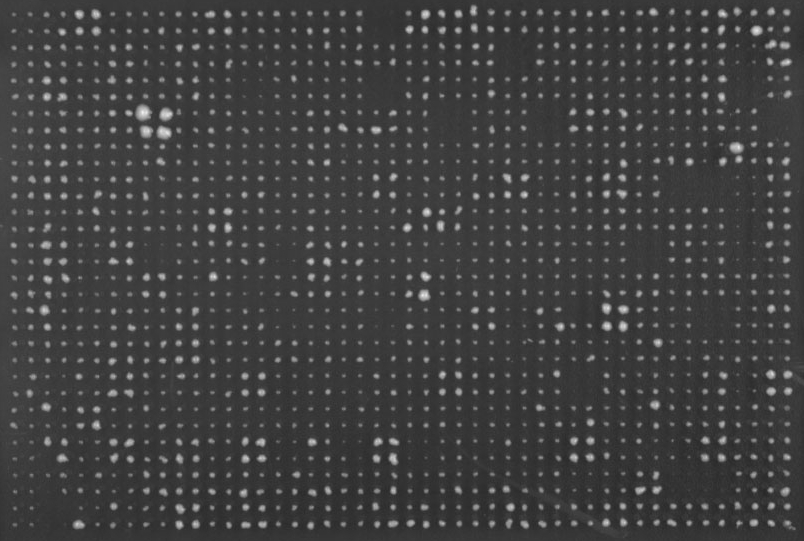

Supplement: Supplemental Material [file supp_g3.116.032607_FileS2.zip › IndividualImagesForSupplementalFile2/mel_A4-TF1-40mM3AT-after7days.1sc.png]

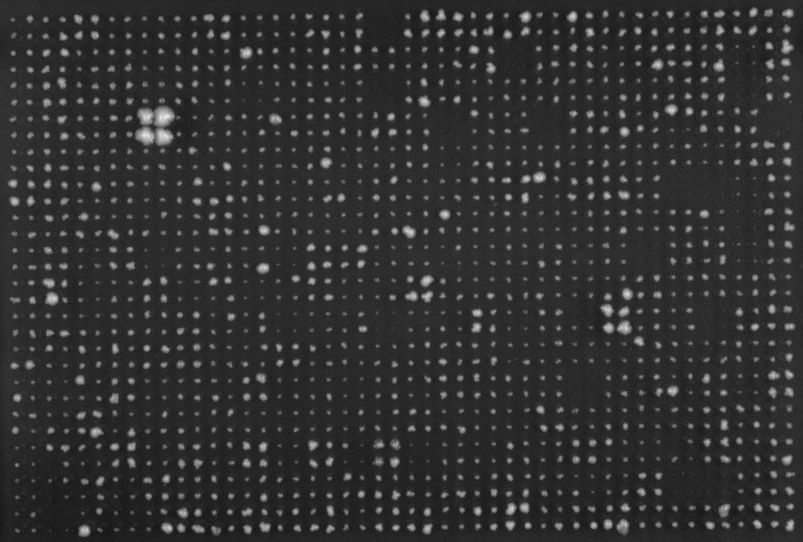

Supplement: Supplemental Material [file supp_g3.116.032607_FileS2.zip › IndividualImagesForSupplementalFile2/mel_A4-TF1-60mM3AT-after10days.png]

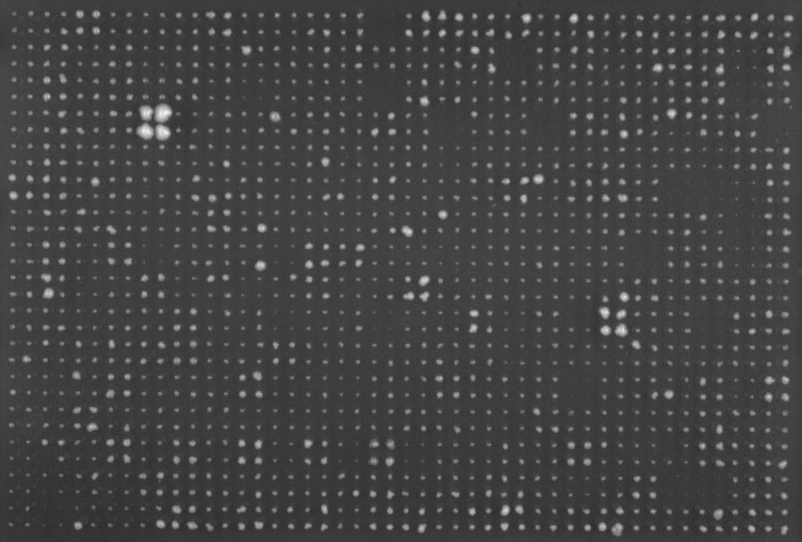

Supplement: Supplemental Material [file supp_g3.116.032607_FileS2.zip › IndividualImagesForSupplementalFile2/mel_A4-TF1-60mM3AT-after7days.1sc.png]

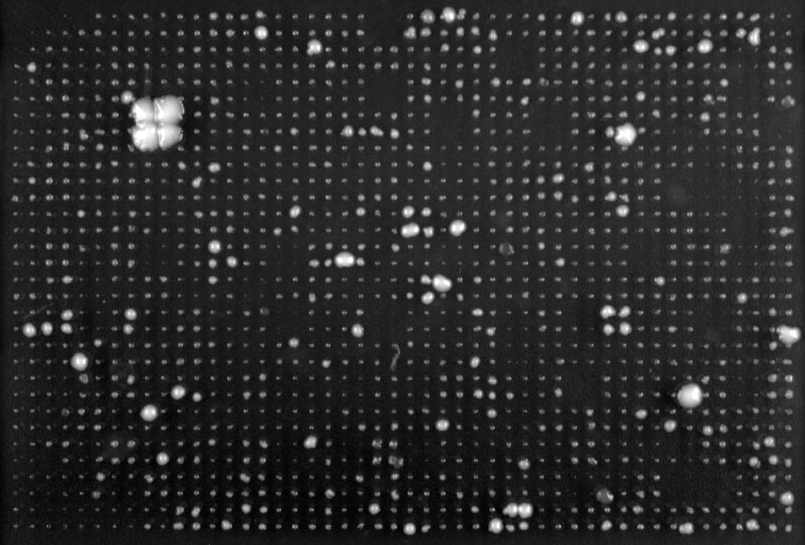

Supplement: Supplemental Material [file supp_g3.116.032607_FileS2.zip › IndividualImagesForSupplementalFile2/mel_A4-TF1-80mM3AT-after10days.png]

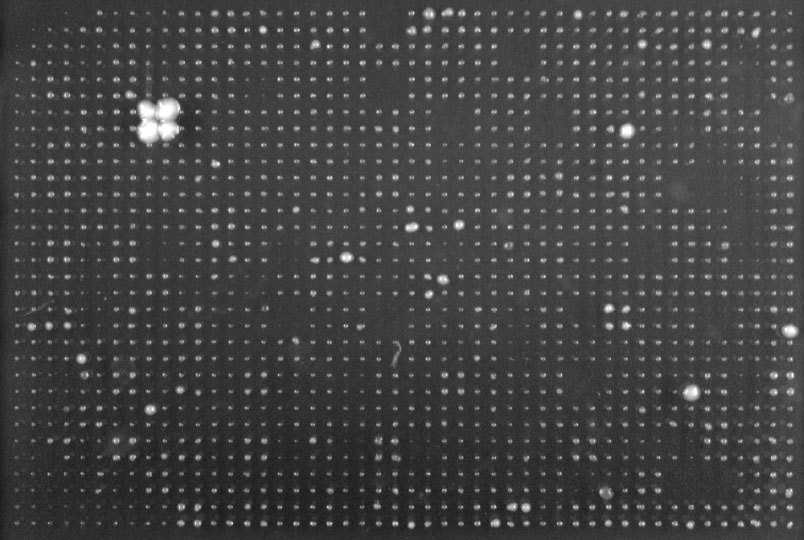

Supplement: Supplemental Material [file supp_g3.116.032607_FileS2.zip › IndividualImagesForSupplementalFile2/mel_A4-TF1-80mM3AT-after7days.1sc.png]

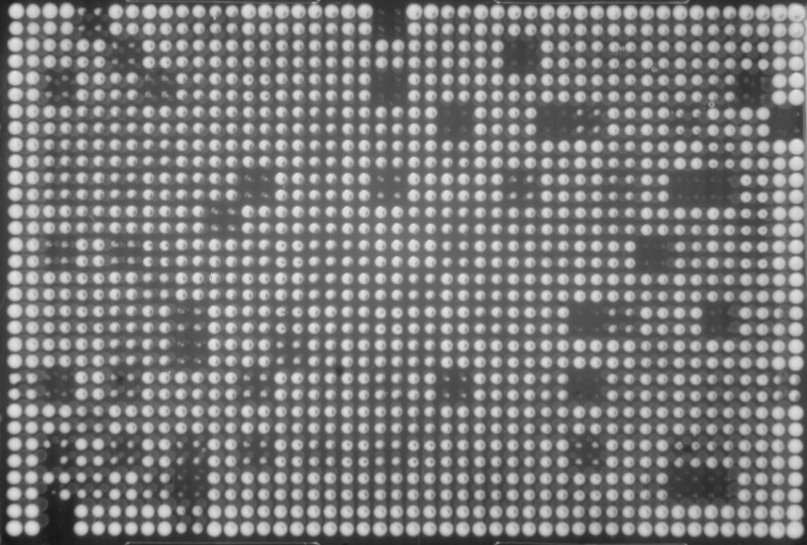

Supplement: Supplemental Material [file supp_g3.116.032607_FileS2.zip › IndividualImagesForSupplementalFile2/mel_A4-TF1-no3AT-1536-3days.1sc.png]

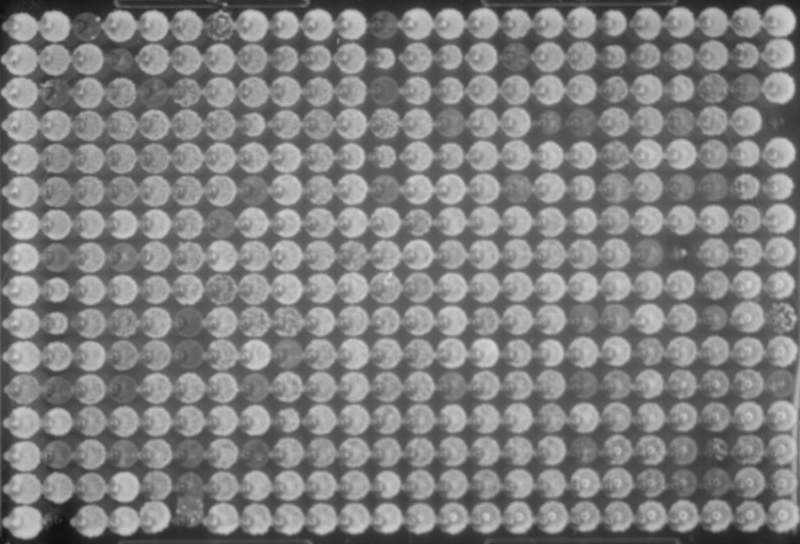

Supplement: Supplemental Material [file supp_g3.116.032607_FileS2.zip › IndividualImagesForSupplementalFile2/mel_A4-TF1-no3AT-after3days.1sc.png]

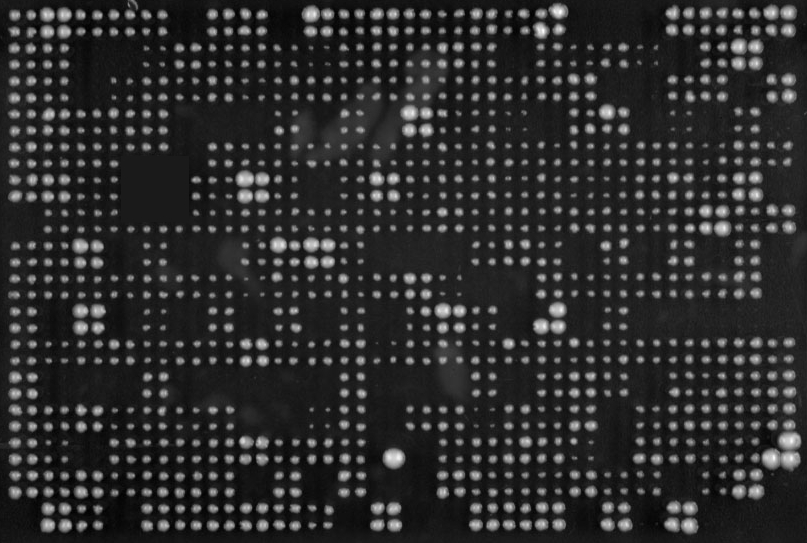

Supplement: Supplemental Material [file supp_g3.116.032607_FileS2.zip › IndividualImagesForSupplementalFile2/mel_A4-TF2-20mM3AT-after10days.png]

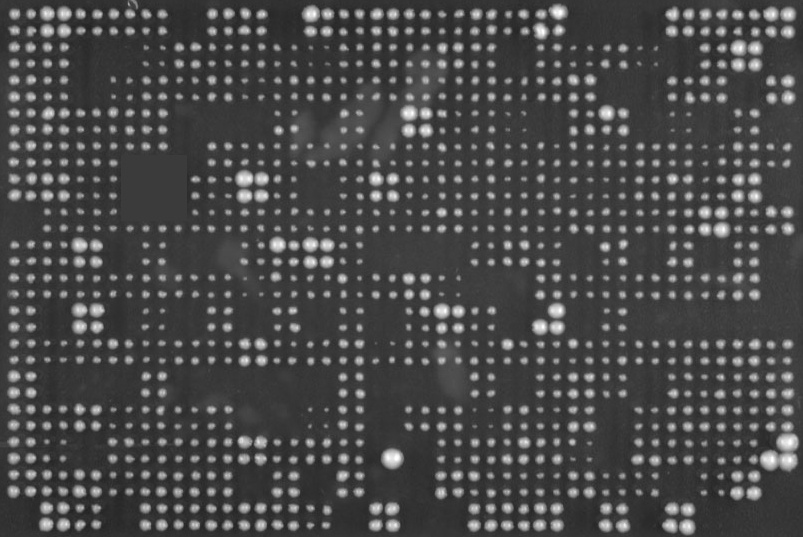

Supplement: Supplemental Material [file supp_g3.116.032607_FileS2.zip › IndividualImagesForSupplementalFile2/mel_A4-TF2-20mM3AT-after7days.1sc.png]

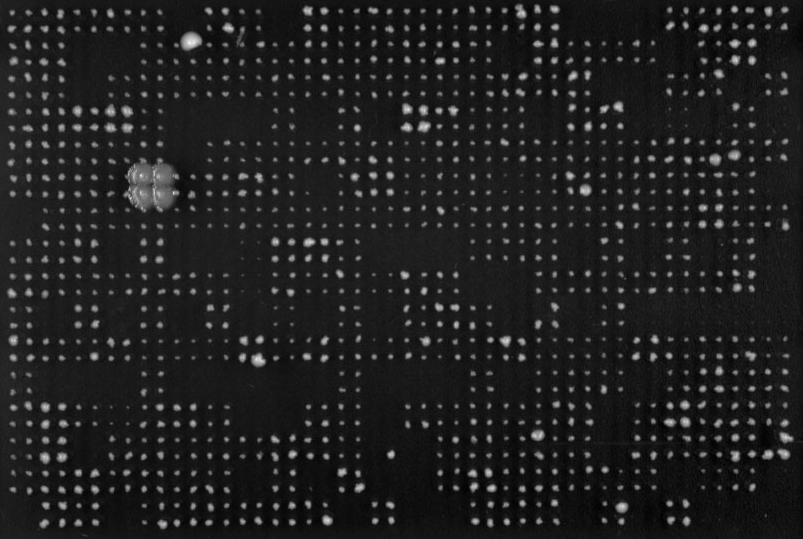

Supplement: Supplemental Material [file supp_g3.116.032607_FileS2.zip › IndividualImagesForSupplementalFile2/mel_A4-TF2-40mM3AT-after10days.png]

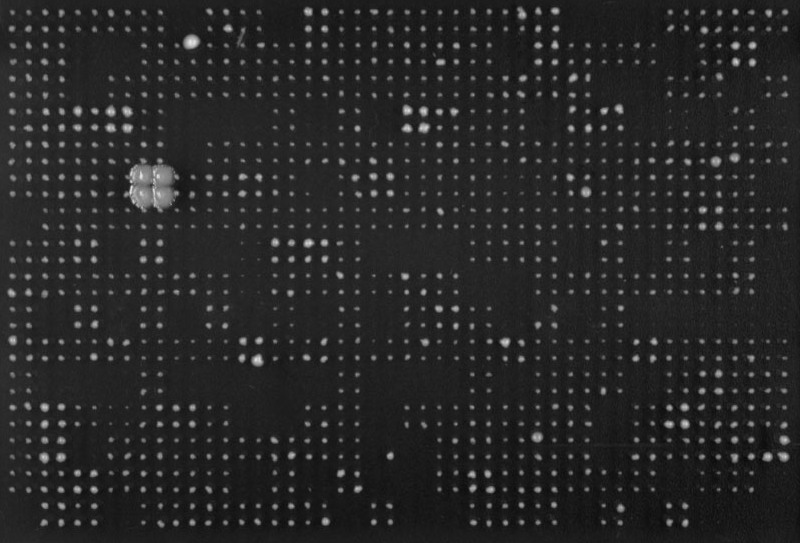

Supplement: Supplemental Material [file supp_g3.116.032607_FileS2.zip › IndividualImagesForSupplementalFile2/mel_A4-TF2-40mM3AT-after7days.1sc.png]

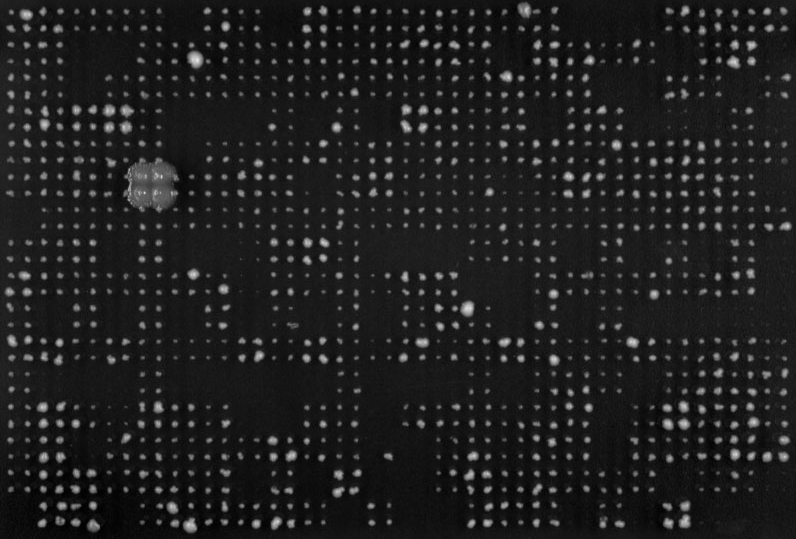

Supplement: Supplemental Material [file supp_g3.116.032607_FileS2.zip › IndividualImagesForSupplementalFile2/mel_A4-TF2-60mM3AT-after10days.png]

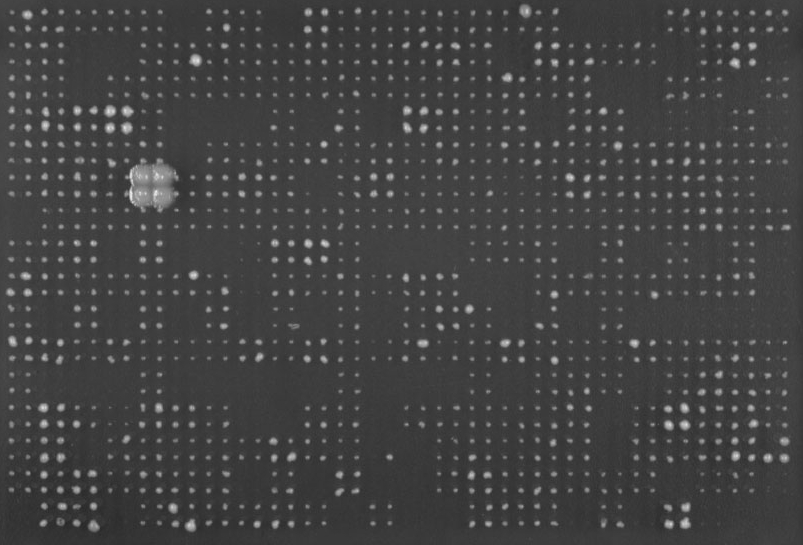

Supplement: Supplemental Material [file supp_g3.116.032607_FileS2.zip › IndividualImagesForSupplementalFile2/mel_A4-TF2-60mM3AT-after7days.1sc.png]

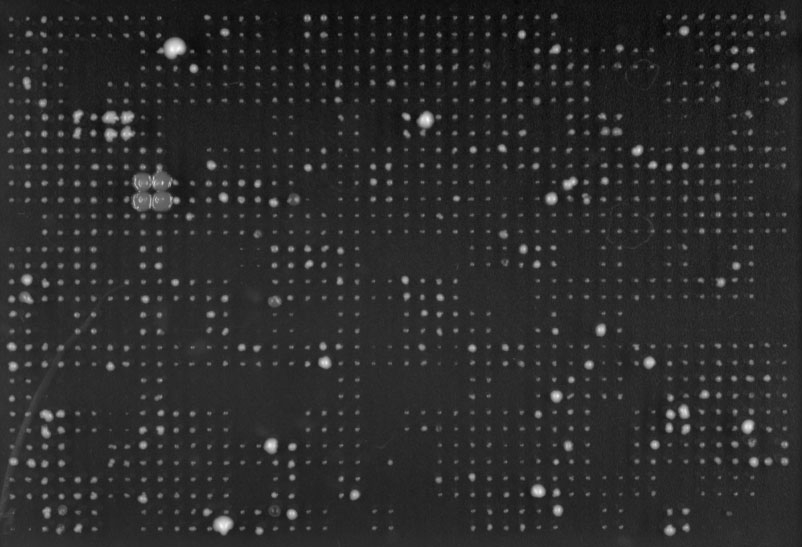

Supplement: Supplemental Material [file supp_g3.116.032607_FileS2.zip › IndividualImagesForSupplementalFile2/mel_A4-TF2-80mM3AT-after10days.png]

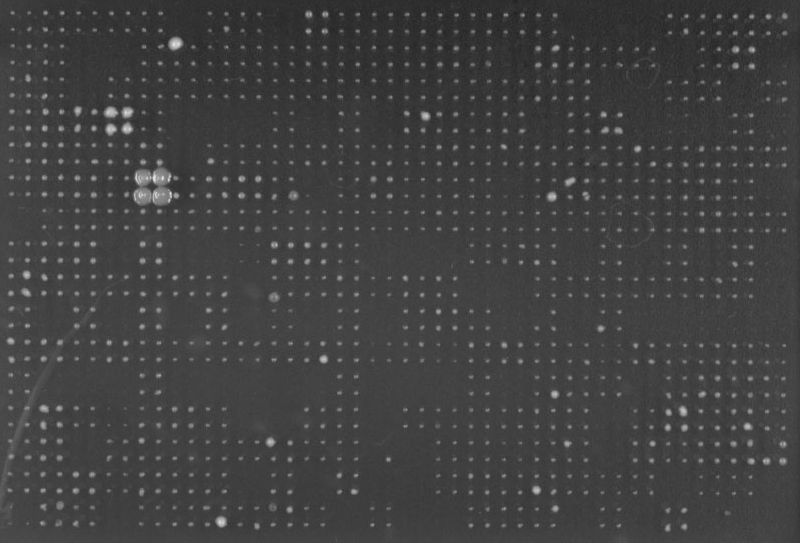

Supplement: Supplemental Material [file supp_g3.116.032607_FileS2.zip › IndividualImagesForSupplementalFile2/mel_A4-TF2-80mM3AT-after7days.1sc.png]

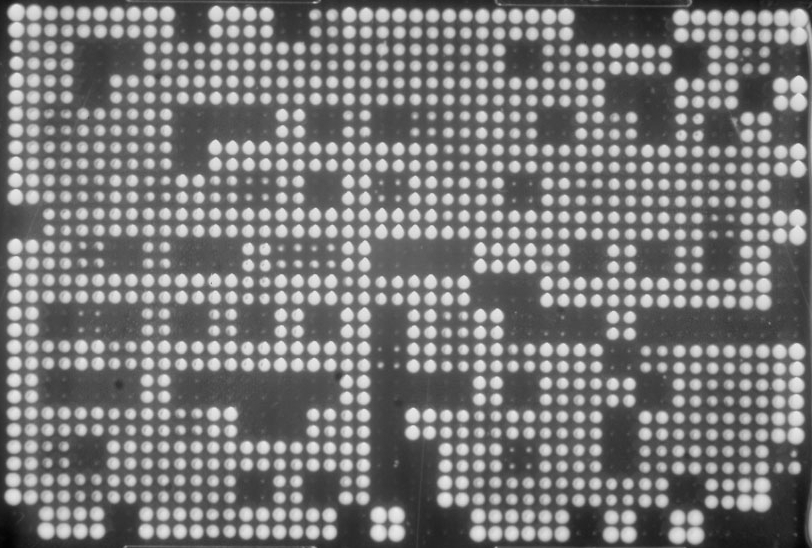

Supplement: Supplemental Material [file supp_g3.116.032607_FileS2.zip › IndividualImagesForSupplementalFile2/mel_A4-TF2-no3AT-1536-3days.1sc.png]

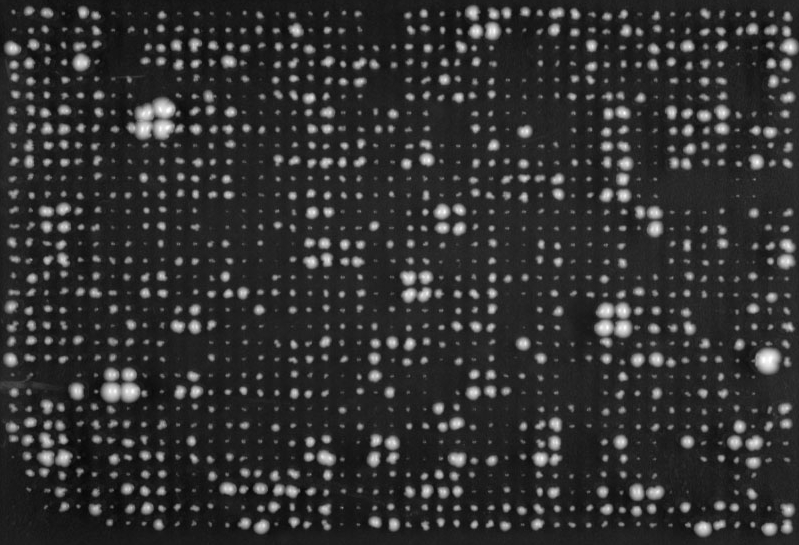

Supplement: Supplemental Material [file supp_g3.116.032607_FileS2.zip › IndividualImagesForSupplementalFile2/mel_A5-TF1-10mM3AT-after10days.png]

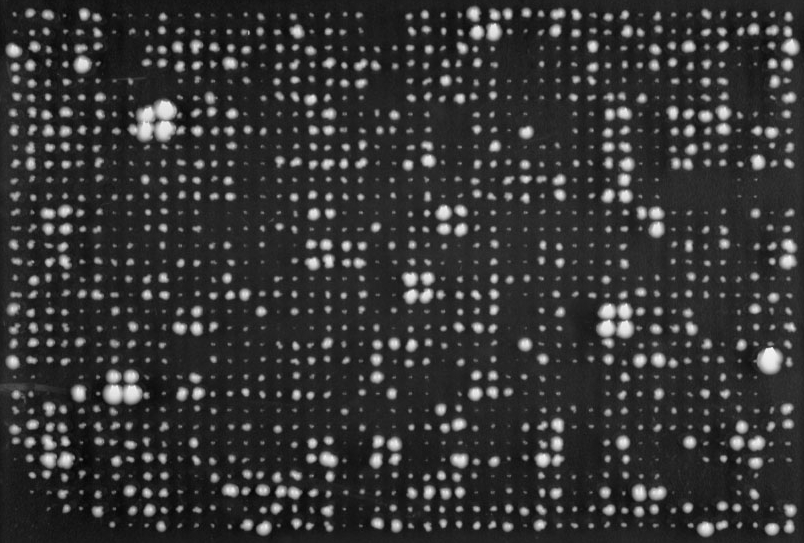

Supplement: Supplemental Material [file supp_g3.116.032607_FileS2.zip › IndividualImagesForSupplementalFile2/mel_A5-TF1-10mM3AT-after7days.1sc.png]

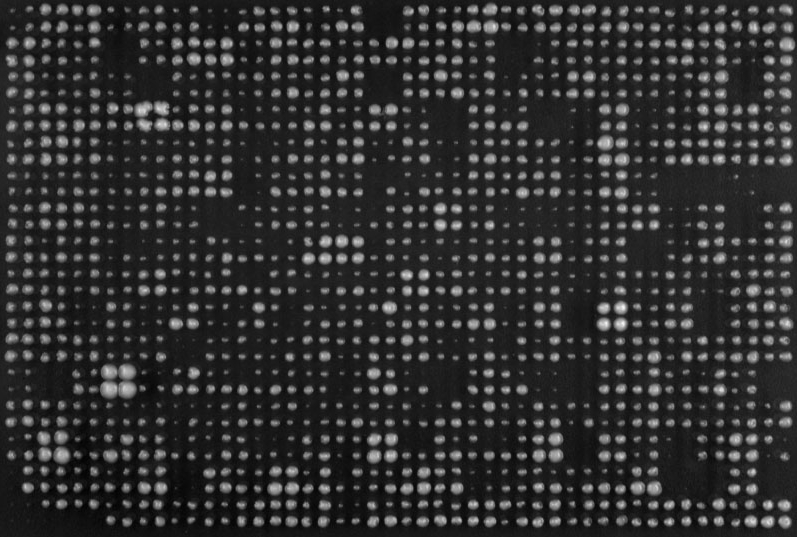

Supplement: Supplemental Material [file supp_g3.116.032607_FileS2.zip › IndividualImagesForSupplementalFile2/mel_A5-TF1-40mM3AT-after10days.png]

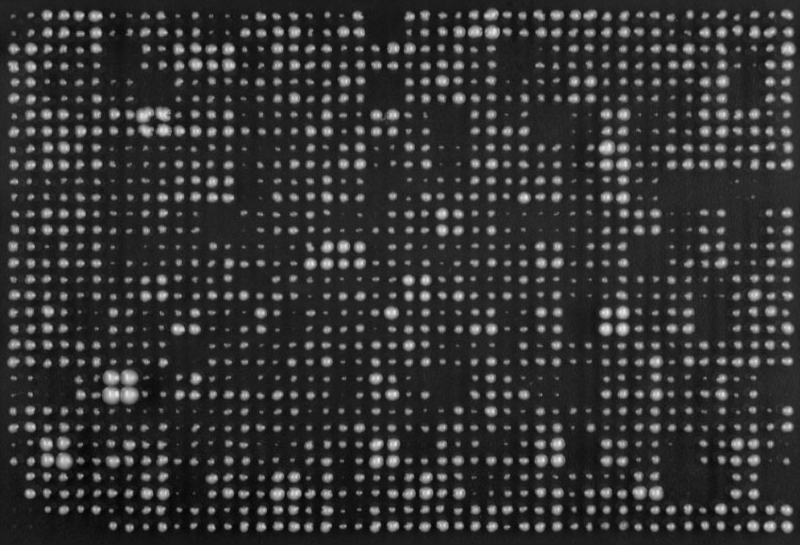

Supplement: Supplemental Material [file supp_g3.116.032607_FileS2.zip › IndividualImagesForSupplementalFile2/mel_A5-TF1-40mM3AT-after7days.1sc.png]

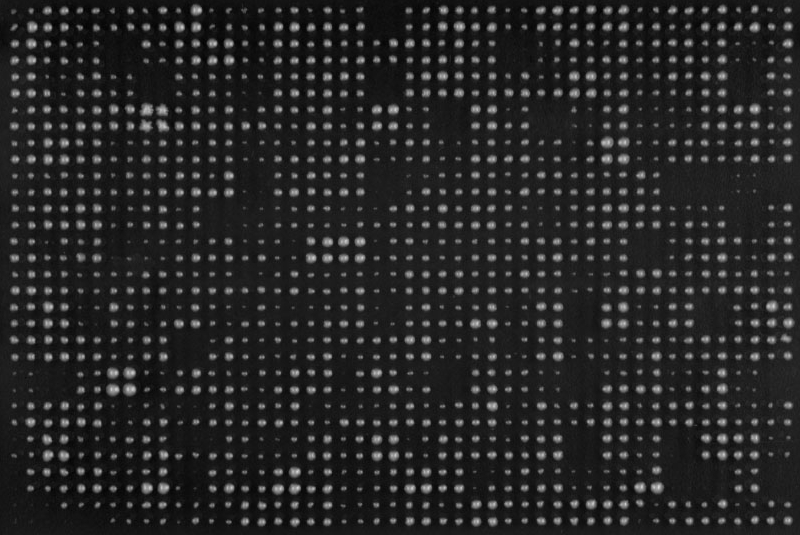

Supplement: Supplemental Material [file supp_g3.116.032607_FileS2.zip › IndividualImagesForSupplementalFile2/mel_A5-TF1-60mM3AT-after10days.png]

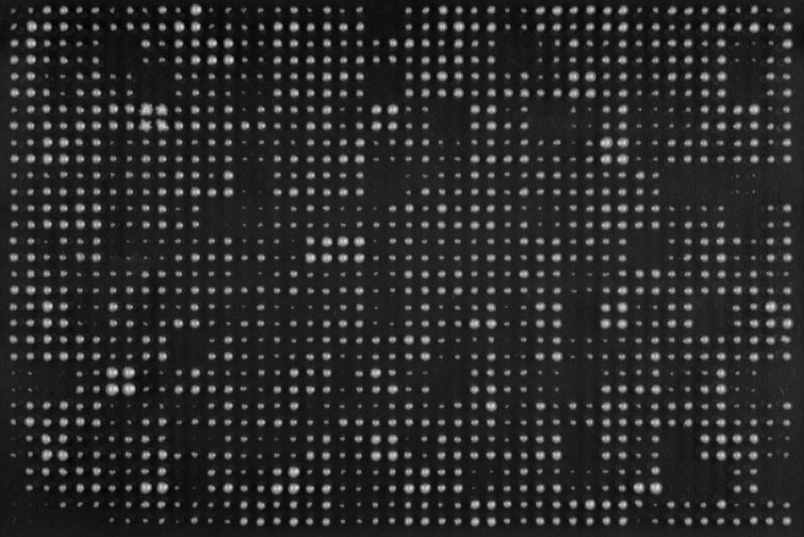

Supplement: Supplemental Material [file supp_g3.116.032607_FileS2.zip › IndividualImagesForSupplementalFile2/mel_A5-TF1-60mM3AT-after7days.1sc.png]

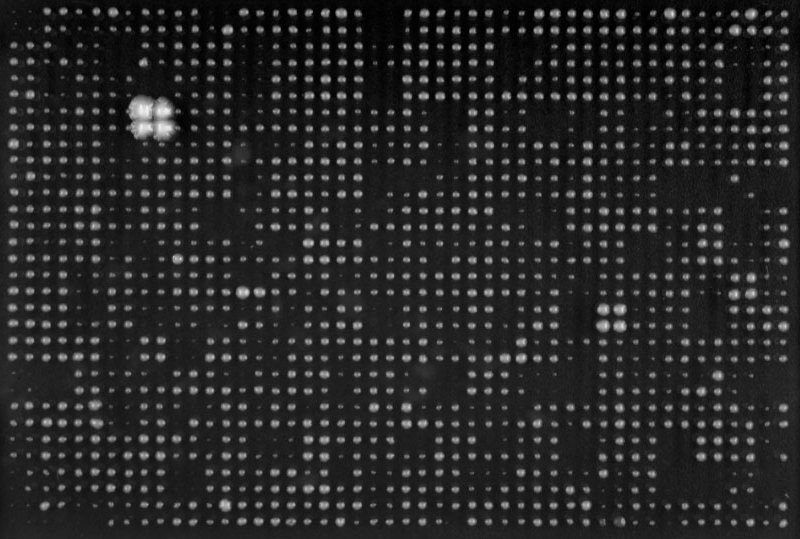

Supplement: Supplemental Material [file supp_g3.116.032607_FileS2.zip › IndividualImagesForSupplementalFile2/mel_A5-TF1-80mM3AT-after10days.png]

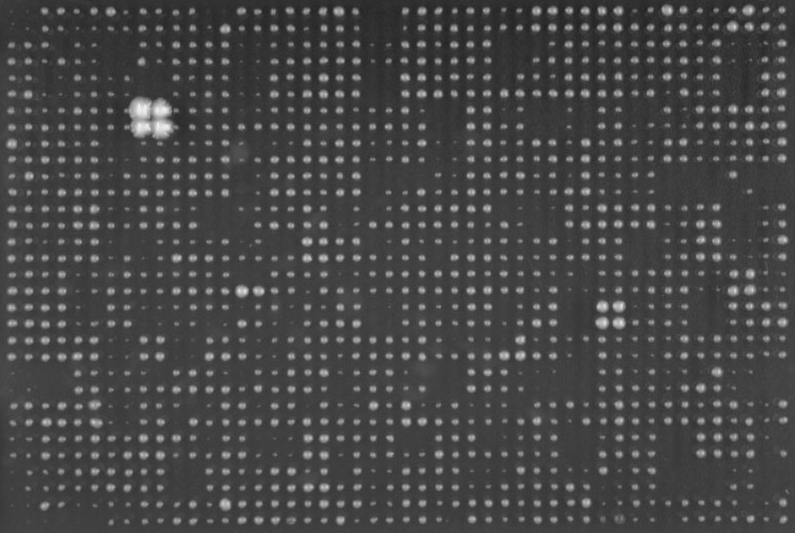

Supplement: Supplemental Material [file supp_g3.116.032607_FileS2.zip › IndividualImagesForSupplementalFile2/mel_A5-TF1-80mM3AT-after7days.1sc.png]

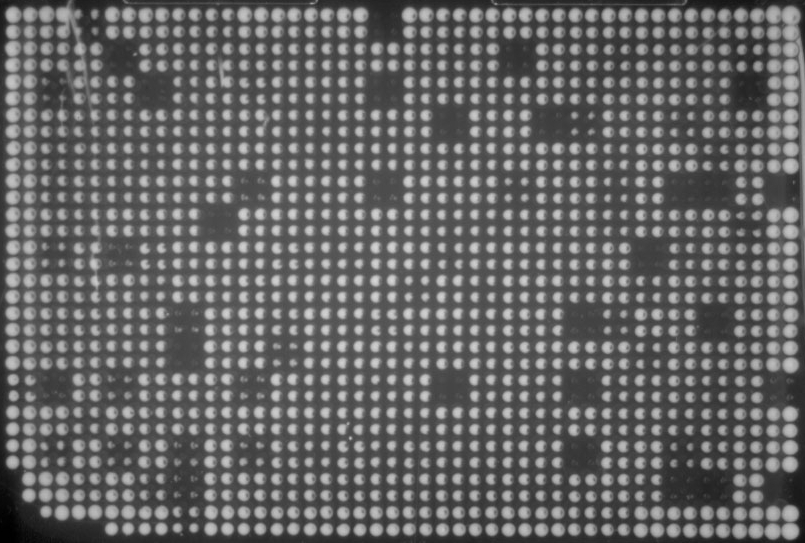

Supplement: Supplemental Material [file supp_g3.116.032607_FileS2.zip › IndividualImagesForSupplementalFile2/mel_A5-TF1-no3AT-1536-3days.1sc.png]

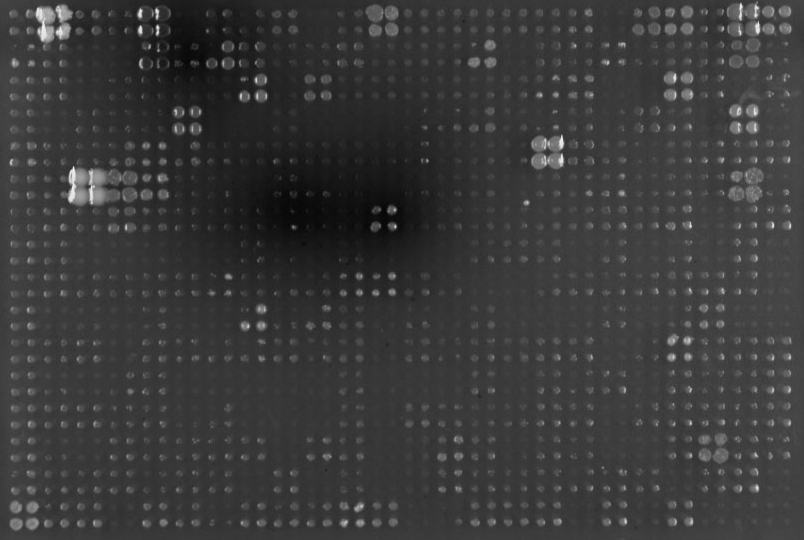

Supplement: Supplemental Material [file supp_g3.116.032607_FileS2.zip › IndividualImagesForSupplementalFile2/mel_A5-TF2-10mM3AT-after9days.1sc.png]

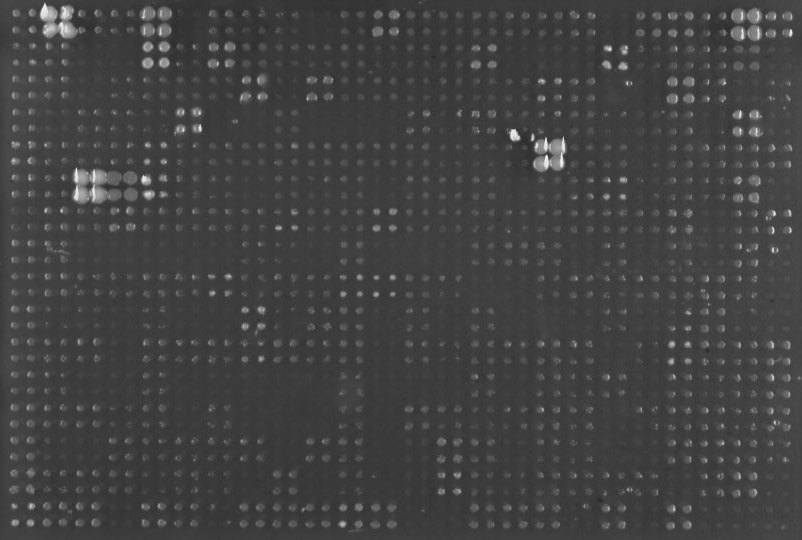

Supplement: Supplemental Material [file supp_g3.116.032607_FileS2.zip › IndividualImagesForSupplementalFile2/mel_A5-TF2-20mM3AT-after9days.1sc.png]

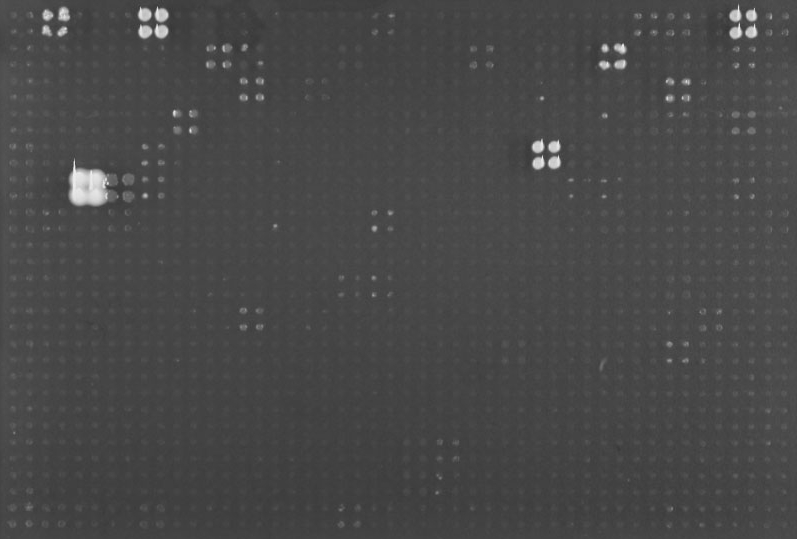

Supplement: Supplemental Material [file supp_g3.116.032607_FileS2.zip › IndividualImagesForSupplementalFile2/mel_A5-TF2-40mM3AT-after9days.1sc.png]

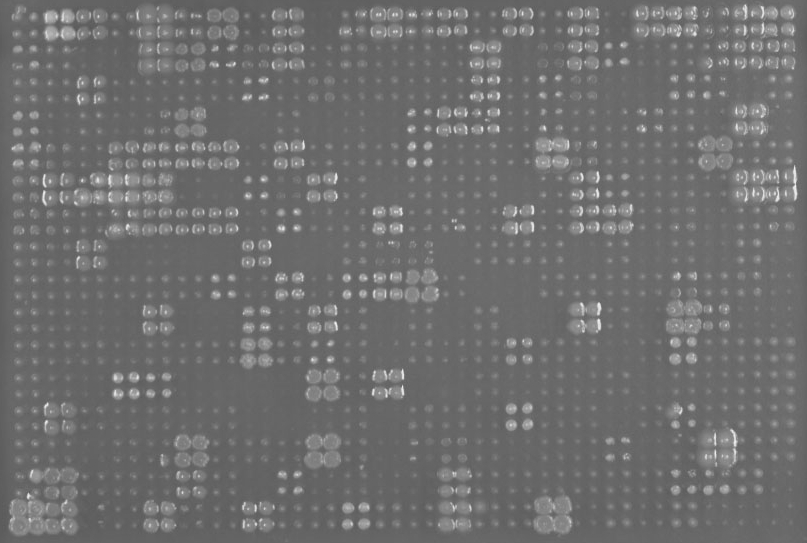

Supplement: Supplemental Material [file supp_g3.116.032607_FileS2.zip › IndividualImagesForSupplementalFile2/mel_A5-TF2-no3AT-1536-6dycnt.1sc.png]

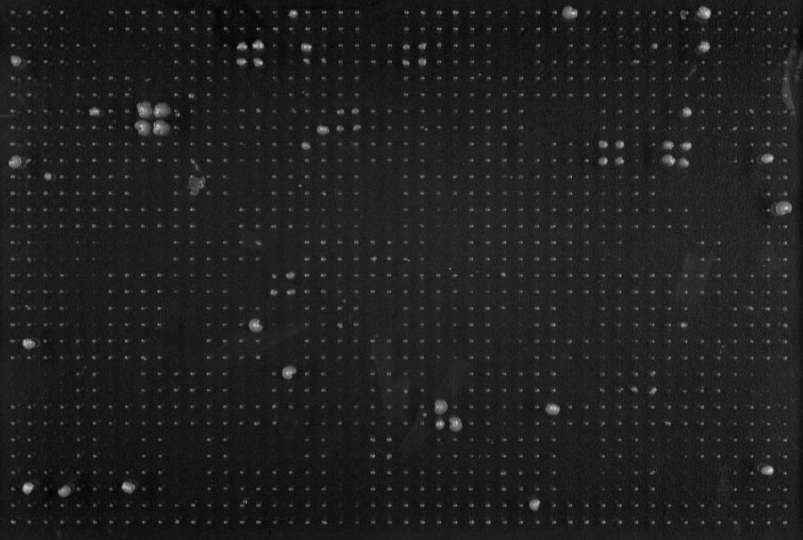

Supplement: Supplemental Material [file supp_g3.116.032607_FileS2.zip › IndividualImagesForSupplementalFile2/mel_A6-TF1-10mM3AT-after10days.png]

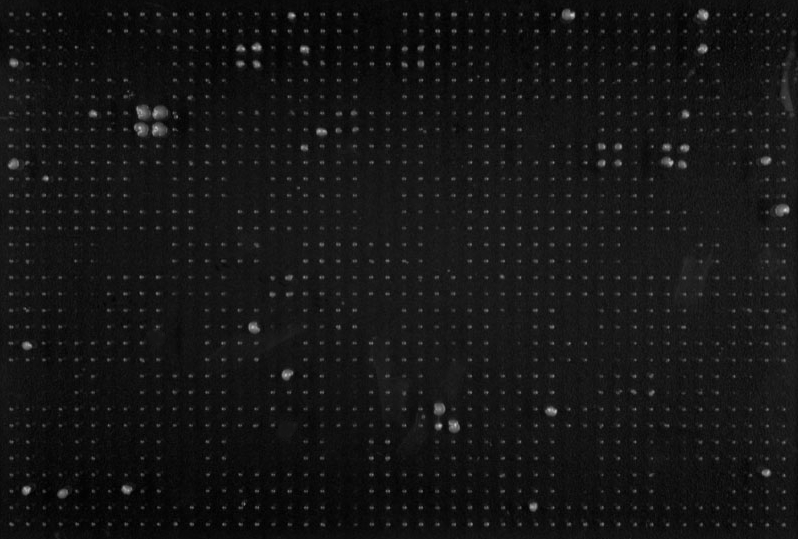

Supplement: Supplemental Material [file supp_g3.116.032607_FileS2.zip › IndividualImagesForSupplementalFile2/mel_A6-TF1-10mM3AT-after7days.1sc.png]

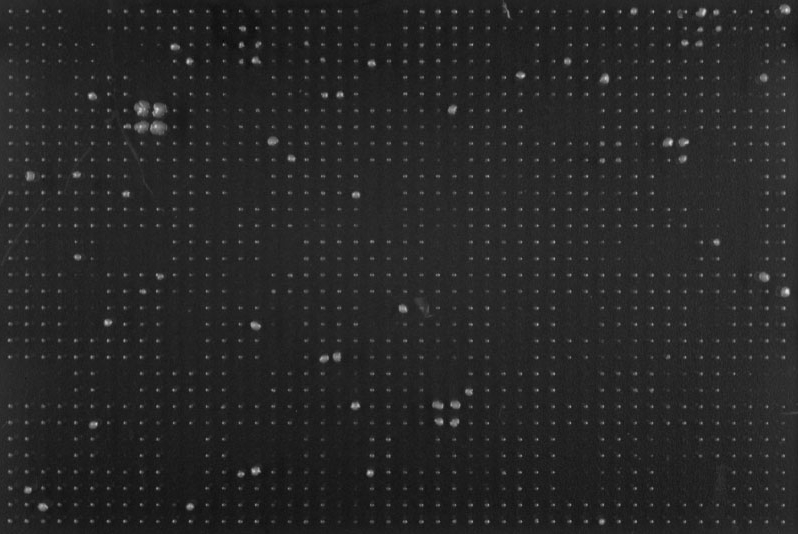

Supplement: Supplemental Material [file supp_g3.116.032607_FileS2.zip › IndividualImagesForSupplementalFile2/mel_A6-TF1-20mM3AT-after10days.png]

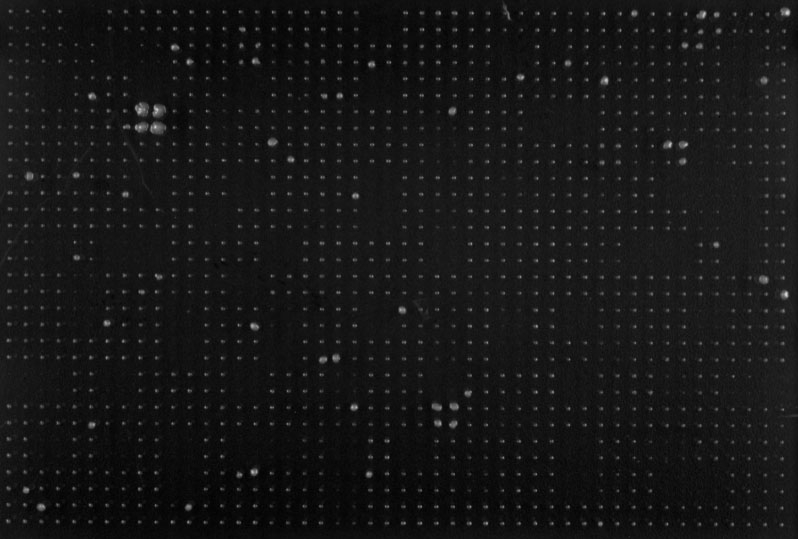

Supplement: Supplemental Material [file supp_g3.116.032607_FileS2.zip › IndividualImagesForSupplementalFile2/mel_A6-TF1-20mM3AT-after7days.1sc.png]

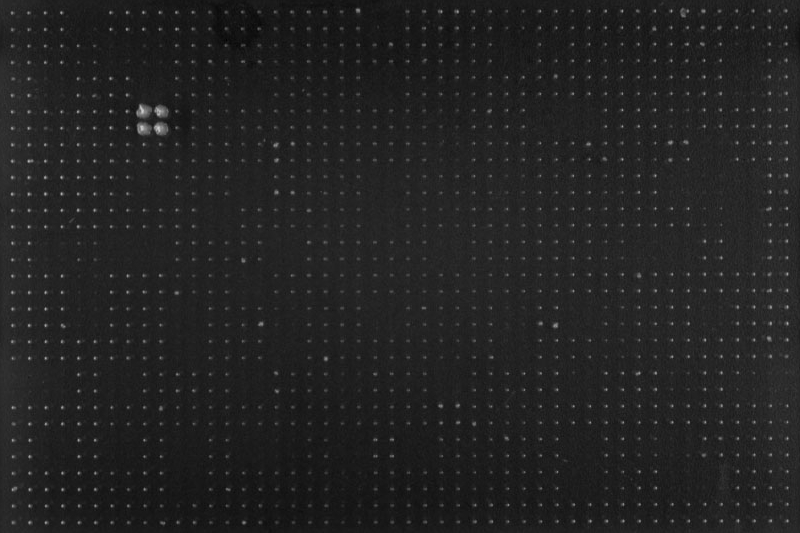

Supplement: Supplemental Material [file supp_g3.116.032607_FileS2.zip › IndividualImagesForSupplementalFile2/mel_A6-TF1-40mM3AT-after10days.png]

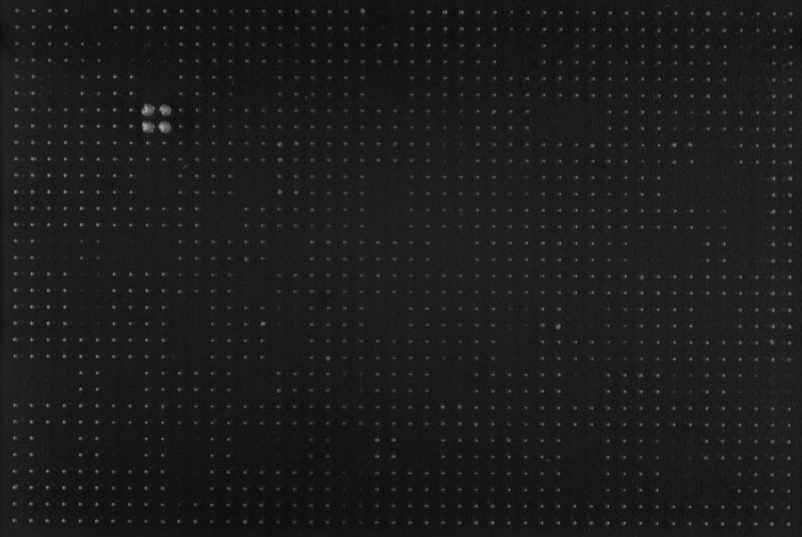

Supplement: Supplemental Material [file supp_g3.116.032607_FileS2.zip › IndividualImagesForSupplementalFile2/mel_A6-TF1-40mM3AT-after7days.1sc.png]

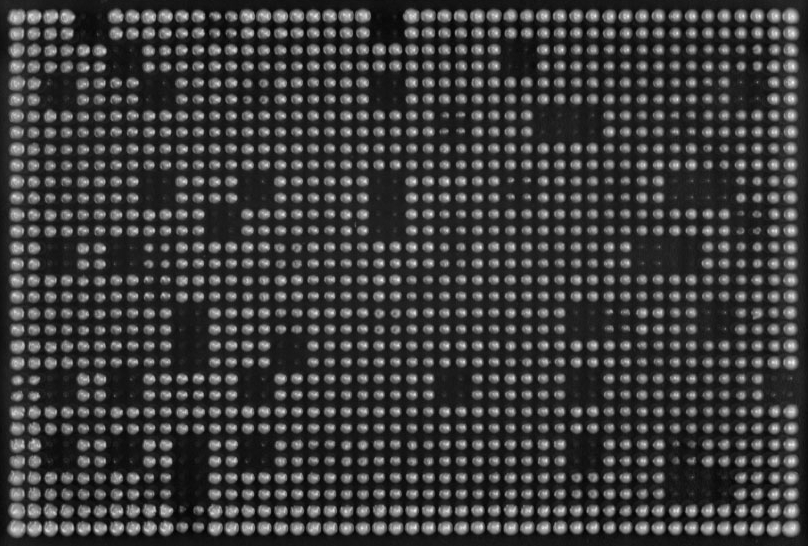

Supplement: Supplemental Material [file supp_g3.116.032607_FileS2.zip › IndividualImagesForSupplementalFile2/mel_A6-TF1-no3AT-3days-1536.1sc.png]

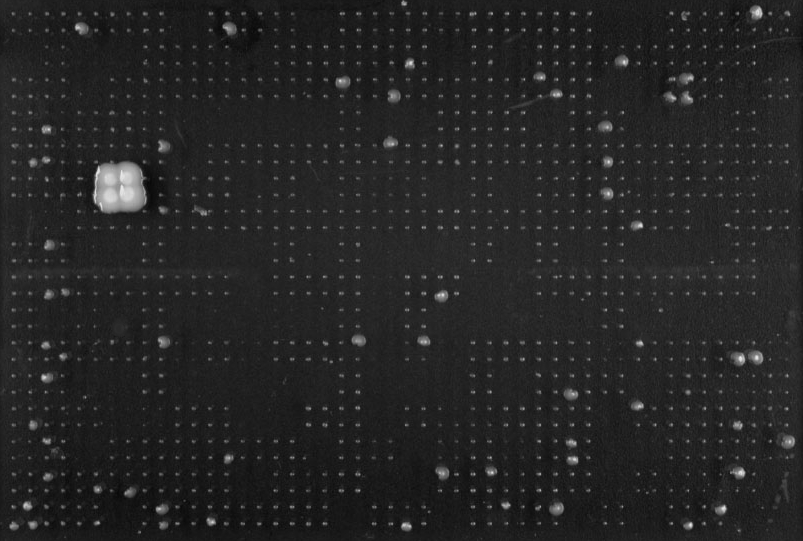

Supplement: Supplemental Material [file supp_g3.116.032607_FileS2.zip › IndividualImagesForSupplementalFile2/mel_A6-TF2-10mM3AT-after10days.png]

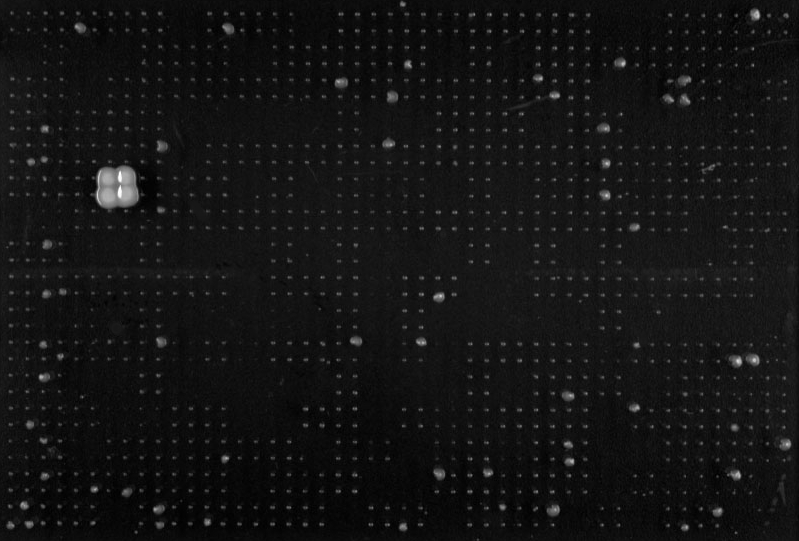

Supplement: Supplemental Material [file supp_g3.116.032607_FileS2.zip › IndividualImagesForSupplementalFile2/mel_A6-TF2-10mM3AT-after7days.1sc.png]

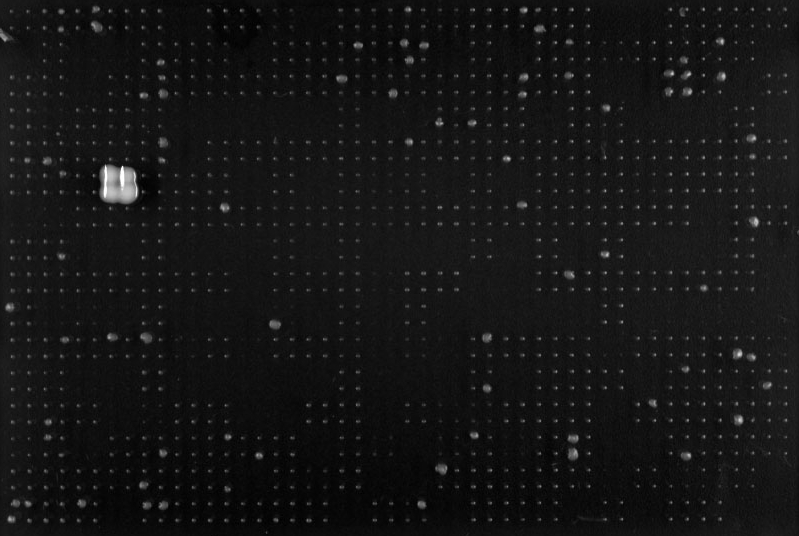

Supplement: Supplemental Material [file supp_g3.116.032607_FileS2.zip › IndividualImagesForSupplementalFile2/mel_A6-TF2-20mM3AT-after10days.png]

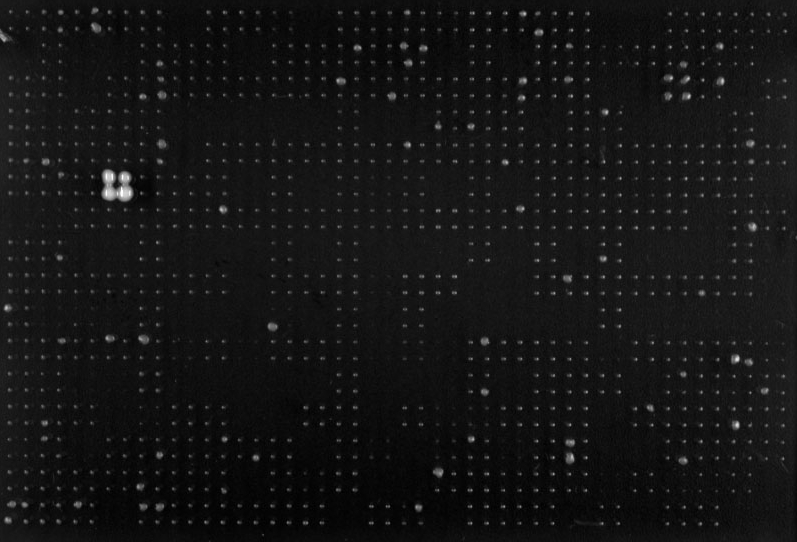

Supplement: Supplemental Material [file supp_g3.116.032607_FileS2.zip › IndividualImagesForSupplementalFile2/mel_A6-TF2-20mM3AT-after7days.1sc.png]

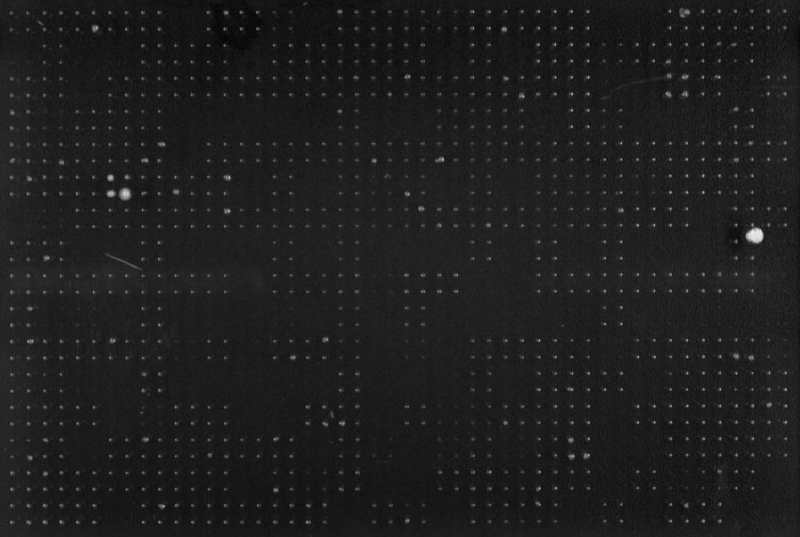

Supplement: Supplemental Material [file supp_g3.116.032607_FileS2.zip › IndividualImagesForSupplementalFile2/mel_A6-TF2-40mM3AT-after10days.png]

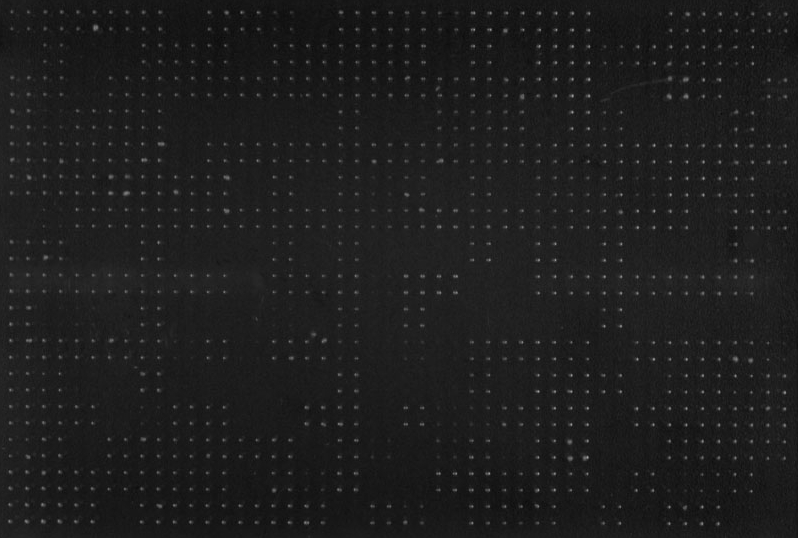

Supplement: Supplemental Material [file supp_g3.116.032607_FileS2.zip › IndividualImagesForSupplementalFile2/mel_A6-TF2-40mM3AT-after7days.1sc.png]

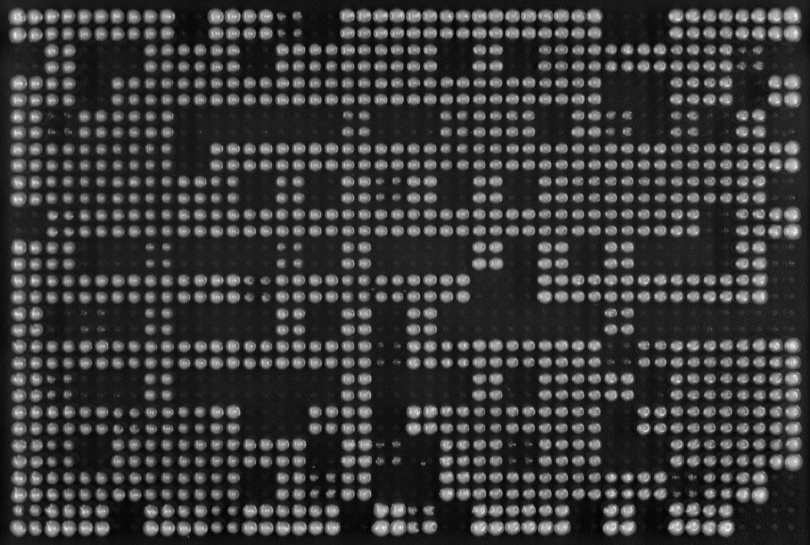

Supplement: Supplemental Material [file supp_g3.116.032607_FileS2.zip › IndividualImagesForSupplementalFile2/mel_A6-TF2-no3AT-3days-1536.1sc.png]

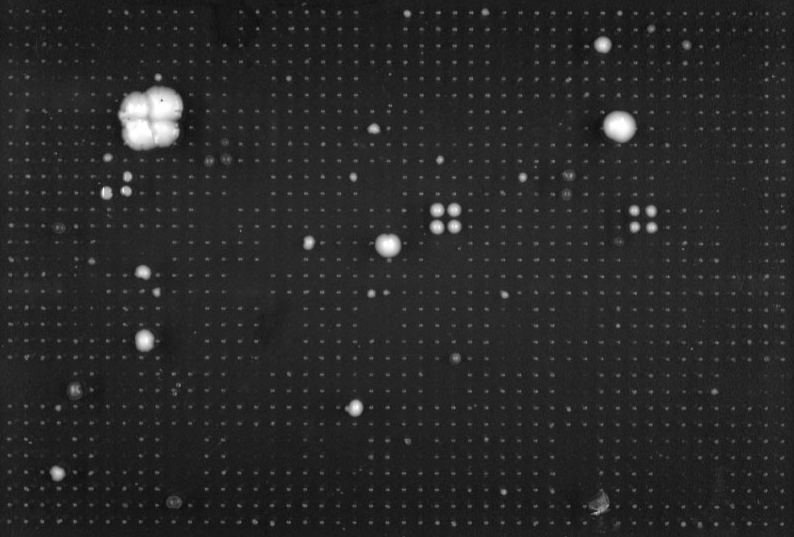

Supplement: Supplemental Material [file supp_g3.116.032607_FileS2.zip › IndividualImagesForSupplementalFile2/mel_A7-TF1-10mM3AT-after10days.png]

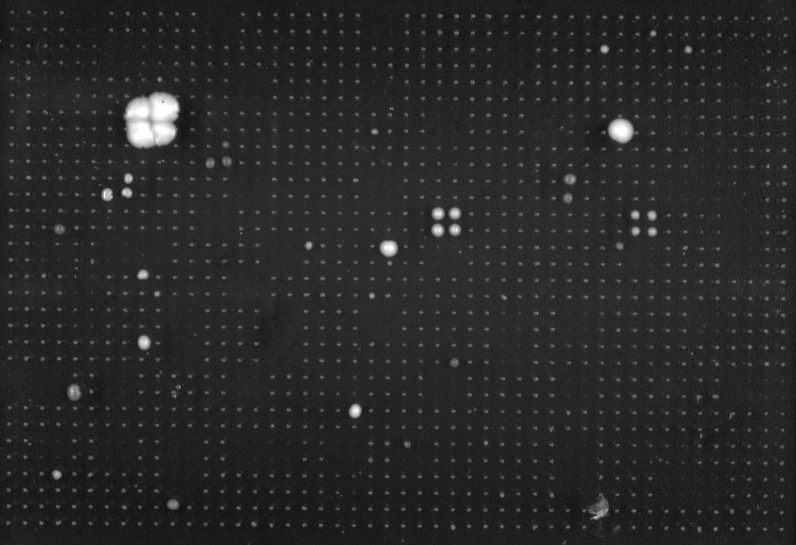

Supplement: Supplemental Material [file supp_g3.116.032607_FileS2.zip › IndividualImagesForSupplementalFile2/mel_A7-TF1-10mM3AT-after7days.1sc.png]

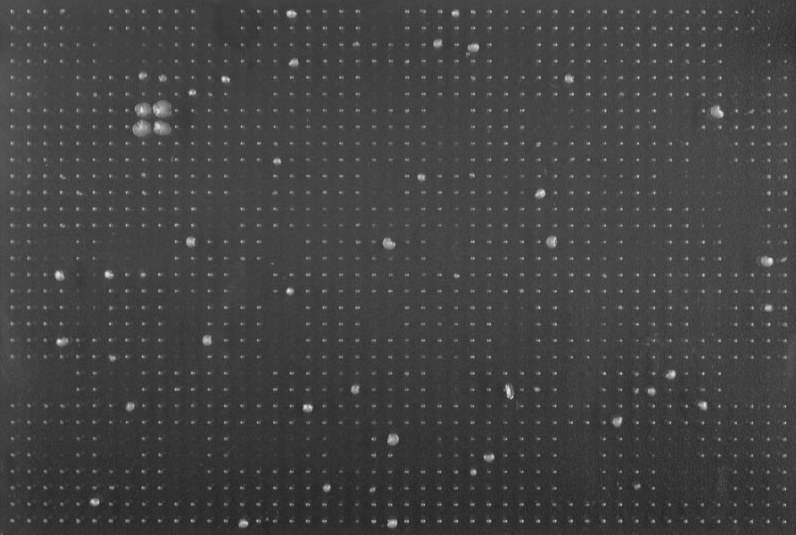

Supplement: Supplemental Material [file supp_g3.116.032607_FileS2.zip › IndividualImagesForSupplementalFile2/mel_A7-TF1-20mM3AT-after10days.png]

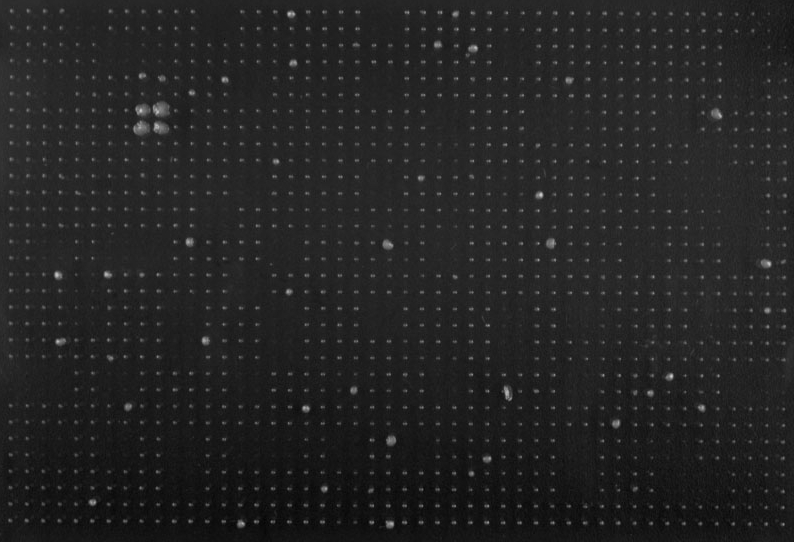

Supplement: Supplemental Material [file supp_g3.116.032607_FileS2.zip › IndividualImagesForSupplementalFile2/mel_A7-TF1-20mM3AT-after7days.1sc.png]

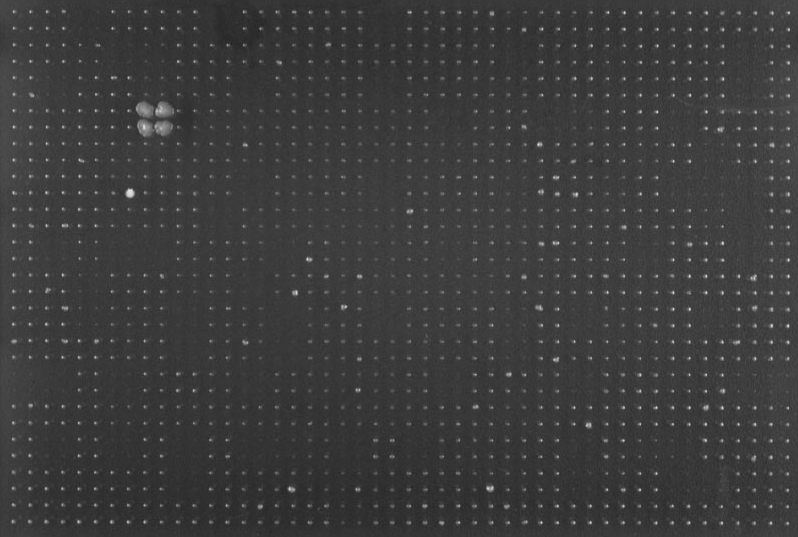

Supplement: Supplemental Material [file supp_g3.116.032607_FileS2.zip › IndividualImagesForSupplementalFile2/mel_A7-TF1-40mM3AT-after10days.png]

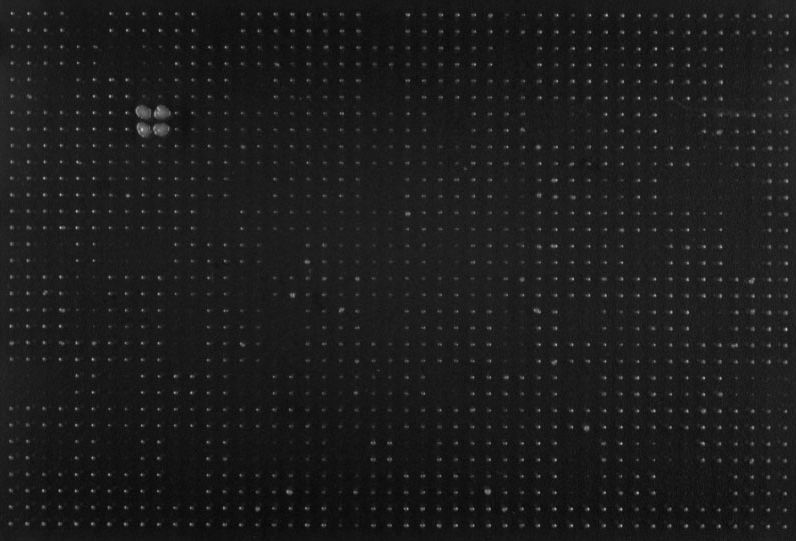

Supplement: Supplemental Material [file supp_g3.116.032607_FileS2.zip › IndividualImagesForSupplementalFile2/mel_A7-TF1-40mM3AT-after7days.1sc.png]

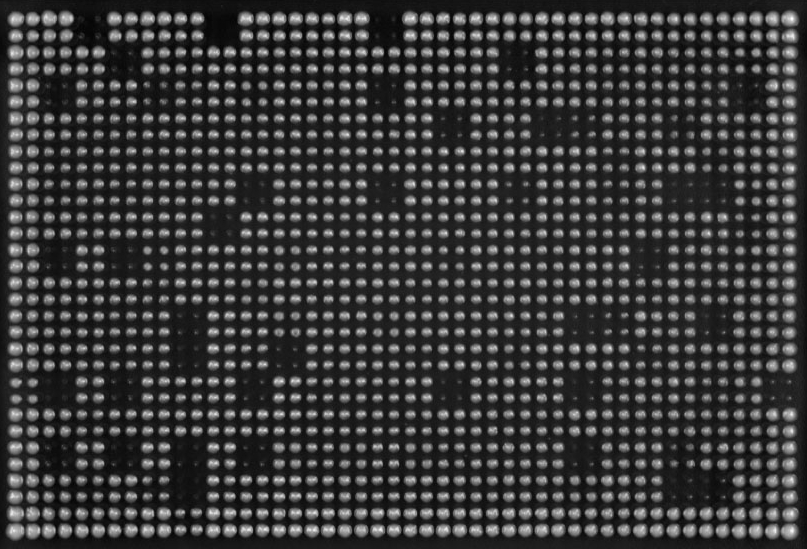

Supplement: Supplemental Material [file supp_g3.116.032607_FileS2.zip › IndividualImagesForSupplementalFile2/mel_A7-TF1-no3AT-3days-1536.1sc.png]

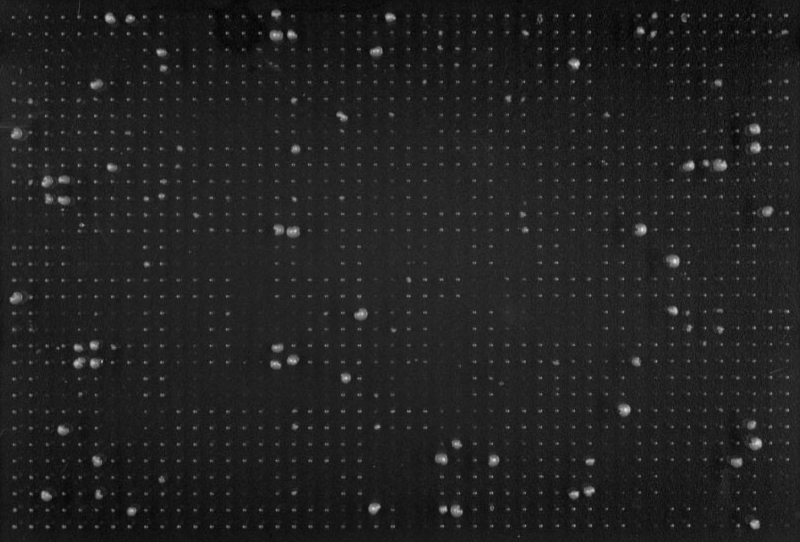

Supplement: Supplemental Material [file supp_g3.116.032607_FileS2.zip › IndividualImagesForSupplementalFile2/mel_A7-TF2-10mM3AT-after10days.png]
