# Supplementary figures and images for: Potential Direct Regulators of the Drosophila yellow Gene Identified by Yeast One-Hybrid and RNAi Screens (part 3 of 3)
Source: G3 (Bethesda). 2016 Aug 12;6(10):3419–30. doi: 10.1534/g3.116.032607 (PMC5068961; doi:10.1534/g3.116.032607)

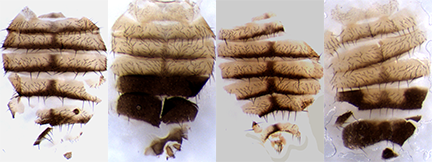

Supplement: Supplemental Material [file supp_g3.116.032607_FileS5.zip › reduced file size/AbdB_ContolFemMaleLeft_KnockdownFemMaleRight.tif]

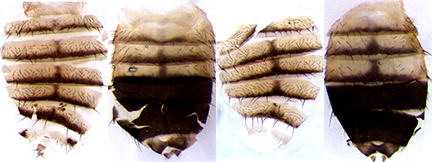

Supplement: Supplemental Material [file supp_g3.116.032607_FileS5.zip › reduced file size/ato_34929_ContolFemMaleLeft_KnockdownFemMaleRight copy.tif]

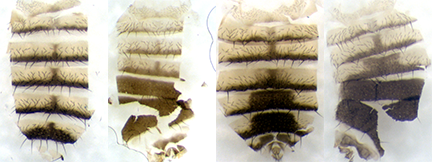

Supplement: Supplemental Material [file supp_g3.116.032607_FileS5.zip › reduced file size/bab1_ContolFemMaleLeft_KnockdownFemMaleRight copy.tif]

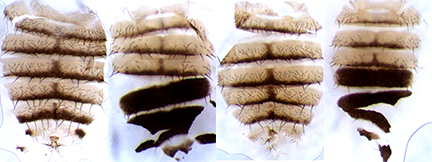

Supplement: Supplemental Material [file supp_g3.116.032607_FileS5.zip › reduced file size/BEAF32_ContolFemMaleLeft_KnockdownFemMaleRight copy.tif]

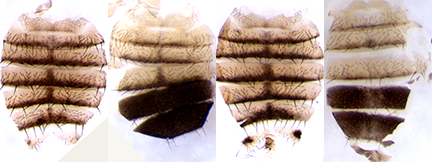

Supplement: Supplemental Material [file supp_g3.116.032607_FileS5.zip › reduced file size/brm_31712_ContolFemMaleLeft_KnockdownFemMaleRight copy.tif]

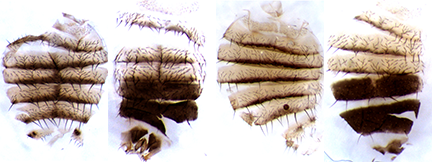

Supplement: Supplemental Material [file supp_g3.116.032607_FileS5.zip › reduced file size/C15_27649_ContolFemMaleLeft_KnockdownFemMaleRight copy.tif]

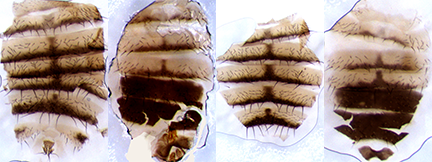

Supplement: Supplemental Material [file supp_g3.116.032607_FileS5.zip › reduced file size/CG11984_ContolFemMaleLeft_KnockdownFemMaleRight copy.tif]

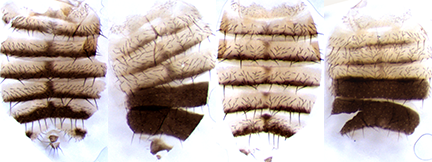

Supplement: Supplemental Material [file supp_g3.116.032607_FileS5.zip › reduced file size/CG1845_ContolFemMaleLeft_KnockdownFemMaleRight copy.tif]

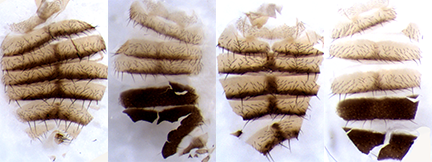

Supplement: Supplemental Material [file supp_g3.116.032607_FileS5.zip › reduced file size/CG30020_ContolFemMaleLeft_KnockdownFemMaleRight copy.tif]

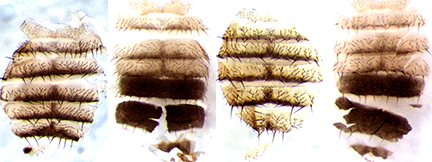

Supplement: Supplemental Material [file supp_g3.116.032607_FileS5.zip › reduced file size/Chrac14_ContolFemMaleLeft_KnockdownFemMaleRight copy.tif]

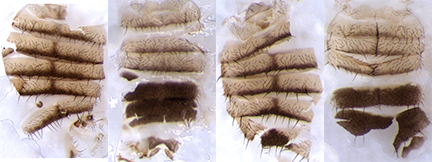

Supplement: Supplemental Material [file supp_g3.116.032607_FileS5.zip › reduced file size/dsx_ContolFemMaleLeft_KnockdownFemMaleRight copy.tif]

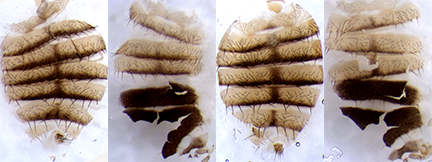

Supplement: Supplemental Material [file supp_g3.116.032607_FileS5.zip › reduced file size/Eip78C_ContolFemMaleLeft_KnockdownFemMaleRight copy.tif]

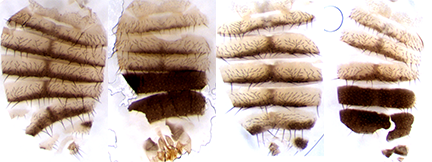

Supplement: Supplemental Material [file supp_g3.116.032607_FileS5.zip › reduced file size/fru_ContolFemMaleLeft_KnockdownFemMaleRight copy.tif]

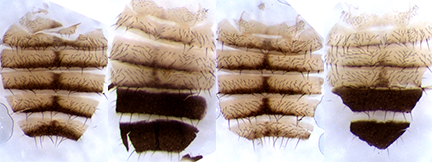

Supplement: Supplemental Material [file supp_g3.116.032607_FileS5.zip › reduced file size/hb_ContolFemMaleLeft_KnockdownFemMaleRight copy.tif]

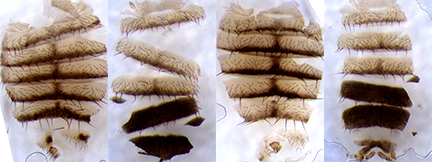

Supplement: Supplemental Material [file supp_g3.116.032607_FileS5.zip › reduced file size/Hesr_ContolFemMaleLeft_KnockdownFemMaleRight copy.tif]

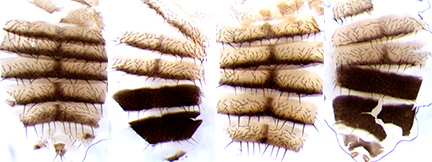

Supplement: Supplemental Material [file supp_g3.116.032607_FileS5.zip › reduced file size/Hr38_ContolFemMaleLeft_KnockdownFemMaleRight copy.tif]

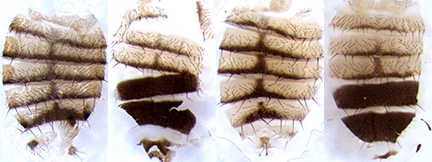

Supplement: Supplemental Material [file supp_g3.116.032607_FileS5.zip › reduced file size/Hr46_ContolFemMaleLeft_KnockdownFemMaleRight copy.tif]

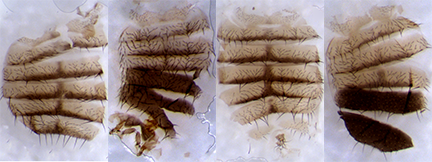

Supplement: Supplemental Material [file supp_g3.116.032607_FileS5.zip › reduced file size/Hr78_ContolFemMaleLeft_KnockdownFemMaleRight copy.tif]

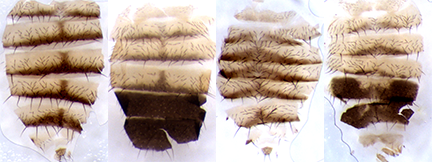

Supplement: Supplemental Material [file supp_g3.116.032607_FileS5.zip › reduced file size/jing_27024_ContolFemMaleLeft_KnockdownFemMaleRight copy.tif]

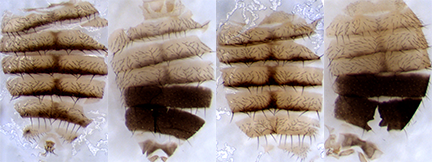

Supplement: Supplemental Material [file supp_g3.116.032607_FileS5.zip › reduced file size/lab_ContolFemMaleLeft_KnockdownFemMaleRight copy.tif]

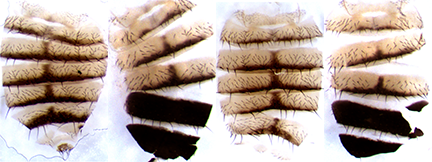

Supplement: Supplemental Material [file supp_g3.116.032607_FileS5.zip › reduced file size/Lim3_ContolFemMaleLeft_KnockdownFemMaleRight copy.tif]

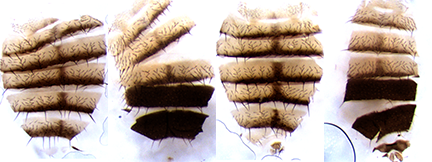

Supplement: Supplemental Material [file supp_g3.116.032607_FileS5.zip › reduced file size/Met_ContolFemMaleLeft_KnockdownFemMaleRight copy.tif]

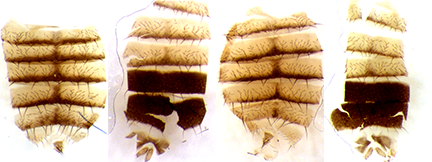

Supplement: Supplemental Material [file supp_g3.116.032607_FileS5.zip › reduced file size/noc_ContolFemMaleLeft_KnockdownFemMaleRight copy.tif]

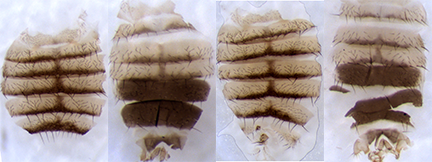

Supplement: Supplemental Material [file supp_g3.116.032607_FileS5.zip › reduced file size/pnt_ContolFemMaleLeft_KnockdownFemMaleRight copy.tif]

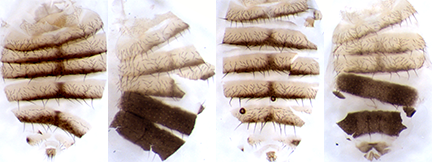

Supplement: Supplemental Material [file supp_g3.116.032607_FileS5.zip › reduced file size/sd_ContolFemMaleLeft_KnockdownFemMaleRight copy.tif]

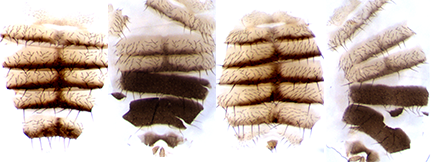

Supplement: Supplemental Material [file supp_g3.116.032607_FileS5.zip › reduced file size/sima_ContolFemMaleLeft_KnockdownFemMaleRight copy.tif]

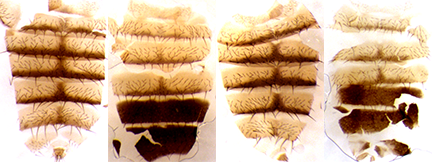

Supplement: Supplemental Material [file supp_g3.116.032607_FileS5.zip › reduced file size/sox102F_ContolFemMaleLeft_KnockdownFemMaleRight copy.tif]

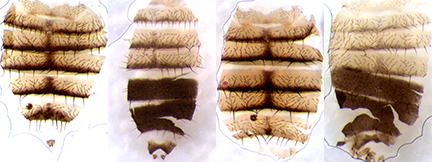

Supplement: Supplemental Material [file supp_g3.116.032607_FileS5.zip › reduced file size/SoxN_ContolFemMaleLeft_KnockdownFemMaleRight copy.tif]

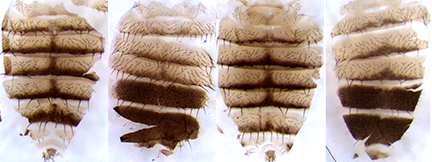

Supplement: Supplemental Material [file supp_g3.116.032607_FileS5.zip › reduced file size/Su(z)12_ContolFemMaleLeft_KnockdownFemMaleRight copy.tif]

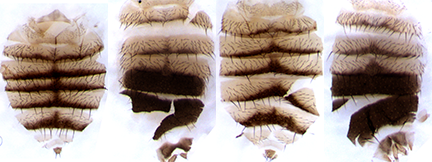

Supplement: Supplemental Material [file supp_g3.116.032607_FileS5.zip › reduced file size/Tip60_ContolFemMaleLeft_KnockdownFemMaleRight copy.tif]

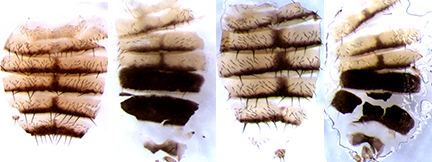

Supplement: Supplemental Material [file supp_g3.116.032607_FileS5.zip › reduced file size/ush_ContolFemMaleLeft_KnockdownFemMaleRight copy.tif]

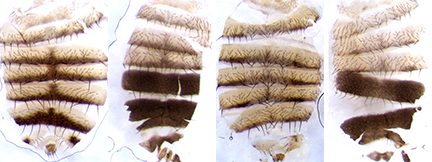

Supplement: Supplemental Material [file supp_g3.116.032607_FileS5.zip › reduced file size/vvl_ContolFemMaleLeft_KnockdownFemMaleRight copy.tif]
